# Prognostic assessment of acute ischemic stroke by systemic immune-inflammatory index: a comprehensive meta-analysis of multidimensional outcomes

**DOI:** 10.3389/fneur.2025.1594258

**Published:** 2025-10-20

**Authors:** Yanhong Jiang, Yifan Cui, Xiaojie Hu, Jiaying Lian, Xueying Qin, Xingchen Wang, Xuran Ma

**Affiliations:** ^1^The Second Clinical Medical College, Shandong University of Traditional Chinese Medicine, Jinan, China; ^2^Department of Neurology, The Second Affiliated Hospital of Shandong University of Traditional Chinese Medicine, Jinan, China; ^3^Department of Neurology, Quzhou Traditional Chinese Medicine Hospital, Quzhou, China; ^4^The First Clinical Medical College, Beijing University of Traditional Chinese Medicine, Beijing, China

**Keywords:** acute ischemic stroke, systemic immune-inflammatory index, poor prognosis, prognostic biomarker, meta-analysis

## Abstract

**Introduction:**

Our study aimed to quantify the predictive ability of the Systemic Immune-inflammatory Index (SII) for predicting the prognosis and multidimensional complications in acute ischemic stroke (AIS) patients. The primary outcome was poor prognosis, and secondary outcomes included mortality, severity, hemorrhagic transformation/symptomatic intracerebral hemorrhage, stroke-associated pneumonia/poststroke pneumonia, early neurological deterioration, post-stroke depression, progression or recurrence, and other adverse outcomes.

**Methods:**

We searched 15 databases from their establishment to 13 October 2024 and selected cohort or case-control analyses that analyzed the association of continuous or categorized SII as exposures with the above adverse outcomes of AIS populations.

**Results:**

The results showed that 78 studies with 40,682 participants were included in meta-analyses. Continuous SII values were significantly higher in poor prognosis groups than in controls (SMD = 248.13, 95% CI: 198.77 to 297.50; *p* = 0.000). Poor prognosis incidences rose with higher continuous SII values (OR = 1.004, 95% CI: 1.002 to 1.005; *p* = 0.000). More patients in High SII groups had poor prognosis (RR = 1.95, 95% CI: 1.66 to 2.28; *p* = 0.000). The risk of poor prognosis was higher in the high SII groups, though this was not statistically significant (OR = 1.007, 95% CI: 0.998 to 1.015; *p* = 0.120).

**Discussion:**

In conclusion, our study found that continuous SII and high SII were associated with poor prognosis of AIS and various complications. Given the accessibility and low cost of SII, integrating it into prognostic scores merits further research for better clinical choices.

**Systematic review registration:**

PROSPERO (CRD42024586414), https://www.crd.york.ac.uk/PROSPERO/view/CRD42024586414.

## 1 Introduction

Acute ischemic stroke (AIS), a prominent form of stroke, ranks as the primary cause of disability and mortality on a global scale (1). Given its high prevalence, there is an urgent need for a simple, accurate, and inexpensive prognostic biomarker to better predict AIS outcomes. Systemic Immune-Inflammatory Index (SII) is an inflammatory indicator calculated as Neutrophil ×Platelets/Lymphocyte, which reflects the balance between the body’s inflammatory response and immune state and the state of coagulation. There were three systematic reviews that reported on SII’s predictive value in the prognosis of AIS, but all were published early and flawed in design, with few included studies (2–4). The purpose of this study was to conduct a thorough literature search and pool data on the prognostic ability of SII for outcomes of AIS, including poor prognosis, mortality, severity, complications like hemorrhagic transformation (HT)/symptomatic intracerebral hemorrhage (sICH), stroke-associated pneumonia (SAP)/poststroke pneumonia (PSP), early neurological deterioration (END), post-stroke depression (PSD), progression/recurrence, and other complications.

## 2 Materials and methods

There were two researchers who independently conducted the entire process under MOOSE (4), with the review protocol deposited in PROSPERO (CRD42024586414). There were 15 databases searched from their establishment to 13 October 2024: PubMed, Embase, Cochrane, EBSCO, Scopus, OVID, Web of Science, CNKI, Wanfang, VIP, Sinomed, Clinical Trials, WHO-ICTRP, Chictr, and DANS EASY. AIS search subject terms included “Brain Infarction,” “Brain Ischemia,” “Cerebral Arterial Diseases,” “Cerebral Infarction,” “Cerebrovascular Disorders,” “Stroke,” and free terms included 122. SII terms included six terms (Search criteria, strategies, and results as shown in Supplementary material 1).

After eliminating duplicate reports, the remaining studies’ titles and abstracts were screened to assess their appropriateness for inclusion. Subsequently, the previously selected papers were evaluated for eligibility, data obtained, and bias risk evaluated by the Newcastle-Ottawa Scale (NOS) using the full text. Another two researchers independently conducted the abovementioned processes, and any disagreements were resolved by consulting a third guide researcher.

Eligible articles were cohort or case–control analyses analyzing the relationship between SII and AIS adverse outcomes, including poor prognosis, mortality, severity, and complications such as HT/sICH, SAP/PSP, END, PSD, progression/recurrence, and others. The inclusion criteria were as follows: 1. Population: Patients of AIS and its complications (any diagnostic criteria); 2. Required data: Continuous SII value of poor prognosis/death/mild severity/HT/SAP/END/PSD/progression or recurrence/other complications groups versus the corresponding control groups; sample size of outcomes’ events, adjusted odds ratio (aOR)/adjusted hazard ratio (aHR) of outcomes, and National Institute of Health Stroke Scale (NIHSS) in High SII groups versus Low SII groups; SII cut-off values and area under curve (AUC) of receiver operating characteristic (ROC) curves. 3. Exclusion criteria were as follows: duplicate publications, obviously incorrect data, mismatched research types, and low quality (NOS ≤ 4) (When studies provided sample sizes of outcomes’ events for high- and low-SII groups, HIGH SII was defined as the highest SII group, and LOW SII was the sum of the other groups. For aOR/aHR or NIHSS data, HIGH SII was defined as the highest SII group, and LOW SII was the lowest SII group).

We assessed the association between SII and AIS adverse outcomes using mean difference (MD), Relative Risk (RR), and pooled aOR/aHR. Using Stata 14.0, we considered a *p-*value < 0.05 significant, quantified heterogeneity with *I^2^* and *p* value of Cochran’s *Q* statistics, applied the random-effects model for high heterogeneity, and checked for bias with funnel plots and Begg/Egger tests.

## 3 Results

### 3.1 General results

**Figure 1:**
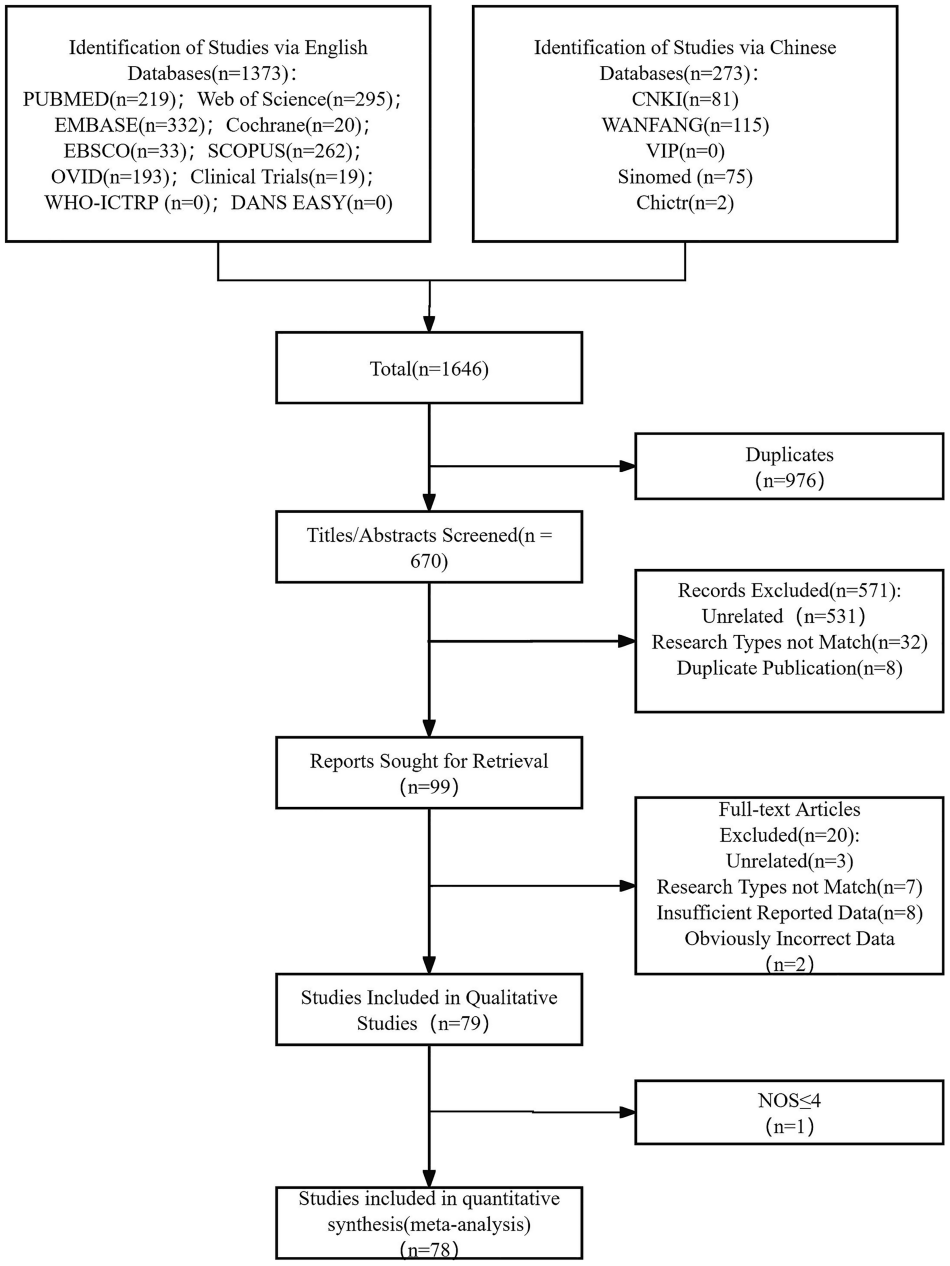
The search and screening process.

Literature search and studies included the initial search, which resulted in 1646 total studies, 670 studies that remained to be screened after removing duplicates, and 99 studies that remained for full-text assessment. Finally, 79 studies (1, 5–82) remained to be included in the systematic review, and 78 studies remained to be included in the meta-analysis (1, 5–33, 35–82) except Wang SN 2024 (34). Details of the process are shown in Figure 1. A summary of the main characteristics of the 78 studies is presented in Table 1; the rating of the quality of the evidence by NOS is presented in Table 2.

**Table 1:** Characteristics of studies investigating the relationship between SII and AIS.

No.	References	Study design	NOS	Region	Population	Type of AIS	Entry time	Participants (M/F)	Age-year (Mean ± SD)/[Median(IQR)]	Medical & medication history	Blood sampling	Followed-up	Outcomes
Cohort studies													
1	Wang N 2024 (13)	R-S	9	China	Changhai Hospital of Naval Medical University	AIS with IVT	2016.01–2020.12	466 (291/175)	65.5	①②③⑤⑥	Before IVT (Within 4.5 h of Symptom Onset)	90d	ACDE
2	Zhang LL 2024 (15)	R-S	9	China	the First Affiliated Hospital of Soochow University	AIS with Atherosclerotic Plaque in Responsible Carotid Artery	2020.01–2022.06	202 (147/55)	Vulnerable groups 65.13 ± 10.53/Stable groups 64.57 ± 11.28	①②③④⑮⑱	Within 24 h of Admission	1mos	J
3	Wei 2024 (16)	R-S	9	China	Second Hospital of Tianjin Medical University	AIS with IVT	2019.03–2021.05	221 (138/83)	68.0 ± 12.1	①②③④⑤⑥⑮⑯	Before The Bolus of IVT	3mos	ABCDE
4	Zhang MK 2024 (32)	R-S	9	China	Xuan Wu Hospital, affiliated to Capital Medical University	AIS with EVT & fDNI	2017.01–2020.04	352 (250/102)	DNI groups 60.89 ± 11.63/Non-DNI groups 64.81 ± 11.85	①②③④⑤⑥	Before EVT	90d	J
5	Yang Y 2024 (5)	R-S	8	China	Beijing Friend-ship Hospital, Capital Medical University	AIS with ICA severe stenosis and SAP	2020.1–2023.6	342 (171/171)	65.2 ± 10.2/66.3 ± 11.1	①②③④⑤⑥⑦⑮⑯⑱⑲	The Next Morning (5:00 a.m.) after Admission	120d	BC
6	Cao 2024 (8)	R-S	8	China	Xuanwu Hospital of Capital Medical University	Anterior Circulation AIS-LVO with EVT	2018.12–2022.12	482 (323/159)	65 (56–72)	①②③④⑤⑥⑮⑯	Admission or the first day post-EVT	90d	AD
7	Arslan 2024 (6)	R-S	7	Turkey	Istanbul Kanuni Sultan Süleyman Training and Research Hospital	Critical AIS in ICU	2020–2022	198 (95/103)	70 (56–86)	①②④⑤⑧	NR	28d	AB
8	Zhu 2024 (7)	R-S	7	China	Nantong Third People’s Hospital	AIS NOT EVT or IVT	2019.09–2024.02	306 (191/115)	FPG groups 68.761 ± 10.763, PPG groups 75.327 ± 8.911	①②③④⑤⑥⑫⑮⑯⑱	Within 1 h of Admission	30d	AG
9	Zhao 2024 (17)	R-S	7	China	Wuxi People’s Hospital	AIS with IVT	NR	197 (125/72)	FPG 68.18 ± 10.09/PPG 67.69 ± 8.75	①②⑤	Within 4.5 h of Symptom Onset	NR	A
10	Guoqing 2024 (18)	R-S	7	China	People’s Hospital of Xinjiang Uygur Autonomous Region	AIS with IVT	2021.06–2023.06	122 (65/57)	58 (54, 63)	①②	Before The Bolus of IVT	6mos	A
11	Ma L 2024 (21)	R-S	7	China	the Second Affiliated Hospital of Anhui Medical University	ACI with IVT	2021.09–2023.09	199 (130/69)	62.96 ± 13.00	①②⑤⑥⑮⑱	Before IVT	3mos	AEG
12	Huang H 2024 (29)	R-S	7	China	the First People’s Hospital of Suqian	Minor Stroke Due to Anterior Circulation AIS-LVO	2021.11–2023.12	132 (85/47)	68 (58–77)	①②③④⑤⑥⑮⑯⑱⑲㉒	340 (228 ~ 572)Min after Onset	24 h	E
13	Misirlioglu 2024 (10)	R-S	6	Turkey	Gaziosmanpasa Education and Research Hospital	AIS	2019.01–2023.06	1,350 (710/640)	64.38 ± 16.43	①②③④⑤	Within 24 h of Stroke Onset	NR	B
14	Mengting 2024 (20)	R-S	6	China	Xishan People’s Hospital of Wuxi	ACI with IVT	2022.01–2023.12	174 (111/63)	FPG groups 68 (57, 76)/PPG groups74 (66, 81)	①②③④⑤⑥⑮⑱	Before and 24 h after IVT	Discharge	A
15	Zhouquan 2024 (23)	R-S	6	China	the Second People’s Hospital of Chengdu	AIS with IVT	2022.03–2023.03	213 (125/88)	67.5 ± 20.5	①②⑤	Admission	3mos	A
16	Jiaxiang 2024 (25)	R-S	6	China	Nanjing Drum Tower Hospital	AIS with IVT	2020.01–2022.12	185 (104/81)	END groups 80 (70, 84)/Non-END groups 73 (66, 80)	①②③⑤⑥	NR	3mos	E
17	Zhang J 2024 (27)	R-S	5	China	The Affiliated Hospital of Chengde Medical College	AIS	2023.01–2023.12	115 (81/34)	PIS groups 61.83 ± 10.89/Non-PIS groups 64.06 ± 9.92	①②④⑤	Within 24 h of Admission	7d	J
18	Haimei 2024 (30)	R-S	5	China	Taizhou People’s Hospital	AIS	2022.01–2022.12	259 (159/100)	SAP groups 71.00 (61.00, 81.00)/Non-SAP groups 70.00 (58.00, 77.25)	①②④⑤	NR	7d	F
19	Lijun 2024 (31)	R-S	5	China	The First Affiliated Hospital of Naval Medical University	AIS	2022.08–2022.12	80 (58/22)	27–84	①②③④⑥	The Day After Admission	90d	G
20(1)	Huang SW 2024 (1) (1)	R-M	8	China	the First Affiliated Hospital of Wenzhou Medical University	AIS without IVT	2020.1–2020.12	1,268 (835/433)	67 (59–76)	①②④⑤⑥	Within 24 h of Admission	1y	ABJ
20(2)	Huang SW 2024 (1) (2)	R-M	8	China	the Third Affiliated Hospital of Wenzhou Medical University	AIS without IVT	2020.1–2020.12	536 (341/195)	69 (60–78)	①②④⑤⑥	Within 24 h of Admission	1y	ABJ
20(3)	Huang SW 2024 (1) (3)	R-M	8	China	Both	AIS without IVT	2020.1–2020.12	650 (391/259)	75.00 (68.00–81.00)	①②④⑤⑥	Within 24 h of Admission	1y	ABJ
21	Lee 2024 (12)	P-S	8	Korea	Soonchunhyang University School of Medicine	AIS	2019.01–2021.12	697 (405/292)	4 SII groups:69.4 ± 13.3/67.1 ± 13.1/68.8 ± 13.8/71.4 ± 14.1	①②⑤⑨⑩⑭⑰⑱⑲	Within 1 h after Admission	7d	ACEI
22	Cheng 2024 (9)	P-S	8	China	the First People’s Hospital of Yancheng	AIS	2022.01–2023.03	332 (203/129)	68 (58–76)	①②④⑤⑬	The Next Morning	3mos	J
23	Hao 2024 (11)	P-S	7	China	People’s Hospital of Zhengzhou University	AIS with IVT	2020.01–2022.08	121 (78/43)	63.8 ± 12.9	①②④⑥⑬⑲⑳	Within 24 h after Ischemic Stroke Onset	Discharge	J
24	Chen GJ 2024 (14)	P-M	8	China	111 hospitals(Clinical trials NCT03370939)	AIS with EVT	2017.11–2019.03	1,002 (660/342)	65 (55–72)	①②④⑤	The First Test on Admission & before EVT	90d	AC
25	Fernández-Garza 2023 (35)	R-S	9	Mexico	University Hospital “Dr. José Eleuterio González”	AIS	2018.01–2019.06	145 (97/48)	61.5 ± 12.75	①②③⑥⑲	Within 24 h of Admission	90d	AG
26	Ma 2023 (37)	R-S	9	China	Jiangsu Province Hospital of Chinese Medicine	AIS with IVT	2019.09–2022.12	190 (122/68)	70.389 ± 11.675	①②④⑤⑥⑮⑯⑱㉒㉓	Within 24 h of Admission	3mos	ABI
27	Zhao 2023 (38)	R-S	8	China	Hebei general hospital	AIS with IVT	2017.09–2022.08	281 (168/113)	66 (56–73)	①②③④⑤⑥	Before IVT	3mos	AE
28	Hu 2023 (39)	R-S	8	America	MIMIC-IV(the Beth Israel Deaconess Medical Center)	AIS Admitted to the ICU	2008–2019	463 (221/242)	71.68 ± 16.29	②④⑦⑧⑩⑪	NR	Discharge	B
29	Zhang 2023 (40)	R-S	8	China	Changhai Hospital	AIS with EVT	2019.01–2019.12	248 (160/188)	67.19 ± 11.47	①②③④⑤㉔	On Admission	90 ± 14d	F
30	Chu 2023 (41)	R-S	8	China	Minhang Hospital of Fudan University	Mild AIS with IVT	2017.01–2022.05	240 (81/159)	66.00 (60.00–73.35)	①②⑤	Before IVT	3mos	A
31	Gao 2023 (54)	R-S	8	China	Huai’an First People’s Hospital	AIS with IVT	2019.07–2022.07	352 (240/112)	66.46 ± 12.00	①②⑤⑥	The Morning after Admission	36 h	D
32	Wang S 2023 (36)	R-S	7	China	the First Affiliated Hospital of Soochow University	AIS with IVT	2017.01–2022.08	717 (485/232)	68 (58–75)	①②③⑤⑥⑮⑯⑰	NR	3mos	A
33	Zhou 2023 (46)	R-S	7	China	The Affiliated Hospital of Guilin Medical College	AIS	2020.01–2020.12	208 (143/65)	63.3 ± 11.3	①②③④⑤	Within 24 h of Admission	3mos	AC
34	Xiao 2023 (43)	R-S	6	China	Guangzhou First People’s Hospital	AIS with PFO	2021.02–2021.12	100 (78/22)	PFO groups 50.48 ± 8.86/Non-PFO groups 54.00 ± 10.30	①②③	NR	NR	J
35	Dan-dan 2023 (44)	R-S	6	China	Affiliated Hospital of Xuzhou Medical University	Elderly AIS with IVT	2019.08–2022.02	347 (228/119)	60–93 (70.12 ± 7.71)	①②⑤⑥	NR	3mos	AD
36	Shao 2023 (47)	R-S	6	China	Lianyungang Second People’s Hospital	Acute Lacunar Infarction	2021.01–2022.06	172 (112/60)	BG-EPVS mild groups 63.35 ± 11.46/BG-EPVS Moderate-to-Severe groups 69.16 ± 10.13	①②	The Morning after Admission	7d	J
37	Wang X 2023 (48)	R-S	6	China	the First Affiliated Hospital of Shihezi University Medical College	AIS with EVT	2019.01–2022.12	682 (481/201)	65.00(55.00, 76.00)	①②	NR	90d	A
38	Song 2023 (49)	R-S	6	China	Wafangdian Third Hospital	ACI	2021.01–2022.05	310 (200/110)	62.58 ± 10.27	①②③④⑤	Within 24 h of Admission	1mos	HJ
39	Wang YL 2023 (50)	R-S	6	China	Jianping County Hospital of traditional Chinese medicine	ACI with IVT	2021.05–2022.09	100 (40/60)	64.24 ± 9.22	①②④⑥	NR	3mos	A
40	Liu HT 2023 (51)	R-S	6	China	Northern Jiangsu People’s Hospital	AIS with AF & IVT	2018.10–2022.11	514 (285/229)	AF-S groups 73.2 ± 10.2/Non-AF-S groups 66.1 ± 11.1	①②④⑤⑥⑮⑯	Before IVT; Morning of The Second Day after Admission	90d	ADJ
41	Dong 2023 (53)	R-S	6	China	Baoji Municipal Central Hospital	AIS-LVO with EVT	2017.12–2022.06	219 (122/97)	39–83 (61 ± 9)	①②③④⑤	Immediately after Admission	90d	J
42	Huixin 2023 (55)	R-S	6	China	Xuanwu Hospital	ALVOS with EVT	2019.01–2021.01	426 (282/144)	65 (57, 74)	①②③④⑤⑥	Before EVT	90d	A
43	Liu YY 2023 (56)	R-S	6	China	The Fifth Affiliated Hospital of Zhengzhou University	AIS	2021.03–2022.10	22 (NR)	NR	①②④⑥⑮⑱	The Morning after Admission	90d	A
44	Su 2023 (57)	R-S	6	China	Nanchong Mental Health Center of Sichuan Province	AIS with IVT	2021.01–2022.08	Model 272 (143/129); Verification 112 (54/58)	63.02 ± 11.27	①④	NR	3mos	J
45	Lin 2023 (42)	P-S	7	China	Shunde Hospital of Southern Medical University	AIS	2022.01–2022.09	177 (121/56)	FPG groups 63.04 ± 12.26/PPG groups 63.17 ± 13.44	①②③④⑥㉔	Within 24 h On The Day of Admission	90d	AGJ
46	Wang ZT 2023 (52)	P-S	7	China	the First Affiliated Hospital of China Medical University	AIS with IVT	2020.09–2022.09	324 (219/105)	65 (58, 71)	①②④⑤	Before IVT	90d	AE
47	Li 2023 (45)	P-S	6	China	Xianyang Hospital of Yan’an University	Anterior Circulation AIS	2020.10–2022.10	110 (83/27	62.03 ± 10.54	NR	Within 24 h	3mos	A
48	Zhang 2022 (59)	R-S	9	China	the First People’s Hospital of Yancheng	AIS with Carotid Atherosclerotic Plaque	2020.06–2021.03	131 (98/33)	61.86 ± 12.37	①②④⑥⑮⑱	Within 24 h of Admission	1mon	CJ
49	Liu 2022 (69)	R-S	9	China	Yantai Yuhuangding Hospital	AIS	2020.08–2021.08	266 (160/106)	Mild groups 64.2 ± 10.0/Moderate-to-severe groups 66.2 ± 12.1	①②⑤⑮⑯	Within 24 h after Onset	90d	ADG
50	Wu 2022 (61)	R-S	8	America	MIMIC-IV(the Beth Israel Deaconess Medical Center)	AIS	2008–2019	1,181 (600/581)	69.1 ± 15.6	①②③④⑤⑧⑩⑪⑫⑭⑮⑯㉔	The First Test Results At Icu.	30d&90d	B
51	Yang 2022 (64)	R-S	8	China	West China Hospital	AIS-LVO with EVT	2017.01–2021.01	379 (199/180)	71 (58–78)	①②③⑤⑰⑱	Immediately Upon Arrival At The Emergency Room	NR	CD
52	Li 2022 (58)	R-S	7	China	Huizhou Central People’s Hospital	LAO-AIS after EVT	2020.01–2022.01	173 (118/55)	56.9 ± 8.9	①②③④⑤	In The Emergency Department Or Within 1D of Admission	NR	J
53	Wenli Z 2022 (71)	R-S	7	China	Nanjing Municipal First Hospital	Acute Stroke with EVT	2018.01–2020.06	88 (52/36)	67.39 ± 28.21	①②③⑤	NR	3mos	AJ
54	Lin 2022 (63)	R-S	6	China	NR	AIS	2017.01–2019.06	526 (277/249)	Definite AF groups 68.08 ± 12.16/Non-AF groups 78.61 ± 9.65	①②④⑥⑲⑳	During Hospitalization, after Fasting For At Least 12 h	Discharge	J
55	Zhou 2022 (65)	R-S	6	China	The Affiliated Hospital of Guilin Medical University	AIS	2020.01–2020.12	208 (143/65)	63.3 ± 11.3	①②③④⑤	Within 24 h	3mos	A
56	Ma 2022 (68)	R-S	6	China	Urumqi Friendship Hospital	AIS with IVT	2020.05–2021.08	63 (33/30)	65.0 ± 11.0	NR	Before IVT	90d	A
57	Laiyun Z 2022 (70)	R-S	6	China	The Affiliated Hospital of Xuzhou Medical University	Young ACI	2019.03–2021.03	182 (152/30)	FPG groups 40.00 (35.00, 44.00)/PPG groups 39.00 (34.00, 43.00)	①②	Within 24 h of Admission	3mos	AG
58	Chen 2022 (66)	R-S	5	China Taiwan	Taipei Tzu Chi Hospital	AIS	2011.01–2021.04	3,402 (72 IHIS+3,330 OHIS) (1959/1443)	IHIS groups 75.3 (65.6–81.9)/OHIS groups 71.8 (61.7–81.5)	①②③④⑤⑥⑦⑩	Emergency Department Arrival/During Acute Attack of Stroke at Ward	Discharge	AB
59	Adiguzel 2022 (67)	R-S	5	Turkey	Hacettepe University Neurology Intensive Care and Stroke Unit	Severe AIS(NIHSS>10)	2019–2021	205 (85/120)	71 ± 15	①②⑤⑧⑨⑰㉔	Within The First 12H after Stroke Onset	Discharge/3mos	ABFJ
60	Ji 2022 (62)	R-M	8	China	Jinling Hospital & Yijishan Hospital	Anterior Circulation LVOS with EVT	2014.01–2018.12/2015.09–2021.07	675 (402/273)	67.1 ± 11.4	①②⑤	Within The First 24 h after Admission	90d	AJ
61	Wang 2022 (60)	P-M	8	China	201 hospitals(CNSR-III)	AIS	NR	9,107 (6343/2764)	61.9 ± 11.1	①②③④⑤⑥	NR	90d&1y	ABI
62	Zhong 2021 (79)	R-S	8	China	the First Affiliated Hospital of Kunming Medical University	AIS	2017.02–2020.04	238 (131/107)	FPG groups 60.47 ± 13.25/PPG groups 68.86 ± 13.19	①②③④⑤⑥⑧⑲㉔	Within 24 h of Admission	3mos	AFG
63	Weng 2021 (75)	R-S	8	China	the Third Affiliated Hospital of Wenzhou Medical University	AIS with IVT	2016.02–2019.04	216 (136/80)	68.5 (59.25–76)	①②③④⑤⑥	Within 24 h after Admission	3mos	ACG
64	Wei 2021 (76)	R-S	8	China	the General Hospital of the Eastern Theater Command	AIS	2017.07–2017.12	116 (87/29)	62.09 ± 12.42	NR	AIS groups Admission/Control groups Fasted For More Than 12 h	2y	IJ
65	Li LH 2021 (74)	R-S	7	China Taiwan	Taipei Veterans General Hospital	AIS within 3 h	2016.01–2018.12	277 (157/120)	73.2 ± 13.4	②③④	Emergency Department Arrival	1y	J
66	Cheng 2021 (77)	R-S	6	China	The Affiliated Hospital of Xuzhou Medical University	AIS	2020.01–2020.12	305 (200/105)	SAP groups 75.77 ± 10.19//Non-SAP groups 61.68 ± 12.31	①②③④⑤⑥㉑㉔	Within 24 h of Admission	7d	F
67	Yi 2021 (73)	R-M	7	Korea	Soonchunhyang University Bucheon Hospital & St. Vincent’s Hospital	LAO-AIS with ET	2015.01–2020.09	440 (260/180)	FPG groups 68.0 (13.4)/PPG groups 72.6 (11.7)	①②③④⑤⑥	On Admission	3mos	ACDJ
68	Hu 2021 (72)	P-S	9	China	the First Affiliated Hospital of Wenzhou Medical University	AIS	2014–2017	432 (272/151)	62.58 ± 10.27	①②③④	The Morning after Admission, 05:00–08:00	1mon	CHJ
69	Wei 2021 (76)	P-S	6	China	Affiliated Beijing Shijitan Hospital of Capital Medical University	ACI	2018.03–2019.02	220 (137/83)	60 ~ 93 (73.86 ± 8.58)	①②③④⑤⑥⑧	Within 24 h of Admission	Discharge	F
70	Zhao 2020 (81)	R-S	8	China	Subei People’s Hospital of Jiangsu Province	ACI	2019.01–2019.07	140 (84/56)	68.20	①②④	Within The First 24 h after Admission.	0.5y	A
71	Chu 2020 (82)	R-S	6	China Taiwan	Taipei Tzu Chi Hospital	AIS	2010.05–2020.02	2,543 (1469/1074)	70.8 ± 13.5	①②③④⑥⑦⑩	Arrival In The Emergency Room	At Discharge	AJ
72	Ceng 2020 (80)	P-S	9	China	the First Affiliated Hospital of Zhengzhou University	AIS	2015.01–2017.12	SAP 1155 (NR);3 Month 1,106 (NR);1 Year 1,074 (721/434)	Non-SAP groups 59.51 ± 12.30/SAP groups 65.65 ± 13.22	①②③④⑤⑥	Within 24 h	3mos&1y	ABF
Case–control studies													
73	Dong 2024 (28)	R-S	8	China	Baoji Central Hospital	AIS	2019.02–2021.02	307 (159/148)	PSD groups 59.52 ± 10.04/Non-PSD groups 61.76 ± 9.96	①②③④⑤	Early Morning after Admission (05:00 ~ 08:00)	30d	H
74	Zheng 2024 (33)	R-S	6	China	The Affiliated Hospital of Putian University	Massive Cerebral Infarction within 48 h	2019.01–2021.11	82 (52/30)	FPG groups 68 (61.5, 80.5)/PPG groups 70 (57.5, 76)	①②⑤⑥	Within 24 h of Admission	Discharge	ADFJ
75	Zhou 2024 (19)	R-S	6	China	Wujin Hospital, Affiliated to Jiangsu University	AIS	2020.01–2022.12	238 (161/77)	SAP groups 77.57 ± 8.69/Non-SAP groups 76.57 ± 9.36	①②④	Within 24 h of Admission	7d	F
76	Tianlu 2024 (24)	R-S	6	China	the First Affiliated Hospital of Harbin Medical University	AIS	2020.01–2023.06	236 (143/93)	NR	①②㉑	NR	7d	F
77	Yu 2024 (77)	R-S	6	China	China-Japan Union Hospital of Jilin University	AIS with EVT	2021.01–2023.08	150 (103/47)	68 (59, 72)	①②④⑤⑥	NR	NR	D
78	Niu 2024 (26)	R-S	5	China	Lijin County Central Hospital	AIS with IVT	2021.07–2023.07	150 (83/67)	HT groups 49.63 ± 9.52/Non-HT groups 50.89 ± 9.66	⑭	The Next Morning	NR	D

ACI, Acute Cerebral Infarction; AF, Atrial fibrillation; AF-S, Atrial fibrillation Stroke; AIS, Acute Ischemic Stroke; AIS-LVO, Acute Ischemic Stroke with Large Vessel Occlusion; ALVOS, Acute Large Vessel Occlusive Stroke; BG-EPVS, Basal Ganglia-Enlarged Perivascular Spaces; BG-EPVS, Basal Ganglia Region Enlarged Perivascular Spaces; CNSR-III, China National Stroke Registry III; CSO-EPVS, Central Semi-ovale Region Enlarged Perivascular Spaces; DNI, Delayed Neurological Improvement; END, Early Neurological Deterioration; EVT, Endovascular Treatment; FPE, First Pass Effect; FPG, Favorable Prognosis groups; HT, Hemorrhagic Transformation; ICA, Internal Carotid Artery; IHIS, In-hospital Ischemic Stroke; IS, Ischemic Stroke; LAO-AIS, Large Artery Occlusion-Acute Ischemic Stroke; LAA, Large Artery Atherosclerosis; LVOS, Large-vessel Occlusive Stroke; MCE, Malignant Cerebral Edema; MIMIC-IV, Medical Information Mart for Intensive Care-IV; MT, mechanical thrombectomy; NOS, Newcastle–Ottawa Scale; NIHSS, National Institutes of Health Stroke Scale; NR, Not Reported; OHIS, Out-of-hospital Ischemic Stroke; PCI, Progressive Cerebral Infarction; PFO, Patent Foramen Ovale; PPG, Poor Prognosis groups; PSCI, Post-stroke Cognitive Impairment; PSD, Post-stroke Depression; PSP, Poststroke Pneumonia; PIS, Progressive Ischemic Stroke; R, Retrospective; SAP, Stroke-Associated Pneumonia; SHS, Stroke-heart Syndrome; SII, Systemic Immune-inflammation Index; sICH, Symptomatic Intracerebral Hemorrhage; IVT, Intravenous Thrombolysis; ICU, Intensive Care Unit; fDNI, Failure of Delayed Neurological Improvement.

R, Retrospective; P, Prospective; S, Single-center; M, Multi-center. M, Male; F, Female; h, hours; d, day; y, year; mon, month; mos, months; w, week.

① Hypertension; ② Diabetes; ③ Dyslipidaemia; ④ Heart Diseases (Coronary Heart Disease/Heart Failure/Myocardial Infarction, etc.); ⑤ Atrial Fibrillation; ⑥ Previous Cerebrovascular Diseases (Stroke/TIA, etc.); ⑦ Kidney Diseases; ⑧ Respiratory Diseases (Asthma/Chronic Obstructive Pulmonary Disease, etc.); ⑨ Infections; ⑩ Cancer; ⑪ Dementia; ⑫ Peripheral Arterial Diseases; ⑬ Carotid Diseases (Carotid Plaque/Carotid Atherosclerosis/Carotid Artery Stenosis, etc.); ⑭ Other Diseases; ⑮ Antiplatelets; ⑯ Anticoagulants; ⑰ Antithrombotics; ⑱ Statins; ⑲ IVT; ⑳ EVT; ㉑ Antibiotics; ㉒ Antihypertensive Drugs; ㉓ Hypoglycemic Drugs; ㉔ Other Drugs or Therapies.

A, Poor Prognosis; B, Mortality; C, Admission NIHSS; D, HT/sICH; E, END; F, SAP/PSP; G, AIS Severity; H, PSD; I, Stroke Progression/Recurrence; J, Others.

**Table 2:** Quality assessment based on the Newcastle–Ottawa Scale (NOS).

Cohort studies										
No.	Study	Total	Selection				Comparability	Outcome		
			1	2	3	4	1	1	2	3
			Representativeness of the exposed cohort	Selection of the non-exposed cohort	Ascertainment of exposure	Demonstration that outcome of interest was not present at start of study	Comparability of cohorts on the basis of the design or analysis	Assessment of outcome	Was follow-up long enough for outcomes to occur	Adequacy of follow-up of cohorts
1	Yang Y 2024 (5)	8	☆	☆	☆	☆	☆☆	×	☆	☆
2	Huang SW 2024 (1)	8	☆	☆	☆	☆	☆☆	☆	☆	×
3	Arslan 2024 (6)	7	☆	☆	☆	☆	☆	☆	×	☆
4	Zhu 2024 (7)	7	☆	☆	☆	☆	☆☆	×	×	☆
5	Cao 2024 (8)	8	☆	☆	☆	☆	☆☆	×	☆	☆
6	Cheng 2024 (9)	8	☆	☆	☆	☆	☆☆	×	☆	☆
7	Misirlioglu 2024 (10)	6	☆	☆	☆	☆	×	☆	×	☆
8	Hao 2024 (11)	7	☆	☆	☆	×	☆☆	☆	×	☆
9	Lee 2024 (12)	8	☆	☆	☆	☆	☆☆	☆	×	☆
10	Wang N 2024 (13)	9	☆	☆	☆	☆	☆☆	☆	☆	☆
11	Chen GJ 2024 (14)	8	☆	×	☆	☆	☆☆	☆	☆	☆
12	Zhang LL 2024 (15)	9	☆	☆	☆	☆	☆☆	☆	☆	☆
13	Wei 2024 (16)	9	☆	☆	☆	☆	☆☆	☆	☆	☆
14	Zhao 2024 (17)	7	☆	☆	☆	☆	☆	☆	×	☆
15	Guoqing 2024 (18)	7	☆	☆	☆	☆	☆	☆	☆	×
16	Mengting 2024 (20)	6	☆	☆	☆	☆	☆	×	☆	×
17	Zhouquan 2024 (23)	6	☆	☆	☆	☆	☆	×	☆	×
18	Jiaxiang 2024 (25)	6	☆	☆	☆	☆	☆	×	☆	×
19	Ma L 2024 (21)	7	☆	☆	☆	☆	☆	×	☆	☆
20	Zhang J 2024 (27)	5	☆	☆	☆	☆	☆	×	×	×
21	Huang H 2024 (29)	7	☆	☆	☆	☆	☆	☆	×	☆
22	Haimei 2024 (30)	5	☆	☆	☆	☆	×	×	☆	×
23	Lijun 2024 (31)	5	☆	☆	☆	☆	×	×	☆	×
24	Zhang MK 2024 (32)	9	☆	☆	☆	☆	☆☆	☆	☆	☆
25	Lin 2023 (42)	7	☆	☆	☆	☆	☆	×	☆	☆
26	Xiao 2023 (43)	6	☆	☆	☆	☆	☆☆	×	×	×
27	Dan-dan 2023 (44)	6	☆	☆	☆	☆	×	☆	☆	×
28	Zhou 2023 (46)	7	☆	☆	☆	☆	☆	☆	☆	×
29	Shao 2023 (47)	6	☆	☆	☆	×	☆	☆	☆	×
30	Wang X 2023 (48)	6	☆	☆	☆	☆	×	×	☆	☆
31	Song 2023 (49)	6	☆	☆	☆	☆	☆	×	☆	×
32	Wang YL 2023 (50)	6	☆	☆	☆	☆	☆	×	☆	☆
33	Liu HT 2023 (51)	6	☆	☆	☆	☆	☆	c	☆	×
34	Wang ZT 2023 (52)	7	☆	☆	☆	☆	☆☆	×	☆	×
35	Dong 2023 (53)	6	☆	☆	☆	☆	☆	×	☆	×
36	Gao 2023 (54)	8	☆	☆	☆	☆	☆☆	×	☆	☆
37	Huixin 2023 (55)	6	☆	☆	☆	☆	×	×	☆	×
38	Liu YY 2023 (56)	6	☆	☆	☆	☆	×	☆	☆	☆
39	Su 2023 (57)	6	☆	☆	☆	☆	☆	×	☆	×
40	Fernández-Garza 2023 (35)	9	☆	☆	☆	☆	☆☆	☆	☆	☆
41	Wang S 2023 (36)	7	☆	☆	☆	☆	×	☆	☆	☆
42	Ma 2023 (37)	9	☆	☆	☆	☆	☆☆	☆	☆	☆
43	Zhao 2023 (38)	8	☆	☆	☆	☆	☆☆	×	☆	☆
44	Hu 2023 (39)	8	☆	☆	☆	☆	☆☆	☆	☆	×
45	Zhang 2023 (40)	8	☆	☆	☆	☆	☆	☆	☆	☆
46	Chu 2023 (41)	8	☆	☆	☆	☆	☆	☆	☆	☆
47	Li 2023 (45)	6	☆	☆	☆	☆	×	☆	☆	×
48	Ma 2022 (68)	6	☆	☆	☆	☆	×	☆	☆	×
49	Liu 2022 (69)	9	☆	☆	☆	☆	☆☆	☆	☆	☆
50	Laiyun Z 2022 (70)	6	☆	☆	☆	☆	☆	×	☆	×
51	Wenli Z 2022 (71)	7	☆	☆	☆	☆	☆	☆	☆	×
52	Li 2022 (58)	7	☆	☆	☆	☆	☆	☆	×	☆
53	Zhang 2022 (59)	9	☆	☆	☆	☆	☆☆	☆	☆	☆
54	Wang 2022 (60)	8	☆	☆	☆	☆	☆☆	×	☆	☆
55	Wu 2022 (61)	8	☆	☆	☆	☆	☆☆	×	☆	☆
56	Ji 2022 (62)	8	☆	☆	☆	☆	☆☆	×	☆	☆
57	Lin 2022 (63)	6	☆	☆	☆	☆	×	☆	☆	☆
58	Yang 2022 (64)	8	☆	☆	☆	☆	☆☆	☆	×	☆
59	Zhou 2022 (65)	6	☆	☆	☆	☆	☆	×	☆	☆
60	Chen 2022 (66)	5	☆	☆	☆	☆	×	☆	×	×
61	Adiguzel 2022 (67)	5	☆	☆	☆	☆	×	×	☆	×
62	Wei L 2021 (76)	6	☆	☆	☆	☆	☆☆	×	×	×
63	Cheng 2021 (77)	6	☆	☆	☆	☆	☆	×	☆	×
64	Zhong 2021 (79)	8	☆	☆	☆	☆	☆☆	×	☆	☆
65	Hu 2021 (72)	9	☆	☆	☆	☆	☆☆	☆	☆	☆
66	Yi 2021 (73)	7	☆	☆	☆	☆	☆	☆	☆	×
67	Li LH 2021 (74)	7	☆	☆	☆	☆	☆	☆	☆	×
68	Weng 2021 (75)	8	☆	☆	☆	☆	☆☆	☆	☆	×
69	Wei-shi 2021 (78)	8	☆	☆	☆	☆	☆☆	☆	☆	×
70	Ceng 2020 (80)	9	☆	☆	☆	☆	☆☆	☆	☆	☆
71	Zhao 2020 (81)	8	☆	☆	☆	☆	☆☆	☆	☆	×
72	Chu 2020 (82)	6	☆	☆	☆	☆	☆	☆	×	×
73	Wang SN 2024 (34)	4	☆	☆	☆	☆	×	×	×	×

Case–control studies										
No.	Study	Total	Selection				Comparability	Exposure		
			1	2	3	4	1	1	2	3
			Adequate case-definition	Representativeness of the cases	Selection of controls	Definition of controls	Comparability of cases and controls on the basis of the design or analysis	Ascertainment of exposure	Same Method of ascertainment for cases and controls	Non-response rate
1	Zheng 2024 (33)	6	☆	☆	☆	☆	×	☆	×	☆
2	Zhou 2024 (19)	6	☆	☆	☆	☆	☆	×	☆	×
3	Tianlu 2024 (24)	6	☆	☆	☆	☆	☆	×	☆	×
4	Yu 2024 (77)	6	☆	☆	☆	☆	☆	×	×	☆
5	Niu 2024 (26)	5	☆	☆	☆	☆	☆	×	×	×
6	Dong 2024 (28)	8	☆	☆	☆	☆	☆☆	☆	×	☆

*Wang SN 2024 (34) was excluded from the meta-analysis because of low quality (Nos ≤ 4). The meanings of the ☆, ☆☆, and × can be found at the official instruction website of the NOS scale: https://www.ohri.ca/programs/clinical_epidemiology/oxford.asp.

### 3.2 Study characteristics

This systematic review and meta-analysis encompassed 40,682 individuals; the sample size ranged from a minimum of 22 (56) to a maximum of 9,107 (60). Not all studies reported the sex distribution and age data, preventing the accurate calculation of these data. Geographically, 68 studies were conducted in China (1, 5, 7–9, 11, 13–33, 36–38, 40–60, 62–65, 68–72, 75–81), 11 studies were conducted in other states or area including Turkey (*n* = 3) (6, 10, 67), China Taiwan (*n* = 3) (66, 74, 82), America (*n* = 2) (61), Korea (*n* = 2) (12, 73), Mexico (*n* = 1) (35). Moreover, studies (1, 5–8, 10, 13, 15–33, 35–41, 43, 44, 46–51, 53–59, 61–71, 73–75, 77–79, 81, 82) were retrospective, and 11 studies (9, 11, 12, 14, 42, 45, 52, 60, 72, 76, 80) were prospective. At the same time, 73 studies (5–13, 15–33, 35–59, 61, 63–72, 74–82) were single-center, and 5 studies (1, 14, 60, 62, 73) were multi-center. The number of studies reporting data on outcomes were as follows: poor prognosis (*n* = 43) (1, 6–8, 12–14, 16–18, 20, 21, 23, 33, 35–38, 41, 42, 44–46, 48, 50–52, 55, 56, 60, 62, 65–71, 73, 75, 79–82), mortality (*n* = 12) (1, 5, 6, 10, 16, 37, 39, 60, 61, 66, 67, 80), severity (*n* = 9) (7, 21, 31, 35, 42, 69, 70, 75, 79), HT/sICH (*n* = 12) (8, 13, 16, 22, 26, 33, 44, 51, 54, 64, 69, 73), END (*n* = 8) (12, 13, 16, 21, 25, 29, 38, 52), SAP/PSP (*n* = 10) (19, 24, 30, 33, 40, 67, 76, 77, 79, 80), PSD (*n* = 3) (28, 49, 72), progression/recurrence (*n* = 4) (12, 37, 60, 78), admission NIHSS (*n* = 11) (5, 12–14, 16, 46, 59, 64, 72, 73, 75), and other complications (*n* = 25) (1, 9, 11, 15, 27, 32, 33, 42, 43, 47, 49, 51, 53, 57–59, 62, 63, 67, 71–74, 78, 82).

### 3.3 Predictive value of SII for AIS poor prognosis (primary outcome)

#### 3.3.1 Predictive value of continuous SII for AIS poor prognosis

**Figure 2:**
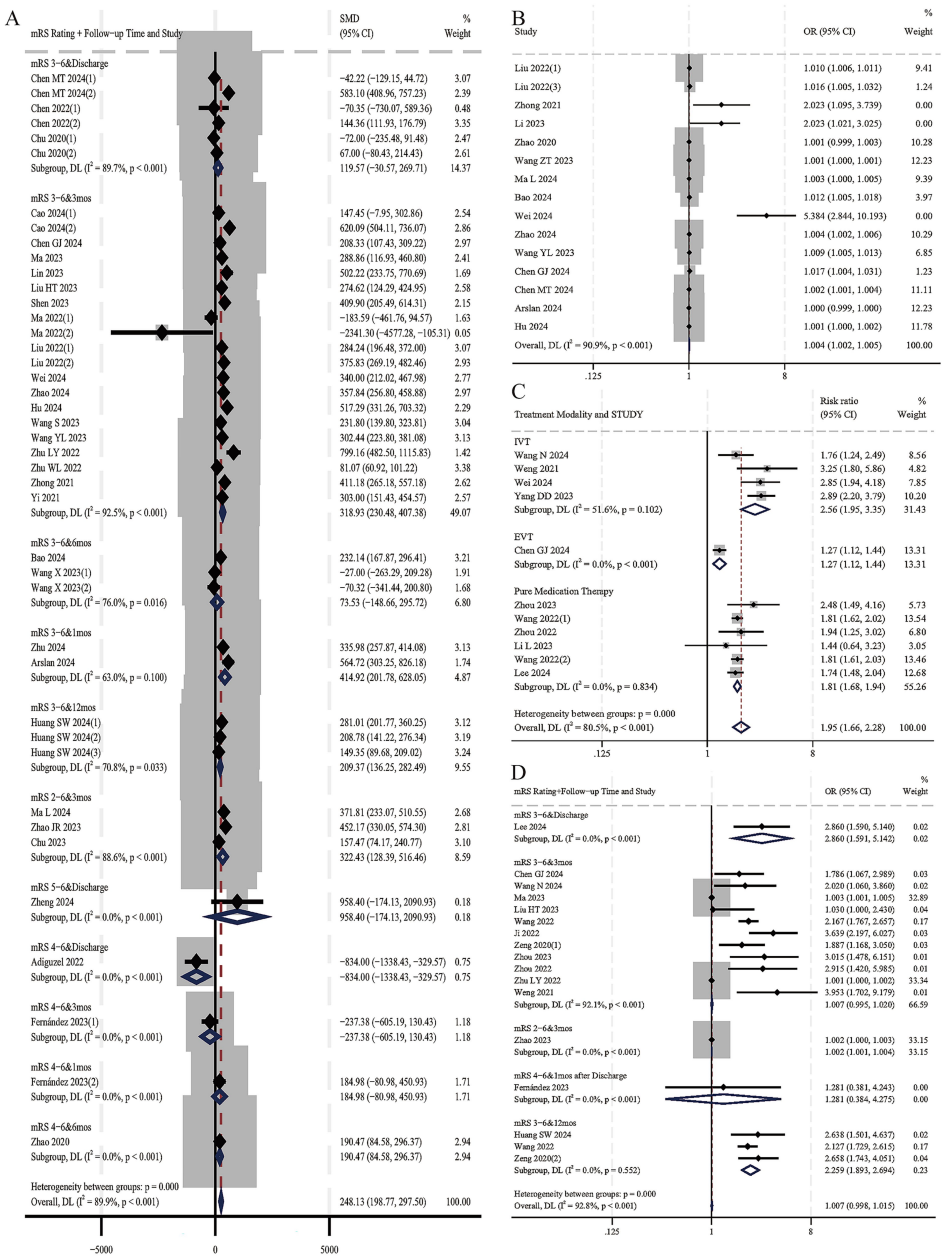
Forest plots of associations between AIS poor prognosis and SII. **(A)** Continuous SII value in poor prognosis groups vs. favorable prognosis groups; **(B)** Pooled OR of continuous SII in predicting poor prognosis; **(C)** The sample size of poor prognosis patients in high SII groups vs. low SII groups; **(D)** Pooled OR of high SII in predicting poor prognosis.

**Figure 3:**
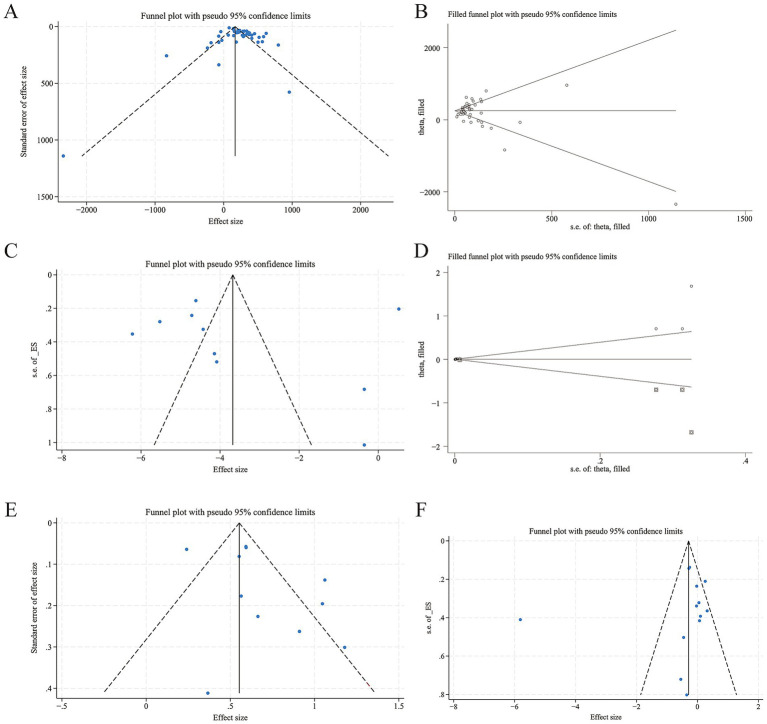
Funnel plots and trim-and-fill plots of associations between AIS poor prognosis and SII. **(A)** Funnel plot-continuous SII value in poor prognosis groups vs. favorable prognosis groups; **(B)** Trim-and-fill plot-continuous SII value in poor prognosis groups vs. favorable prognosis groups; **(C)** Funnel plot-pooled OR of continuous SII in predicting poor prognosis; **(D)** Trim-and-fill plot-pooled OR of continuous SII in predicting poor prognosis; **(E)** Funnel plot-the sample size of poor prognosis patients in high SII groups vs. low SII groups; **(F)** Funnel plot-pooled OR of high SII in predicting poor prognosis.

A total of 32 studies (1, 6–8, 14, 16–18, 20, 21, 23, 33, 35–38, 41, 42, 48, 50, 51, 55, 66–71, 73, 79, 81, 82), which included 42 designs and involved 14,915 AIS patients, were included. Among them, 6,198 patients were in the poor prognosis groups, and 8,717 were in the favorable prognosis groups. A total of 16 studies (8, 14, 16, 17, 23, 36, 37, 42, 50, 55, 68–71, 73, 79) with 20 designs adopted the guideline-recommended 3-month modified Rankin Scale (mRS) 3–6 as the poor prognostic criterion (83). Ultimately, 26 studies came from China (1, 7, 8, 14, 16–18, 20, 21, 23, 33, 36–38, 41, 42, 48, 50, 51, 55, 68–71, 79, 81), and 6 from other countries and regions (6, 35, 66, 67, 73, 82). In the meantime, 12 studies mentioned IVT (16–18, 20, 21, 23, 36, 38, 41, 50, 51, 68), 6 mentioned EVT (8, 14, 48, 55, 71, 73), and 14 used pure medication therapy (1, 6, 7, 33, 35, 37, 42, 66, 67, 69, 70, 79, 81, 82). *I^2^* = 89.9% > 50%, *Q* statistics *p* = 0.000, indicating a high level of heterogeneity among 42 designs. Meta-regression was conducted with effect size (ES) as the dependent variable and the 5 possible sources of heterogeneity (mRS rating, follow-up time, treatment modality, regional distribution, and mRS rating + follow-up time) as independent variables. The meta-regression results showed that for the 5 independent variables, all *p*-values were > 0.05 (0.444; 0.380; 0.275; 0.745; 0.643), indicating that the heterogeneity was not related to these 5 factors, and the source of heterogeneity needs to be further explored. Random-effects model showed the baseline SII value was significantly higher in poor prognosis groups (SMD = 248.13, 95% CI: 198.77 to 297.50, *p* = 0.000, Figure 2A), meaning that the SII value of the poor prognosis groups was 248.13 × 10^9^/L higher than that of the favourable prognosis groups significantly. Figure 3A shows the funnel plot was asymmetric, Begg *p* = 0.319 > 0.05, Egger *p* = 0.004 < 0.05, indicating a slight publication bias in the 42 designs. After applying the trim-and-fill method, the significance of the overall effect size and the heterogeneity did not change, suggesting that publication bias did not distort the conclusions of this meta-analysis (Figure 3B).

A total of fourteen studies (6, 14, 16–18, 20, 21, 23, 45, 50, 52, 69, 79, 81), with 15 designs, evaluated the aORs of continuous SII in predicting AIS poor prognosis. High heterogeneity was found (*I^2^* = 90.9%, *Q*-statistic, *p* = 0.000). Meta-regression indicated that neither follow-up time nor treatment modality was a source of heterogeneity (*p* = 0.578; 0.489). Figure 2B shows a trend: with an increase in continuous SII, the incidence of poor prognosis may be slightly higher (OR = 1.004, 95% CI: 1.002 to 1.005, *p* = 0.000). The funnel plot in Figure 3C shows a specific publication bias in the 15 designs (Begg *p* = 0.020, Egger *p* = 0.834). The trim-and-fill analysis showed that the number of imputed missing studies was negligible, and the adjusted effect size (OR = 1.003, 95% CI: 1.002 to 1.005, *p* = 0.000) was almost consistent with the unadjusted one (Figure 3D).

Additionally, 2 studies involved aORs of SII per 1 standard deviation (SD) to predict AIS poor prognosis. Chen GJ 2024 (14) reported aOR = 1.241 (95% CI: 1.051 to 1.465), and Huang SW 2024 (1) reported aOR = 1.191 (95% CI: 1.006 to 1.410), indicating that for every 1 SD increase in SII, the likelihood of a poor prognosis increases in AIS patients.

#### 3.3.2 Predictive value of categorized SII for AIS poor prognosis

A total of 10 studies (12–14, 16, 44–46, 60, 65, 75), with 11 designs, provided data on the sample size of poor/favorable prognosis patients in both high and low SII groups; all criteria of poor prognosis were mRS 3–6. Among 21,719 patients, 5,761 were in high SII groups, and 15,958 were in low SII groups. High heterogeneity was noted (*I^2^* = 92.9%, *Q* statistics *p* = 0.000), and meta-regression showed follow-up time, regional distribution, and treatment modality were not sources of heterogeneity (*p* = 0.590; 0.459; 0.593). Subgroup analysis by treatment modality in Figure 2C revealed less within-group heterogeneity. A random-effects model for all designs indicated RR = 1.95 (95% CI: 1.66 to 2.28, *p* = 0.000), meaning patients with High SII were 1.95 times more likely to have a poor prognosis significantly. The almost symmetrical funnel plot (Begg *p* = 0.876, Egger *p* = 0.134) suggests that there is no expected publication bias, as shown in Figure 3E.

A total of 16 studies (1, 8, 12, 14, 32, 35, 37, 38, 46, 51, 60, 62, 65, 70, 75, 80) with 19 designs reported aORs of categorized SII in predicting poor prognosis. Among them, CAO 2024 (8) with 2 designs was removed as its aOR = 1.000 (95% CI: 1.000 to 1.000) made log-conversion in STATA difficult. The remaining studies had substantial heterogeneity (*I^2^* = 92.8%, *Q*-statistic *p* = 0.000). Meta-regression showed that four variables (follow-up time, regional distribution, treatment modality, and mRS rating+follow-up time) were not the source of heterogeneity (*p* = 0.866; 0.893; 0.710; 0.949 > 0.05). Figure 2D shows that the random-effects model pooled OR = 1.007 (95% CI: 0.998 to 1.015, *p* = 0.120), indicating a higher but non-significantly poor prognosis risk in the high SII groups compared to the low SII groups. Funnel plots for the 17 designs were symmetrical, and bias tests (Begg *p* = 0.760, Egger *p* = 0.833) suggested likely no publication bias in the designs (Figure 3F).

### 3.4 Predictive value of SII for AIS secondary outcomes (mortality, severity, HT/sICH, END, PSD, progression/recurrence, and other complications)

#### 3.4.1 Continuous SII

**Figure 4:**
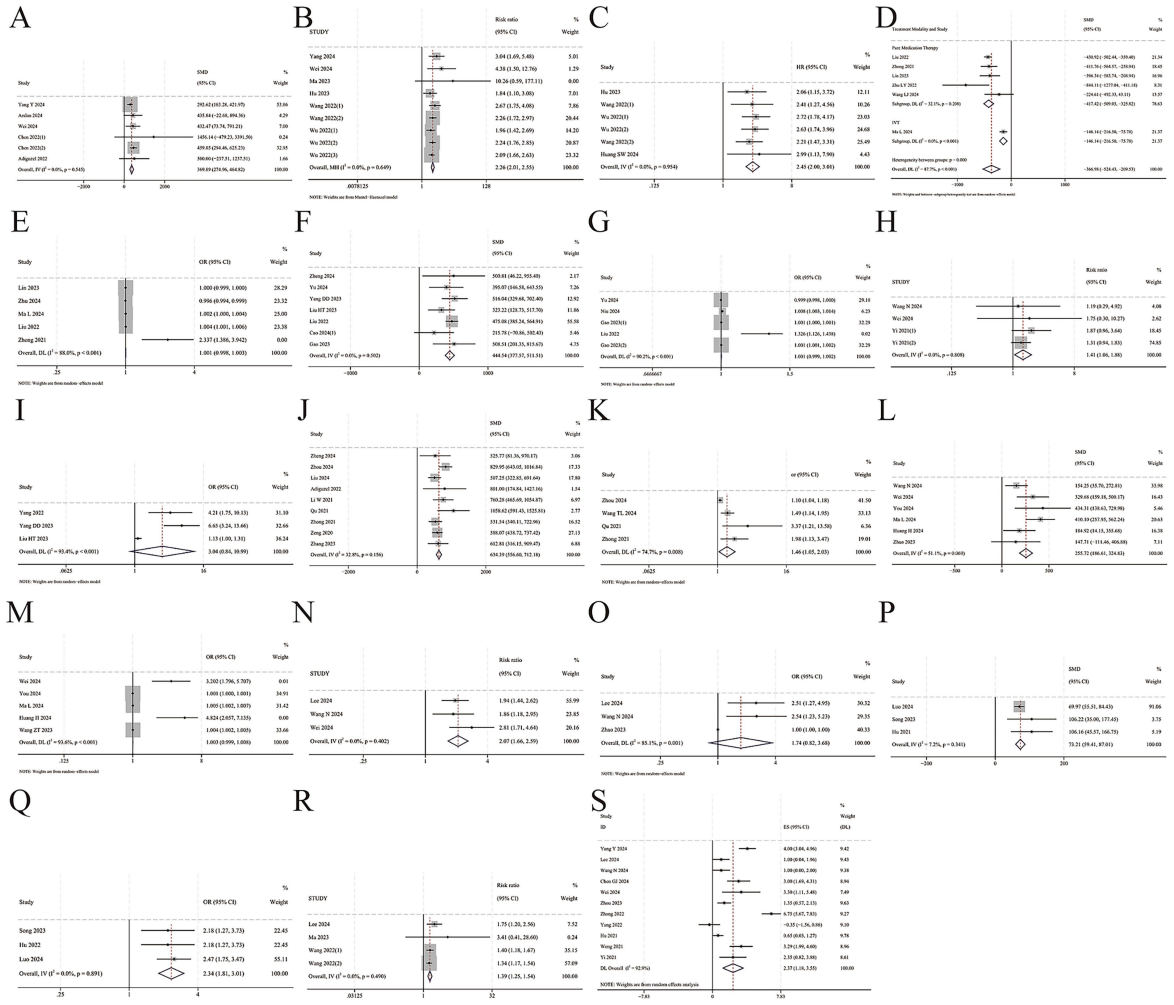
Forest plots of associations between AIS secondary outcomes and SII. **(A)** Continuous SII value in death groups vs. survival groups; **(B)** The sample size of death patients in high SII groups vs. low SII groups; **(C)** Pooled HR of high SII in predicting mortality; **(D)** Continuous SII value in mild severity groups vs. mild-moderate severity groups; **(E)** Pooled OR of continuous SII in predicting severity; **(F)** Continuous SII value in HT/sICH groups vs. non-HT/sICH groups; **(G)** Pooled OR of continuous SII in predicting HT/sICH; **(H)** The sample size of HT/sICH patients in high SII groups vs. low SII groups; **(I)** Pooled OR of high SII in predicting HT/sICH; **(J)** Continuous SII value in SAP/PSP groups vs. non-SAP/PSP groups; **(K)** Pooled OR of continuous SII in predicting SAP/PSP; **(L)** Continuous SII value in END groups vs. non-END groups; **(M)** Pooled OR of continuous SII in predicting END; **(N)** The sample size of END patients in high SII groups vs. low SII groups; **(O)** Pooled OR of high SII in predicting END; **(P)** Continuous SII value in PSD groups vs. survival groups; **(Q)** Pooled OR of high SII in predicting PSD; **(R)** The sample size of progression/recurrence patients in high SII groups vs. low SII groups; **(S)** Admission NIHSS in high SII groups vs. low SII groups.

**Figure 5:**
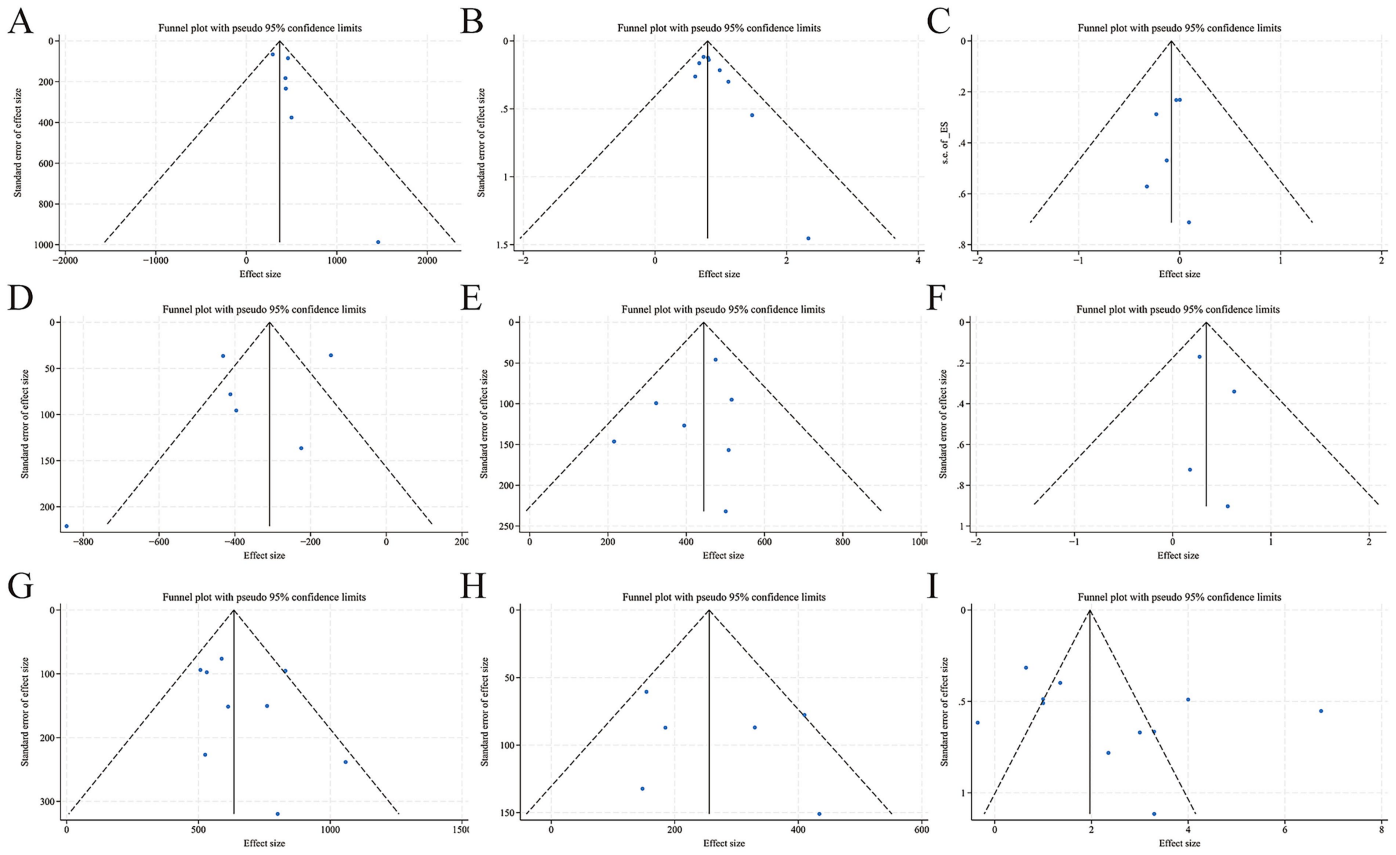
Funnel plots of associations between AIS poor prognosis and SII. **(A)** Continuous SII value in death groups vs. survival groups; **(B)** The sample size of death patients in high SII groups vs. low SII groups; **(C)** Pooled HR of high SII in predicting mortality; **(D)** Continuous SII value in mild severity groups vs. mild-moderate severity groups; **(E)** Continuous SII value in HT/sICH groups vs. non-HT/sICH groups; **(F)** The sample size of HT/sICH patients in high SII groups vs. low SII groups; **(G)** Continuous SII value in SAP/PSP groups vs. non-SAP/PSP groups; **(H)** Continuous SII value in END groups vs. non-END groups; **(I)** Admission NIHSS in high SII groups vs. low SII groups.

1. Continuous SII values were listed in both the death, mild severity, HT/sICH, SAP/PSP, END, PSD, Progression/Recurrence groups, and the corresponding control groups, including 5 (5, 6, 16, 66, 67), 6 (21, 31, 42, 69, 70, 79), 8 (8, 22, 26, 33, 44, 51, 54, 69), 10 (19, 24, 30, 33, 40, 67, 76, 77, 79, 80), 7 (13, 16, 21, 25, 29, 38, 52), and 3 (28, 49, 72), 1 (27) studies. The baseline SII value was significantly higher in the death groups, SMD = [369.889 (95% CI: 274.957 to 464.822), *p* = 0.000, *I^2^* = 0.0%, *Q* statistics *p* = 0.545, fixed, Figure 4A; Begg *p* = 0.707, Egger *p* = 0.150, Figure 5A]; mild severity groups SMD = [−366.98 (95% CI: −524.43 to −209.53), *p* = 0.000, *I^2^* = 87.7%, *Q* statistics *p* = 0.000, random, Figure 4D; Begg *p* = 1.000, Egger *p* = 0.166, Figure 5D]; HT/sICH groups [Excluding NIU 2024 (26), one design of Gao 2023 (54) and one design of Cao 2024 (8), SMD = 444.540 (95% CI: 377.566 to 511.514), *p* = 0.000, *I^2^* = 0.0%, *Q* statistics *p* = 0.502, fixed, Figure 4F; Begg *p* = 0.371, Egger *p* = 0.274, Figure 5E]; SAP/PSP (Excluding Tianlu 2024 (24), SMD = 634.39 (95% CI: 556.60 to 712.18), *p* = 0.000, *I^2^* = 32.8%, *Q* statistics *p* = 0.156, fixed, Figure 4J; Begg *p* = 0.34, Egger *p* = 0.311, Figure 5G]; END (Excluding Wang ZT 2023 (52), SMD = 255.72 (95% CI: 186.61 to 324.83), *p* = 0.000, *I^2^* = 51.1%, *Q* statistics *p* = 0.069, fixed, Figure 4L; Begg *p* = 0.707, Egger *p* = 0.536, Figure 5H]; PSD SMD = [73.21(95% CI: 59.41 to 87.01), *p* = 0.000, *I^2^* = 7.2%, *Q* statistics *p* = 0.341, fixed, Figure 4P]; Progression/Recurrence groups [Progression/Recurrence groups SII = 557.00 (345.00, 832.88); Non-Progression/Recurrence groups SII = 420.63 (310.58, 546.48), *p* = 0.011].
2. Adjusted ORs of continuous SII in predicting AIS mortality, mild severity, HT/sICH, SAP/PSP, and END were reported in 2 (6, 16), 5 (7, 21, 42, 69, 79), 4 (22, 26, 54, 69), 4 (19, 24, 77, 79), and 5 (16, 21, 25, 29, 52) studies. Except for severity, the incidence of adverse outcomes could be higher with an increase significantly in continuous SII, Mortality pooled OR = [2.592 (95% CI: 1.046 to 6.421), *p* = 0.040]; severity pooled OR = [1.001(95% CI: 0.998 to 1.003), *p* = 0.718, *I^2^* = 88.0%, *Q* statistics *p* = 0.000, random, Figure 4E]; HT/sICH pooled OR = [1.001 (95% CI: 0.999 to 1.002), *p* = 0.000, *I^2^* = 90.2%, *Q* statistics *p* = 0.000, random, Figure 4G]; SAP/PSP pooled OR = [1.46 (95% CI: 1.05 to 2.03), *p* = 0.000, *I^2^* = 74.7%, *Q* statistics *p* = 0.008, random, Figure 4K]; END pooled OR = [1.003 (95% CI: 0.999 to 1.008), *p* = 0.123, *I^2^* = 93.6%, Q statistics *p* = 0.000, random, Figure 4M].
3. Huang SW 2024 (1) mentioned SII per 1 SD to predict mortality, aHR = 1.195 (95% CI: 1.072 to 1.332), *p* = 0.001. Yang 2022 (64) mentioned SII per 10 SD to predict HT/sICH, aOR = 1.005 (95% CI: 1.002 to 1.008), *p* = 0.002.

#### 3.4.2 Categorized SII

1. The sample size of death, HT/sICH, END, progression/recurrence patients in both High SII vs. Low SII groups was listed, including 6 (5, 16, 37, 39, 60, 61), 3 (13, 16, 73), 3 (12, 13, 16), 3 (12, 37, 60) studies. The sample size of adverse outcomes patients of high SII groups were significantly higher than low SII groups, death pooled RR = [2.26 (95% CI: 2.01 to 2.55, *p* = 0.000, *I^2^* = 0%, *Q* statistics *p* = 0.649, fixed, Figure 4B; Begg *p* = 0.076, Egger *p* = 0.036, Figure 5B]; HT/sICH pooled RR = [1.41 (95% CI: 1.06 to 1.88), *p* = 0.019, *I^2^* = 0.0%, *Q* statistics *p* = 0.808, fixed, Figure 4H; Begg *p* = 0.734, Egger *p* = 0.601, Figure 5F]; END pooled RR = [2.07 (95% CI: 1.66 to 2.59), *p* = 0.000, *I^2^* = 0.0%, *Q* statistics *p* = 0.402, fixed, Figure 4N]; Progression/Recurrence pooled RR = [1.39 (95% CI: 1.25 to 1.54), *p* = 0.000, *I^2^* = 0.0%, *Q* statistics *p* = 0.490, fixed, Figure 4R].
2. Adjusted ORs of categorized SII in predicting AIS mortality, severity, HT/sICH, SAP/PSP, END, PSD, Progression/Recurrence were reported in 2 (5, 80), 1 (35), 4 (8, 44, 51, 64), 2 (76, 80), 3 (12, 13, 38), 3 (28, 49, 72), and 1 (27) studies. Except for END, the risk of adverse outcomes in high SII groups was significantly higher than in low SII groups, mortality pooled OR = [Ceng 2020 (80) ① 90d: 7.332 (95% CI: 1.608 to 33.419, *p* = 0.01; ② 1y: 5.15 (95% CI: 1.918 to 13.841), *p* = 0.001; Yang Y 2024 (5) 4.671(95% CI: 1.379 to 15.826), *p* = 0.013]; severity pooled OR = [7.462 (95% CI: 1.666 to 33.333), *p* = 0.009]; HT/sICH pooled OR = [Excluding CAO 2024 (8), 3.04 (95% CI: 0.84 to 8.99), *p* = 0.000, *I^2^* = 93.4%, *Q* statistics *p* = 0.000, random, Figure 4I]; SAP/PSP pooled OR = [Ceng 2020 (80) 6.803 (95% CI: 3.251 to 14.236), *p* = 0.000; Wei 2021 (76) 0.999 (95% CI: 0.998 to 1.000), *p* = 0.060], END pooled OR = [1.74(95% CI: 0.82 to 3.68), *p* = 0.150, *I^2^* = 85.1%, *Q* statistics *p* = 0.001, random, Figure 4O]; PSD pooled OR = [2.34 (95% CI: 1.81 to 3.07), *p* = 0.000, *I^2^* = 0.0%, *Q* statistics *p* = 0.891, fixed, Figure 4Q]; Progression/Recurrence pooled OR = [1.003(95% CI: 1.000485 to 1.005), *p* = 0.017].
3. Adjusted HRs of categorized SII in predicting AIS mortality were reported in 4 studies (1, 39, 60, 61), pooled HR = 2.45 (95% CI: 2.00 to 3.01, *p* = 0.000, *I^2^* = 0.0%, *Q* statistics *p* = 0.954, fixed, Figure 4C; Begg *p* = 0.707, Egger *p* = 0.589, Figure 5C).
4. A total of 11 studies (5, 12–14, 16, 46, 59, 64, 72, 73, 75) provided data on the value of Admission NIHSS in both high SII and low SII groups, NIHSS in high SII groups were significantly higher, pooled SMD = 2.365 (95% CI: 1.178 to 3.552, *p* = 0.003, *I^2^* = 92.94%, *Q* statistics *p* = 0.000, random, Figure 4S; Begg *p* = 0.350, Egger *p* = 0.242, Figure 5I).

### 3.5 Other complications

A total of 17 studies (1, 9, 11, 15, 32, 42, 43, 47, 51–53, 57–59, 62, 71, 78) listed continuous/categorized SII aORs/aHRs in other complication groups studied, as shown in Table 3. A total of 13 studies (9–11, 15, 33, 43, 47, 51, 57, 62, 63, 70, 78) listed continuous SII values in other complications groups were studied, as shown in Table 4.

**Table 3:** Continuous/categorized SII aORs/aHRs in other complications.

No.	Study	Indicators (continuous SII/HIGH SII)	Outcomes	aOR/aHR(95% CI)
1	Huang SW 2024 (1) (1)	High SII vs. Low SII	Functional Dependency	2.894 (1.093, 7.659)
2	Huang SW 2024 (1) (2)	High SII vs. Low SII	Stroke-associated Infection	2.655 (1.490, 4.731)
3	Cheng 2024 (9)	High SII vs. Low SII	Post-stroke Cognitive Impairment	10.369 (4.460, 24.107)
4	Liu HT 2023 (51)	High SII vs. Low SII	Atrial fibrillation Stroke	1.116 (1.024, 1.438)
5	Zhang 2022 (59) (1)	High SII vs. Low SII	Vulnerable Plaques Presence	2.242 (1.378, 4.024)
6	Zhang 2022 (59) (2)	High SII vs. Low SII	Ruptured Fibrous Caps	3.462 (2.031, 6.074)
7	Li 2022 (58)	High SII vs. Low SII	Decompressive craniectomy	3.579 (1.360, 9.422)
8	Zhang LL 2024 (15) (1)	Continuous SII	Presence of Vulnerability Plaques	5.013 (2.671, 8.472)
9	Zhang LL 2024 (15) (2)	Continuous SII	Presence of Ulcerative Plaques	5.017 (3.010, 8.023)
10	Hao 2024 (11)	Continuous SII	Stroke-heart Syndrome	5.089 (1.981, 15.74)
11	Dong 2023 (53)	Continuous SII	First Pass Effect	0.895 (0.801, 0.971)
12	Wang ZT 2023 (52)	Continuous SII	Early Neurological Improvement	0.998 (0.997, 0.999)
13	Shao 2023 (47)	Continuous SII	Basal Ganglia-Enlarged Perivascular Spaces Severity	1.004 (1.001, 1.008)
14	Lin 2023 (42)	Continuous SII	Good Prognosis (90d/mRS 0–2)	1.000 (0.999, 1.001)
15	Xiao 2023 (43)	Continuous SII	PatentForamenOvale	0.99 (0.98, 1.01)
16	Su 2023 (57)	Continuous SII	Vascular Dementia	1.006 (1.002, 1.010)
17	Ji 2022 (62)	Continuous SII	Malignant Cerebral Edema	1.209 (1.034, 1.413)
18	Wenli Z 2022 (71)	Continuous SII	Ineffective Recanalization	3.731 (1.641, 10.602)
19	Huang SW 2024 (1) (3)	SII (per 1 SD)	Functional Dependency	1.224 (1.040, 1.441)
20	Huang SW 2024 (1) (4)	SII (per 1 SD)	Stroke-associated Infection	1.349 (1.139, 1.598)
21	Zhang MK 2024 (32)	SII (per 200 Units)	Failure of Delayed Neurological Improvement	1.065 (1.001, 1.132)
22	Wei 2021 (76)	continuous SII	Favorable Prognosis (Non-cerebrovascular Diseases Recurrence/2y)	1.284 (1.105, 1.493)

Only the effect size of the Wei2021 literature is aHR, and the rest are aORs. As shown in columns 3 and 4 of Table 3, four aOR/aHR (95% CI) data are mentioned in the literature by Huang SW (1). (1) represents the aOR/aHR (95% CI) with “High SII vs. Low SII” as the Indicator and “Functional Dependency” as the Outcome. (2) represents the aOR/aHR (95% CI) with “High SII vs. Low SII” as the Indicator and “Stroke-associated Infection” as the Outcome. (3) represents the aOR/aHR (95% CI) with “SII (per 1 SD)” as the Indicator and “Functional Dependency” as the Outcome. (4) represents the aOR/aHR (95% CI) with “SII (per 1 SD)” as the Indicator and “Stroke-associated Infection” as the Outcome.

**Table 4:** Continuous SII values in other complication groups and the corresponding control groups.

No.	Complications	Study	Group 1		Group 2		Group 3	
			*n*	SII [M (Q1, Q3]/ $\bar{X}$ ± s	*n*	SII [M (Q1, Q3]/ $\bar{X}$ ± s	*n*	SII [M(Q1, Q3]/ $\bar{X}$ ± s
1&2	AF-S/Non-AF-S	Liu HT 2023 (51)	144	759 (516, 1,549)	370	480 (379, 1,081)	–	–
		Lin 2022 (63)	173	802.29 (473.08, 1390.30)	353	562.50 (379.73, 1040.33)	–	–
3	Plaque: Vulnerable/Stable	Zhang LL 2024 (15)	144	684.6 (553.2, 819.7)	58	407.1 (293.4, 601.9)	–	–
4	Cerebral Herniation /Non-Cerebral Herniation	Zheng 2024 (33)	7	2184.13 (1849.47, 4724.67)	75	1336.41 (833.34, 2242.55)	–	–
5	SHS/Non-SHS	Hao 2024 (11)	24	1,100 (700, 1,500)	97	500 (400, 800)	–	–
6	PFO /Non-PFO	Xiao 2023 (43)	50	613.08 ± 202.03	50	411.64 ± 157.81	–	–
7	Vascular Dementia /Non-Vascular Dementia	Su 2023 (57)	56	579.35 ± 122.32	216	503.46 ± 122.41	–	–
8	MCE/Non-MCE	Ji 2022 (62)	132	2,460 ± 1,860	543	1,570 ± 1,300	–	–
9	PSCI/Non-PSCI	Cheng 2024 (9)	193	587.75 (337.42, 988.95)	139	345.66 (248.44, 572.89)	–	–
10	BG-EPVS Severity: Mild /Moderate–Severe	Shao 2023 (47)	57	466.16 (336.69, 603.12)	115	652.63 (463.75, 903.16)	–	–
11	CSO-EPVS Severity: Mild/Moderate–Severe	Shao 2023 (47)	100	579.45 (418.36, 775.58)	72	581.75 (391.48, 751.26)	–	–
11	Aetiology: Small Vessels/Large Vessels /Other Etiologies	Misirlioglu 2024 (10)	794	871.04 (650.62, 1102.69)	396	898.17 (565.27, 1165.79)	160	243.34 (142.97, 367.66)
12	Infarct Focus Volume: Small/Medium/Large	Laiyun Z 2022 (70)	77	565.13 (369.81, 741.89)	75	696.25 (441.22, 1072.71)	30	1187.28 (730.05, 2251.80)
13	Recurrent Cerebrovascular Disease (Ischemic Stroke/Hemorrhagic Stroke/Transient Ischemic Attack)	Wei 2021 (76)	24	1190.65 (439.77, 2290.33)	92	426.35 (311.45, 769.23)	–	–

AF-S, Atrial Fibrillation Stroke; SHS, Stroke-heart Syndrome; PFO, patent foramen ovale; MCE, Malignant Cerebral Edema; PSCI, Post-stroke Cognitive Impairment; BG-EPVS, Basal Ganglia-Enlarged Perivascular Spaces; CSO-EPVS, Central Semi-ovale Region Enlarged Perivascular Spaces.

### 3.6 SII cut-off values and AUC of ROC curves

A total of 51 studies (5, 6, 8, 9, 11–13, 15–21, 24–29, 32, 33, 35–39, 43, 44, 46, 47, 49–52, 54, 56, 58, 62, 65, 66, 68–70, 72, 73, 76, 77, 79, 80, 82) listed cut-off values, AUC (95% CI), sensitivity, and specificity of ROC curves, as shown in Table 5.

**Table 5:** SII cut-off values and AUC of ROC curves.

No.	Study	Outcomes	AUC (95% CI)	SII Cut-off	Sensitivity (%)	Specificity (%)
1. Poor prognosis						
1	Zheng 2024 (33)	Poor Prognosis (Discharge)	0.721 (0.561, 0.881)	1,192	92.3	44.9
2	Mengting 2024 (20)	Poor Prognosis (Discharge)	0.821 (0.746, 0.896)	753.68	87.2	74.8
3	Chu 2020 (82)	Poor Prognosis (Discharge)	NR	651	NR	NR
4(1)	Ma 2022(1) (68)	Poor Prognosis (3mos)	0.714 (0.514, 0.914)	974	75.0	85.7
4(2)	Ma 2022(2) (68)	Poor Prognosis (3mos)	0.688 (0.504, 0.871)	695	100	62.5
5(1)	Ceng 2020(1) (80)	Poor Prognosis (3mos)	0.612 (NR, NR)	555	68	49.9
6	Zhao 2024 (17)	Poor Prognosis (3mos)	0.779 (0.715, 0.843)	NR	NR	NR
7	Cao 2024 (8)	Poor Prognosis (3mos)	0.633 (0.583, 0.683)	1617.42	60.6	64.1
8	Liu YY 2023 (56)	Poor Prognosis (3mos)	0.848 (0.634, 1.000)	1103.22	NR	NR
9	Wang ZT 2023 (52)	Poor Prognosis (3mos)	0.702 (0.642, 0.762)	848.7	62.5	72.3
10	Liu HT 2023 (51)	Poor Prognosis (3mos)	0.701 (0.611, 0.790)	644	85.2	58.9
11	Liu 2022 (69)	Poor Prognosis (3mos)	0.880 (0.836, 0.924)	449.76	83.7	67.3
12	Ma 2023 (37)	Poor Prognosis (3mos)	0.715 (0.546, 0.826)	392.903	87.9	46.5
13	Wang YL 2023 (50)	Poor Prognosis (3mos)	0.880 (0.804, 0.957)	1012.06	90.8	79.2
14	Zhouquan 2024 (23)	Poor Prognosis (3mos)	0.715 (0.6550, 0.776)	868.55	55.7	84.0
15	Yi 2021 (73)	Poor Prognosis (3mos)	0.679 (0.643, 0.745)	853	NR	NR
16	Zhou 2023 (46)	Poor Prognosis (3mos)	0.657 (0.572, 0.742)	802.8	70.9	58.2
17	Zhou 2022 (65)	Poor Prognosis (3mos)	0.657 (0.572, 0.742)	802.8	70.9	58.2
18	Laiyun Z 2022 (70)	Poor Prognosis (3mos)	0.789 (0.712, 0.866)	781.4	74.5	74.0
19	Zhao 2023 (38)	Poor Prognosis (3mos)	0.787 (0.731, 0.843)	621.68	71.7	75.4
20	Wang S 2023 (36)	Poor Prognosis (3mos)	0.598 (0.552, 0.645)	582.755	65	53
21	Zhong 2021 (79)	Poor Prognosis (3mos)	0.702 (0.635, 0.769)	580	73.1	69.7
22	Wei 2024 (16)	Poor Prognosis (3mos)	0.717 (0.646, 0.788)	504.99	70.9	69.6
23	Ma L 2024 (21)	Poor Prognosis (3mos after Discharge)	0.826 (0.755, 0.898)	781.16	96.2	52.5
24	Arslan 2024 (6)	Poor Prognosis (28d)	0.645 (0.568, 0.722)	1,146	50.5	78.8
25	Fernández-Garza 2023 (35)	Poor Prognosis (30d)	0.634 (0.528, 0.741)	621.161	73.6	51.0
26	Guoqing 2024 (18)	Poor Prognosis (6mos)	0.841 (0.759, 0.924)	880.53	63.41	95.06
5(2)	Zeng 2020(2) (80)	Poor Prognosis (1y)	0.662 (NR, NR)	856.46	43.9	75.5
2. Mortality						
1(1)	Chen 2022(1) (66)	Mortality (Discharge)	NR	1,051	NR	NR
1(2)	Chen 2022(2) (66)	Mortality (Discharge)	0.707 (NR, NR)	2,120	50.0	91.4
2	Hu 2023 (39)	Mortality (Discharge)	0.65 (0.62, 0.68)	NR	NR	NR
3	Wei 2024 (16)	Mortality (3mos)	0.703 (0.582, 0.825)	524.47	78.9	59.9
4(1)	Ceng 2020(1) (80)	Mortality (3mos)	0.765 (NR, NR)	915.03	70.4	76.6
4(2)	Ceng 2020(2) (80)	Mortality (1y)	0.725 (NR, NR)	887.25	60.8	75.4
5	Yang Y 2024 (5)	Mortality (120d)	0.830 (0.710, 0.949)	666.31	72.7	92.0
3. HT/sICH						
1	Niu 2024 (26)	HT	0.604 (0.506, 0.701)	NR	27.60	43.20
2	Zheng 2024 (33)	HT	0.659 (0.541, 0.776)	1721.7914	58.3	71.7
3	Liu 2022 (69)	HT	0.857 (0.808, 0.907)	728.03	79.2	82.6
4	Dan-dan 2023 (44)	HT	0.784 (0.715, 0.853)	721	73.1	70.5
5	Liu HT 2023 (51)	HT	0.82 (0.747, 0.889)	706.3	83.7	53.2
6(1)	Gao 2023(1) (54)	HT	0.610 (0.535, 0.686)	488.48	69	47
6(2)	Gao 2023(2) (54)	sICH	0.739 (0.636, 0.842)	846.56	70	77
7	Cao 2024 (8)	sICH	0.707 (0.639, 0.776)	1817.83	70	65
8	Wei 2024 (16)	sICH	0.517 (0.279, 0.754)	NR	NR	NR
4. END						
1	Huang H 2024 (29)	END	0.798 (0.709, 0.888)	854.76	80.7	78.2
2	Jiaxiang 2024 (25)	END	0.658 (0.558, 0.758)	768.206	63.4	69.4
3	Wang N 2024 (13)	END	0.61 (0.54, 0.69)	591.63	58.1	64.6
4	Lee 2024 (12)	END	0.702 (0.620, 0.784)	588.9	NR	NR
5	Wei 2024 (16)	END	0.708 (0.631, 0.785)	504.99	70.7	62.6
6	Zhao 2023 (38)	END	0.601 (0.473, 0.730)	NR	NR	NR
7	Wang ZT 2023 (52)	END	0.845 (0.772, 0.918)	1,429	71.9	93.5
5. SAP						
1	Zhou 2024 (19)	SAP	0.807 (0.751, 0.855)	846.55	74.58	79.17
2	Tianlu 2024 (24)	SAP	0.723 (0.643, 0.802)	1179.56	62.50	79.44
3	Zhong 2021 (79)	SAP	0.742 (0.673, 0.812)	700	73.9	66.9
4	Cheng 2021 (77)	SAP	0.843 (0.798, 0.882)	885.05	79.5	85.0
5	Wei L 2021 (76)	SAP	0.801 (0.742, 0.852)	NR	NR	NR
6	Ceng 2020 (2) (80)	SAP	0.762 (0.736, 0.787)	901.06	68.67	78.00
6. PSD						
1	Dong 2024 (28)	PSD	0.765 (0.709, 0.820)	478.18	75.7	67.6
2	Song 2023 (49)	PSD	0.827 (0.736, 0.918)	NR	NR	NR
3	Hu 2021 (72)	PSD	0.579 (0.517, 0.641)	565.7	NR	NR
7. Moderate to Severe Disability(mRS3-5)						
1	Ceng 2020(1) (80)	mRS3-5(90d)	0.557 (NR, NR)	1148.4	26.8	87
2	Ceng 2020(2) (80)	mRS3-5(1y)	0.575 (NR, NR)	1179.43	25	88.4
8. AIS Severity						
1	Fernández-Garza 2023 (35)	AIS Severity	0.693 (0.599, 0.786)	623.723	73.5	67.3
9. Others						
1	Shao 2023 (47)	Moderate—Severe BG-EPVS	0.717 (0.638, 0.796)	686.35	47.8	91.2
2	Ji 2022 (62)	Malignant Cerebral Edema	0.69 (0.66, 0.73)	2,144	55	80
3	Zhang MK 2024 (32)	Failure of Delayed Neurological Improvement	0.861 (0.816, 0.907)	696.165	NR	NR
4	Wang ZT 2023 (52)	Early neurological improvement	0.58 (0.511, 0.648)	639.9	55.8	57.3
5	Li 2022 (58)	Decompressive Craniectomy	0.649 (NR, NR)	2505.7	55	75.8
6	Zheng 2024 (33)	Cerebral Herniation	0.794 (0.636, 0.953)	1798	85.7	68
7	Xiao 2023 (43)	Patent Foramen Ovale	0.777 (0.674, 0.861)	476.4	70	70
8	Zhang LL 2024 (15)	Ulcerative Plaque	0.895 (NR, NR)	537.4	93.3	89.2
9	Hao 2024 (11)	Stroke-heart Syndrome	0.767 (0.6443, 0.8892)	857	66.67	83.51
10	Zhang J 2024 (27)	Progressive Ischemic Stroke	0.656 (0.535, 0.778)	737.624	40.0	92.9
11	Cheng 2024 (9)	Post-stroke Cognitive Impairment	0.659 (0.600, 0.717)	676.83	44.6	82.0

SII, Systemic Immune-inflammation Index; AUC, Area Under the Curve; NR, Not Reported; AIS, Acute Ischemic Stroke; HT, Hemorrhagic Transformation; sICH, Symptomatic Intracerebral Hemorrhage; END, Early Neurological Deterioration; SAP, Stroke-Associated Pneumonia; PSD, Post-stroke Depression; mRS, Modified Rankin Scale; BG-EPVS, Basal Ganglia Region Enlarged Perivascular Spaces; h, hours; d, day; y, year; mos, month(s); w, week.

## 4 Discussion

In this study, we conducted a comprehensive systematic review and meta-analysis to explore the link between SII and AIS. A total of 40,682 individuals from 78 studies (1, 5–33, 35–82) were involved in the meta-analysis, while 79 studies (1, 5–82) were included in the systematic review.

The principal findings of this study are as follows: (1) The continuous SII values in poor prognosis, death, moderate–severe severity, HT/sICH, SAP/PSP, END, PSD, Progression/Recurrence groups were significantly higher than those in favorable prognosis, survival, mild severity, non-HT/sICH, non-SAP/PSP, non-END, non-PSD, no-progression/recurrence groups. (2) The incidence of poor prognosis, mortality, moderate–severe severity, HT/sICH, SAP/PSP, and END could be higher with an increase in continuous SII, significantly higher except for the incidence related to severity. (3) The sample size of poor prognosis, death, HT/sICH, END, progression/recurrence patients of high SII groups was significantly higher than that of low SII groups. (4) The risk of mortality, severity, HT/sICH, SAP/PSP, END, PSD, Progression/Recurrence in high SII groups was higher than in low SII groups, significantly higher except for the risks of poor prognosis and END. (5) The Admission NIHSS in AIS patients with high SII groups was significantly higher than in low SII groups.

From a pathophysiological perspective, the body’s immune-inflammatory response is activated following the onset of AIS. SII, a biomarker of systemic immune inflammation, has an elevated SII level that often implies an exacerbated inflammatory response, triggering a cascade of adverse events (84). Inflammatory cells infiltrate the brain tissue, releasing diverse inflammatory factors that disrupt the blood–brain barrier, exacerbate brain edema, and intensify neurological damage (83). Additionally, high SII levels are associated with platelet activation and aggregation, promoting thrombosis, aggravating cerebral ischemia, and influencing AIS prognosis, mortality, severity, END, progression, and recurrence (2–4). Patients in high SII groups are at a significantly higher risk of developing HT (64), likely due to high-SII-induced vascular endothelial damage, increased vascular permeability, and blood component exudation. Patients in High SII groups are also more susceptible to PSD (72), as the inflammatory response interferes with neurotransmitter synthesis, metabolism, and release, leading to an imbalance in neurotransmitters like 5-hydroxytryptamine and dopamine. Moreover, high SII levels, reflecting a perturbed immune-inflammatory state, increase the risk of SAP by reducing the body’s resistance and making it more vulnerable to pulmonary infections (76, 80).

Our study boasts noteworthy strengths. First, given that the concept of the SII was first proposed by Chinese researchers (84), we specifically retrieved several Chinese databases as sources. This effort significantly broadened the scope of our system review. The search strategy we implemented was more sophisticated. For the research on AIS, our search keywords included 6 subject terms and 122 free terms, effectively reducing the probability of missed or inaccurate retrievals. By incorporating studies from more recent years, we broadened the scope further, guaranteeing the inclusion of the latest research findings. Moreover, our analysis encompassed additional outcomes, such as SAP/PSP, END, and PSD, which were integrated into the meta-analysis for the first time, facilitating a more multi-dimensional assessment.

There are several limitations to our study. First, language is a constraint, as we only included literature in Chinese and English, while relevant studies in other languages may contain valuable information, affecting the generalizability and comprehensiveness of the findings. Second, due to the variability of cut-offs of SII used in different studies, we could not determine a consensus on the best cut-off value based on our analysis, which may limit clinical guidance. Third, although we used various methods to assess and deal with heterogeneity, some analysis results still have high heterogeneity, which may affect the accuracy and reliability of pooled effect values, reducing the persuasiveness of the findings. Sources of heterogeneity may include differences in study participants (age, sex, nationality, etc.), differences in study design (prospective cohort studies, retrospective cohort studies, and case–control studies), differences in interventions (different treatments, drug use, etc.), differences in SII grouping criteria (time of blood sampling and instruments), and differences in outcome measures (definitions and evaluation tools).

This result suggests that SII levels may represent an important diagnostic and prognostic tool for AIS complications in clinical practice. Monitoring and treatment should be strengthened for patients with higher SII levels, and more active measures should be taken to control the inflammatory response and clotting state. However, the role of SII in predicting poor prognosis, mortality, severity, and a variety of other complications is not fully understood.

In summary, high SII levels are linked to poor AIS prognosis and multiple complications, and SII may function as a cost-effective prognostic biomarker. Evaluating the role of SII in therapeutic decision-making is necessary, as our preliminary results suggest its potential to reflect clinical conditions and assist decision-makers. However, more research, especially large-sample and multi-center studies, is needed to better understand the utility of SII through dynamic monitoring.

## Data Availability

The raw data supporting the conclusions of this article will be made available by the authors, without undue reservation.
