# Supplementary material for: Prognostic assessment of acute ischemic stroke by systemic immune-inflammatory index: a comprehensive meta-analysis of multidimensional outcomes
Source: Front Neurol. 2025 Oct 20;16:1594258. doi: 10.3389/fneur.2025.1594258 (PMC12580097; doi:10.3389/fneur.2025.1594258)

# Supplementary 1:Search Strategy and Results

ALL=1646

PUBMED 219+WOS 295+EMBASE 332+Cochrane 20+EBSCO 33+SCOPUS 262+OVID 193+CNKI 81+WANFANG 115+VIP 0+sinomed 75+ClinicalTrials 19+WHO-ICTRP 0+Chictr 2+DANS EASY 0

# PUBMED:219

Search: ****(((((((Brain Infarction[MeSH Terms]) OR (Brain Ischemia[MeSH Terms])) OR (Cerebral Arterial Diseases[MeSH Terms])) OR (Cerebral Infarction[MeSH Terms])) OR (Cerebrovascular Disorders[MeSH Terms])) OR (Stroke[MeSH Terms])) OR ((((((((((((((((((((((((((((((((((((((((((((((((((((((((((((((((((((((((((((((((((((((((((((((((((((((((((((((((((((((((((((((((Acute Cerebral Infarction[Title/Abstract]) OR (Acute Cerebrovascular Accident[Title/Abstract])) OR (Acute Cerebrovascular Accidents[Title/Abstract])) OR (Acute Cerebrovascular Lesion[Title/Abstract])) OR (Acute Focal Cerebral Vasculopathy[Title/Abstract])) OR (Acute Ischemic Stroke[Title/Abstract])) OR (Acute Stroke[Title/Abstract])) OR (Acute Strokes[Title/Abstract])) OR (Apoplectic Hemiplegia[Title/Abstract])) OR (Apoplectic Stroke[Title/Abstract])) OR (Apoplexia[Title/Abstract])) OR (Apoplexy[Title/Abstract])) OR (Apoplexy, Cerebrovascular[Title/Abstract])) OR (Blood Flow Disturbance, Brain[Title/Abstract])) OR (Brain Accident[Title/Abstract])) OR (Brain Angiopathy[Title/Abstract])) OR (Brain Attack[Title/Abstract])) OR (Brain Blood Flow Disturbance[Title/Abstract])) OR (Brain Circulation Failure[Title/Abstract])) OR (Brain Infarct[Title/Abstract])) OR (Brain Infarction[Title/Abstract])) OR (Brain Infarctions[Title/Abstract])) OR (Brain Infarcts[Title/Abstract])) OR (Brain Insult[Title/Abstract])) OR (Brain Insultus[Title/Abstract])) OR (Brain Ischemia[Title/Abstract])) OR (Brain Ischemias[Title/Abstract])) OR (Brain Vascular Accident[Title/Abstract])) OR (Brain Vascular Accidents[Title/Abstract])) OR (Brain Vascular Disease[Title/Abstract])) OR (Brain Vascular Disorder[Title/Abstract])) OR (Brain Vascular Disorders[Title/Abstract])) OR (Brain Vasculopathy[Title/Abstract])) OR (Cerebral Apoplexia[Title/Abstract])) OR (Cerebral Arterial Diseases[Title/Abstract])) OR (Cerebral Infarct[Title/Abstract])) OR (Cerebral Infarction[Title/Abstract])) OR (Cerebral Infarctions[Title/Abstract])) OR (Cerebral Infarcts[Title/Abstract])) OR (Cerebral Insult[Title/Abstract])) OR (Cerebral Ischemia[Title/Abstract])) OR (Cerebral Ischemias[Title/Abstract])) OR (Cerebral Ischemic Stroke[Title/Abstract])) OR (Cerebral Stroke[Title/Abstract])) OR (Cerebral Strokes[Title/Abstract])) OR (Cerebral Vascular Accident[Title/Abstract])) OR (Cerebral Vascular Disease[Title/Abstract])) OR (Cerebral Vascular Disorder[Title/Abstract])) OR (Cerebral Vascular Disturbance[Title/Abstract])) OR (Cerebral Vascular Insufficiency[Title/Abstract])) OR (Cerebral Vascular Lesion[Title/Abstract])) OR (Cerebral Vasculopathy[Title/Abstract])) OR (Cerebro Vascular Accident[Title/Abstract])) OR (Cerebro Vascular Arrest[Title/Abstract])) OR (Cerebro Vascular Failure[Title/Abstract])) OR (Cerebro Vascular Injury[Title/Abstract])) OR (Cerebro Vascular Insufficiency[Title/Abstract])) OR (Cerebro Vascular Insult[Title/Abstract])) OR (Cerebrovascular Accident[Title/Abstract])) OR (Cerebrovascular Accident, Acute[Title/Abstract])) OR (Cerebrovascular Accidents[Title/Abstract])) OR (Cerebrovascular Accidents, Acute[Title/Abstract])) OR (Cerebrovascular Apoplexy[Title/Abstract])) OR (Cerebrovascular Damage[Title/Abstract])) OR (Cerebrovascular Disease[Title/Abstract])) OR (Cerebrovascular Diseases[Title/Abstract])) OR (Cerebrovascular Disorder[Title/Abstract])) OR (Cerebrovascular Disorders[Title/Abstract])) OR (Cerebrovascular Insufficiencies[Title/Abstract])) OR (Cerebrovascular Insufficiency[Title/Abstract])) OR (Cerebrovascular Lesion[Title/Abstract])) OR (Cerebrovascular Occlusion[Title/Abstract])) OR (Cerebrovascular Occlusions[Title/Abstract])) OR (Cerebrovascular Stroke[Title/Abstract])) OR (Cerebrovascular Strokes[Title/Abstract])) OR (Cerebrovascular Syndrome Accident[Title/Abstract])) OR (Cerebrum Vascular Accident[Title/Abstract])) OR (Cryptogenic Stroke[Title/Abstract])) OR (CVA[Title/Abstract])) OR (CVAs[Title/Abstract])) OR (Disease, Cerebrovascular[Title/Abstract])) OR (Diseases, Cerebrovascular[Title/Abstract])) OR (Encephalopathy, Ischemic[Title/Abstract])) OR (Focal Cerebral Ischemia[Title/Abstract])) OR (Infarct, Brain[Title/Abstract])) OR (Infarct, Cerebral[Title/Abstract])) OR (Infarction, Brain[Title/Abstract])) OR (Infarction, Cerebral[Title/Abstract])) OR (Infarctions, Brain[Title/Abstract])) OR (Infarctions, Cerebral[Title/Abstract])) OR (Infarcts, Brain[Title/Abstract])) OR (Infarcts, Cerebral[Title/Abstract])) OR (Insufficiencies, Cerebrovascular[Title/Abstract])) OR (Insufficiency, Cerebrovascular[Title/Abstract])) OR (Intracranial Vascular Disease[Title/Abstract])) OR (Intracranial Vascular Disorder[Title/Abstract])) OR (Intracranial Vascular Disorders[Title/Abstract])) OR (Ischemia, Brain[Title/Abstract])) OR (Ischemia, Cerebral[Title/Abstract])) OR (Ischemias, Cerebral[Title/Abstract])) OR (Ischemic Cerebrovascular Disease[Title/Abstract])) OR (Ischemic Brain Injury[Title/Abstract])) OR (Ischemic Cerebrovascular Disease[Title/Abstract])) OR (Ischemic Encephalopathies[Title/Abstract])) OR (Ischemic Encephalopathy[Title/Abstract])) OR (Ischemic Stroke[Title/Abstract])) OR (Massive Cerebral Infarction[Title/Abstract])) OR (Multiple Cerebral Infarction[Title/Abstract])) OR (Occlusion, Cerebrovascular[Title/Abstract])) OR (Occlusions, Cerebrovascular[Title/Abstract])) OR (Occlusive Cerebrovascular Disease[Title/Abstract])) OR (Stroke[Title/Abstract])) OR (Stroke, Acute[Title/Abstract])) OR (Stroke, Cerebral[Title/Abstract])) OR (Stroke, Cerebrovascular[Title/Abstract])) OR (Strokes[Title/Abstract])) OR (Strokes, Acute[Title/Abstract])) OR (Strokes, Cerebral[Title/Abstract])) OR (Strokes, Cerebrovascular[Title/Abstract])) OR (Thrombotic Stroke[Title/Abstract])) OR (Vascular Accident, Brain[Title/Abstract])) OR (Vascular Accidents, Brain[Title/Abstract])) OR (Vascular Disease, Intracranial[Title/Abstract])) OR (Vascular Diseases, Intracranial[Title/Abstract])) OR (Vascular Disorder, Brain[Title/Abstract])) OR (Vascular Disorder, Intracranial[Title/Abstract])) OR (Vascular Disorders, Brain[Title/Abstract])) OR (Vascular Disorders, Intracranial[Title/Abstract]))) AND ((((((systemic immune inflammatory index[Title/Abstract]) OR (systemic immune-inflammatory index[Title/Abstract])) OR (SII[Title/Abstract])) OR (Systemic-immune-inflammation index[Title/Abstract])) OR (Systemic immune-inflammation index[Title/Abstract])) OR (neutrophil ×platelets/lymphocyte[Title/Abstract]))**** Filters: ****from 1900/1/1 - 2024/10/13**** Sort by: ****Most Recent****


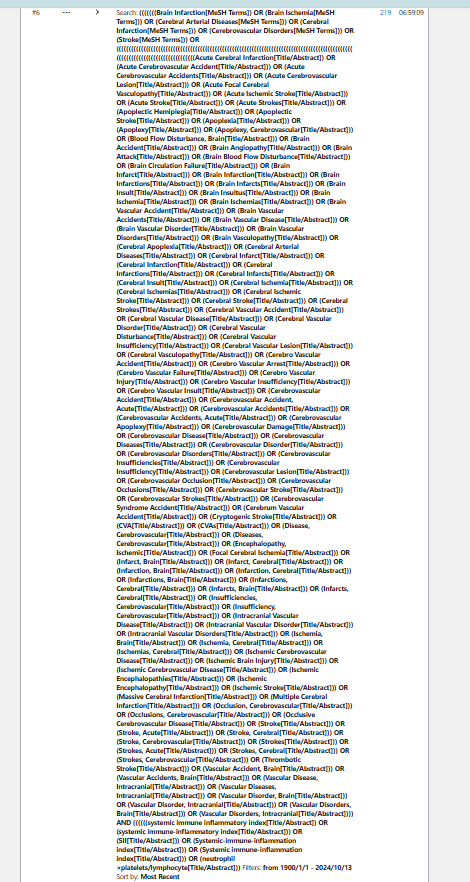


# WOS:295

****#1****

****TS=(systemic immune inflammatory index OR systemic immune-inflammatory index OR SII OR Systemic-immune-inflammation index OR Systemic immune-inflammation index OR neutrophil ×platelets/lymphocyte)****

****#2****

****TS=(Acute Cerebral Infarction OR Acute Cerebrovascular Accident OR Acute Cerebrovascular Accidents OR Acute Cerebrovascular Lesion OR Acute Focal Cerebral Vasculopathy OR Acute Ischemic Stroke OR Acute Stroke OR Acute Strokes OR Apoplectic Hemiplegia OR Apoplectic Stroke OR Apoplexia OR Apoplexy OR Apoplexy, Cerebrovascular OR Blood Flow Disturbance, Brain OR Brain Accident OR Brain Angiopathy OR Brain Attack OR Brain Blood Flow Disturbance OR Brain Circulation Failure OR Brain Infarct OR Brain Infarction OR Brain Infarctions OR Brain Infarcts OR Brain Insult OR Brain Insultus OR Brain Ischemia OR Brain Ischemias OR Brain Vascular Accident OR Brain Vascular Accidents OR Brain Vascular Disease OR Brain Vascular Disorder OR Brain Vascular Disorders OR Brain Vasculopathy OR Cerebral Apoplexia OR Cerebral Arterial Diseases OR Cerebral Infarct OR Cerebral Infarction OR Cerebral Infarctions OR Cerebral Infarcts OR Cerebral Insult OR Cerebral Ischemia OR Cerebral Ischemias OR Cerebral Ischemic Stroke OR Cerebral Stroke OR Cerebral Strokes OR Cerebral Vascular Accident OR Cerebral Vascular Disease OR Cerebral Vascular Disorder OR Cerebral Vascular Disturbance OR Cerebral Vascular Insufficiency OR Cerebral Vascular Lesion OR Cerebral Vasculopathy OR Cerebro Vascular Accident OR Cerebro Vascular Arrest OR Cerebro Vascular Failure OR Cerebro Vascular Injury OR Cerebro Vascular Insufficiency OR Cerebro Vascular Insult OR Cerebrovascular Accident OR Cerebrovascular Accident, Acute OR Cerebrovascular Accidents OR Cerebrovascular Accidents, Acute OR Cerebrovascular Apoplexy OR Cerebrovascular Damage OR Cerebrovascular Disease OR Cerebrovascular Diseases OR Cerebrovascular Disorder OR Cerebrovascular Disorders OR Cerebrovascular Insufficiencies OR Cerebrovascular Insufficiency OR Cerebrovascular Lesion OR Cerebrovascular Occlusion OR Cerebrovascular Occlusions OR Cerebrovascular Stroke OR Cerebrovascular Strokes OR Cerebrovascular Syndrome Accident OR Cerebrum Vascular Accident OR Cryptogenic Stroke OR CVA OR CVAs OR Disease, Cerebrovascular OR Diseases, Cerebrovascular OR Encephalopathy, Ischemic OR Focal Cerebral Ischemia OR Infarct, Brain OR Infarct, Cerebral OR Infarction, Brain OR Infarction, Cerebral OR Infarctions, Brain OR Infarctions, Cerebral OR Infarcts, Brain OR Infarcts, Cerebral OR Insufficiencies, Cerebrovascular OR Insufficiency, Cerebrovascular OR Intracranial Vascular Disease OR Intracranial Vascular Disorder OR Intracranial Vascular Disorders OR Ischemia, Brain OR Ischemia, Cerebral OR Ischemias, Cerebral OR Ischemic Cerebrovascular Disease OR Ischemic Brain Injury OR Ischemic Cerebrovascular Disease OR Ischemic Encephalopathies OR Ischemic Encephalopathy OR Ischemic Stroke OR Massive Cerebral Infarction OR Multiple Cerebral Infarction OR Occlusion, Cerebrovascular OR Occlusions, Cerebrovascular OR Occlusive Cerebrovascular Disease OR Stroke OR Stroke, Acute OR Stroke, Cerebral OR Stroke, Cerebrovascular OR Strokes OR Strokes, Acute OR Strokes, Cerebral OR Strokes, Cerebrovascular OR Thrombotic Stroke OR Vascular Accident, Brain OR Vascular Accidents, Brain OR Vascular Disease, Intracranial OR Vascular Diseases, Intracranial OR Vascular Disorder, Brain OR Vascular Disorder, Intracranial OR Vascular Disorders, Brain OR Vascular Disorders, Intracranial)****

****#3****

****#1 AND #2****


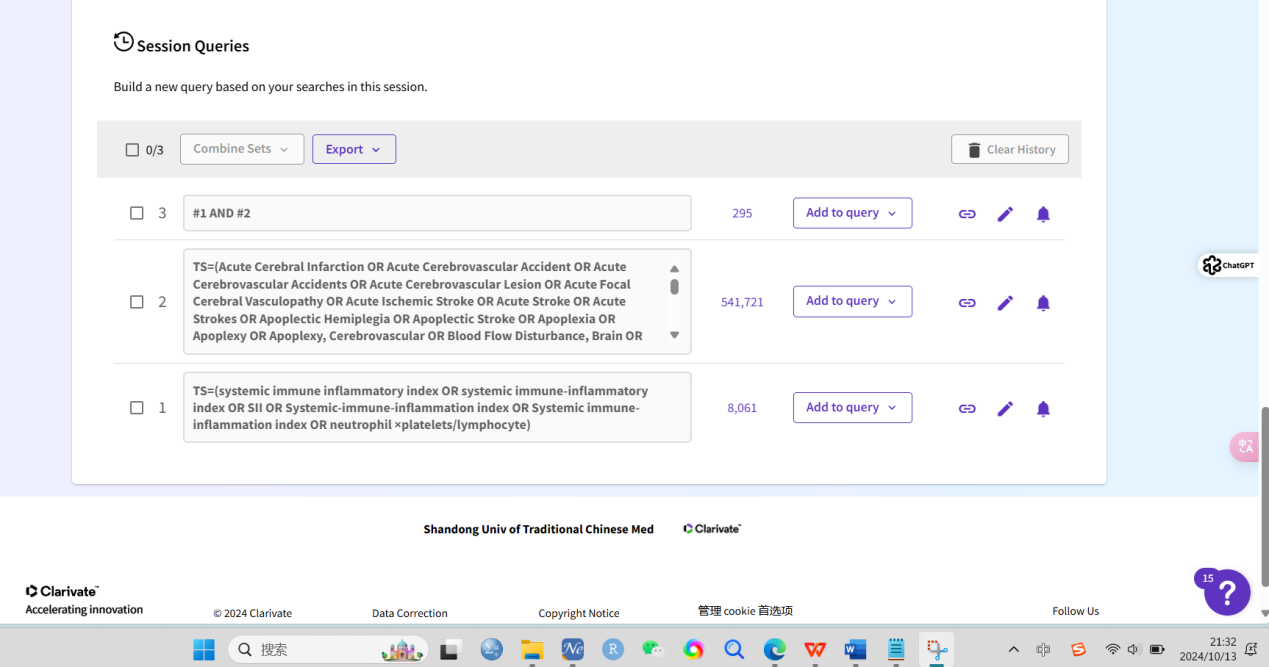


# EMBASE:332

#1

**'brain infarction'/exp OR 'cerebrovascular accident'/exp OR 'cerebrovascular disease'/exp OR 'brain ischemia'/exp OR 'occlusive cerebrovascular disease'/exp OR 'cerebral artery disease'/exp OR** 'Acute Cerebral Infarction':ab,ti or 'Acute Cerebrovascular Accident':ab,ti or 'Acute Cerebrovascular Accidents':ab,ti or 'Acute Cerebrovascular Lesion':ab,ti or 'Acute Focal Cerebral Vasculopathy':ab,ti or 'Acute Ischemic Stroke':ab,ti or 'Acute Stroke':ab,ti or 'Acute Strokes':ab,ti or 'Apoplectic Hemiplegia':ab,ti or 'Apoplectic Stroke':ab,ti or 'Apoplexia':ab,ti or 'Apoplexy':ab,ti or 'Apoplexy, Cerebrovascular':ab,ti or 'Blood Flow Disturbance, Brain':ab,ti or 'Brain Accident':ab,ti or 'Brain Angiopathy':ab,ti or 'Brain Attack':ab,ti or 'Brain Blood Flow Disturbance':ab,ti or 'Brain Circulation Failure':ab,ti or 'Brain Infarct':ab,ti or 'Brain Infarction':ab,ti or 'Brain Infarctions':ab,ti or 'Brain Infarcts':ab,ti or 'Brain Insult':ab,ti or 'Brain Insultus':ab,ti or 'Brain Ischemia':ab,ti or 'Brain Ischemias':ab,ti or 'Brain Vascular Accident':ab,ti or 'Brain Vascular Accidents':ab,ti or 'Brain Vascular Disease':ab,ti or 'Brain Vascular Disorder':ab,ti or 'Brain Vascular Disorders':ab,ti or 'Brain Vasculopathy':ab,ti or 'Cerebral Apoplexia':ab,ti or 'Cerebral Arterial Diseases':ab,ti or 'Cerebral Infarct':ab,ti or 'Cerebral Infarction':ab,ti or 'Cerebral Infarctions':ab,ti or 'Cerebral Infarcts':ab,ti or 'Cerebral Insult':ab,ti or 'Cerebral Ischemia':ab,ti or 'Cerebral Ischemias':ab,ti or 'Cerebral Ischemic Stroke':ab,ti or 'Cerebral Stroke':ab,ti or 'Cerebral Strokes':ab,ti or 'Cerebral Vascular Accident':ab,ti or 'Cerebral Vascular Disease':ab,ti or 'Cerebral Vascular Disorder':ab,ti or 'Cerebral Vascular Disturbance':ab,ti or 'Cerebral Vascular Insufficiency':ab,ti or 'Cerebral Vascular Lesion':ab,ti or 'Cerebral Vasculopathy':ab,ti or 'Cerebro Vascular Accident':ab,ti or 'Cerebro Vascular Arrest':ab,ti or 'Cerebro Vascular Failure':ab,ti or 'Cerebro Vascular Injury':ab,ti or 'Cerebro Vascular Insufficiency':ab,ti or 'Cerebro Vascular Insult':ab,ti or 'Cerebrovascular Accident':ab,ti or 'Cerebrovascular Accident, Acute':ab,ti or 'Cerebrovascular Accidents':ab,ti or 'Cerebrovascular Accidents, Acute':ab,ti or 'Cerebrovascular Apoplexy':ab,ti or 'Cerebrovascular Damage':ab,ti or 'Cerebrovascular Disease':ab,ti or 'Cerebrovascular Diseases':ab,ti or 'Cerebrovascular Disorder':ab,ti or 'Cerebrovascular Disorders':ab,ti or 'Cerebrovascular Insufficiencies':ab,ti or 'Cerebrovascular Insufficiency':ab,ti or 'Cerebrovascular Lesion':ab,ti or 'Cerebrovascular Occlusion':ab,ti or 'Cerebrovascular Stroke':ab,ti or 'Cerebrovascular Strokes':ab,ti or 'Cerebrovascular Syndrome Accident':ab,ti or 'Cerebrum Vascular Accident':ab,ti or 'Cryptogenic Stroke':ab,ti or 'CVA':ab,ti or 'CVAs':ab,ti or 'Disease, Cerebrovascular':ab,ti or 'Diseases, Cerebrovascular':ab,ti or 'Encephalopathy, Ischemic':ab,ti or 'Focal Cerebral Ischemia':ab,ti or 'Infarct, Brain':ab,ti or 'Infarct, Cerebral':ab,ti or 'Infarction, Brain':ab,ti or 'Infarction, Cerebral':ab,ti or 'Infarctions, Brain':ab,ti or 'Infarctions, Cerebral':ab,ti or 'Infarcts, Brain':ab,ti or 'Infarcts, Cerebral':ab,ti or 'Insufficiencies, Cerebrovascular':ab,ti or 'Insufficiency, Cerebrovascular':ab,ti or 'Intracranial Vascular Disease':ab,ti or 'Intracranial Vascular Disorder':ab,ti or 'Intracranial Vascular Disorders':ab,ti or 'Ischemia, Brain':ab,ti or 'Ischemia, Cerebral':ab,ti or 'Ischemias, Cerebral':ab,ti or 'Ischemic Cerebrovascular Disease':ab,ti or 'Ischemic Brain Injury':ab,ti or 'Ischemic Cerebrovascular Disease':ab,ti or 'Ischemic Encephalopathies':ab,ti or 'Ischemic Encephalopathy':ab,ti or 'Ischemic Stroke':ab,ti or 'Massive Cerebral Infarction':ab,ti or 'Multiple Cerebral Infarction':ab,ti or 'Occlusion, Cerebrovascular':ab,ti or 'Occlusions, Cerebrovascular':ab,ti or 'Occlusive Cerebrovascular Disease':ab,ti or 'Stroke':ab,ti or 'Stroke, Acute':ab,ti or 'Stroke, Cerebral':ab,ti or 'Stroke, Cerebrovascular':ab,ti or 'Strokes':ab,ti or 'Strokes, Acute':ab,ti or 'Strokes, Cerebral':ab,ti or 'Strokes, Cerebrovascular':ab,ti or 'Thrombotic Stroke':ab,ti or 'Vascular Accident, Brain':ab,ti or 'Vascular Accidents, Brain':ab,ti or 'Vascular Disease, Intracranial':ab,ti or 'Vascular Diseases, Intracranial':ab,ti or 'Vascular Disorder, Brain':ab,ti or 'Vascular Disorder, Intracranial':ab,ti or 'Vascular Disorders, Brain':ab,ti or 'Vascular Disorders, Intracranial':ab,ti

#2

'systemic immune inflammatory index'/exp OR 'systemic immune inflammatory index':ab,ti OR 'systemic immune-inflammatory index':ab,ti OR 'SII ':ab,ti OR 'Systemic-immune-inflammation index':ab,ti OR 'Systemic immune-inflammation index

':ab,ti OR 'neutrophil ×platelets/lymphocyte':ab,ti

#3

#1 AND #2 AND [01-01-1966]/sd NOT [14-10-2024]/sd


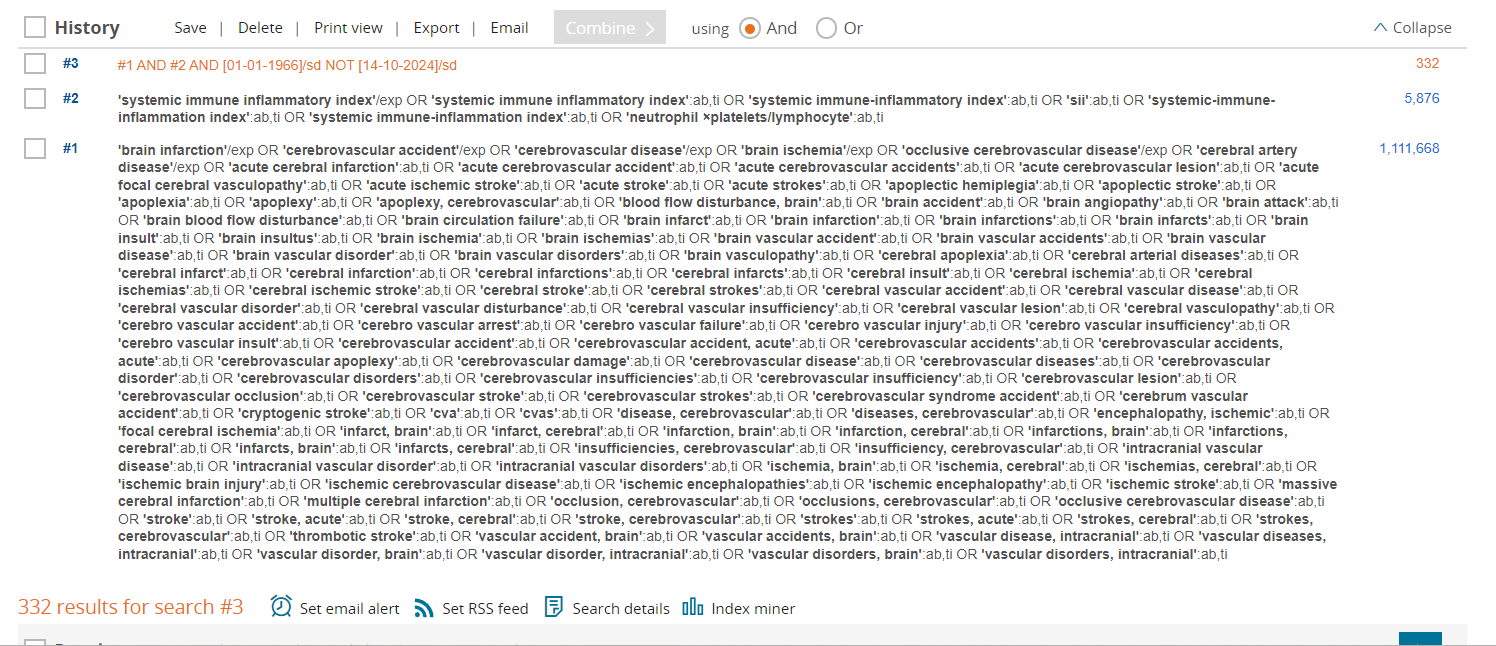


# Cochrane:20

#1 MeSH descriptor: [Cerebral Infarction] explode all trees

#2 MeSH descriptor: [Stroke] explode all trees

#3 MeSH descriptor: [Brain Infarction] explode all trees

#4 MeSH descriptor: [Cerebrovascular Disorders] explode all trees

#5 MeSH descriptor: [Brain Ischemia] explode all trees

#6 MeSH descriptor: [Cerebral Arterial Diseases] explode all trees

#7 (Acute Cerebral Infarction OR Acute Cerebrovascular Accident OR Acute Cerebrovascular Accidents OR Acute Cerebrovascular Lesion OR Acute Focal Cerebral Vasculopathy OR Acute Ischemic Stroke OR Acute Stroke OR Acute Strokes OR Apoplectic Hemiplegia OR Apoplectic Stroke OR Apoplexia OR Apoplexy OR Apoplexy, Cerebrovascular OR Blood Flow Disturbance, Brain OR Brain Accident OR Brain Angiopathy OR Brain Attack OR Brain Blood Flow Disturbance OR Brain Circulation Failure OR Brain Infarct OR Brain Infarction OR Brain Infarctions OR Brain Infarcts OR Brain Insult OR Brain Insultus OR Brain Ischemia OR Brain Ischemias OR Brain Vascular Accident OR Brain Vascular Accidents OR Brain Vascular Disease OR Brain Vascular Disorder OR Brain Vascular Disorders OR Brain Vasculopathy OR Cerebral Apoplexia OR Cerebral Arterial Diseases OR Cerebral Infarct OR Cerebral Infarction OR Cerebral Infarctions OR Cerebral Infarcts OR Cerebral Insult OR Cerebral Ischemia OR Cerebral Ischemias OR Cerebral Ischemic Stroke OR Cerebral Stroke OR Cerebral Strokes OR Cerebral Vascular Accident OR Cerebral Vascular Disease OR Cerebral Vascular Disorder OR Cerebral Vascular Disturbance OR Cerebral Vascular Insufficiency OR Cerebral Vascular Lesion OR Cerebral Vasculopathy OR Cerebro Vascular Accident OR Cerebro Vascular Arrest OR Cerebro Vascular Failure OR Cerebro Vascular Injury OR Cerebro Vascular Insufficiency OR Cerebro Vascular Insult OR Cerebrovascular Accident OR Cerebrovascular Accident, Acute OR Cerebrovascular Accidents OR Cerebrovascular Accidents, Acute OR Cerebrovascular Apoplexy OR Cerebrovascular Damage OR Cerebrovascular Disease OR Cerebrovascular Diseases OR Cerebrovascular Disorder OR Cerebrovascular Disorders OR Cerebrovascular Insufficiencies OR Cerebrovascular Insufficiency OR Cerebrovascular Lesion OR Cerebrovascular Occlusion OR Cerebrovascular Occlusions OR Cerebrovascular Stroke OR Cerebrovascular Strokes OR Cerebrovascular Syndrome Accident OR Cerebrum Vascular Accident OR Cryptogenic Stroke OR CVA OR CVAs OR Disease, Cerebrovascular OR Diseases, Cerebrovascular OR Encephalopathy, Ischemic OR Focal Cerebral Ischemia OR Infarct, Brain OR Infarct, Cerebral OR Infarction, Brain OR Infarction, Cerebral OR Infarctions, Brain OR Infarctions, Cerebral OR Infarcts, Brain OR Infarcts, Cerebral OR Insufficiencies, Cerebrovascular OR Insufficiency, Cerebrovascular OR Intracranial Vascular Disease OR Intracranial Vascular Disorder OR Intracranial Vascular Disorders OR Ischemia, Brain OR Ischemia, Cerebral OR Ischemias, Cerebral OR Ischemic Cerebrovascular Disease OR Ischemic Brain Injury OR Ischemic Cerebrovascular Disease OR Ischemic Encephalopathies OR Ischemic Encephalopathy OR Ischemic Stroke OR Massive Cerebral Infarction OR Multiple Cerebral Infarction OR Occlusion, Cerebrovascular OR Occlusions, Cerebrovascular OR Occlusive Cerebrovascular Disease OR Stroke OR Stroke, Acute OR Stroke, Cerebral OR Stroke, Cerebrovascular OR Strokes OR Strokes, Acute OR Strokes, Cerebral OR Strokes, Cerebrovascular OR Thrombotic Stroke OR Vascular Accident, Brain OR Vascular Accidents, Brain OR Vascular Disease, Intracranial OR Vascular Diseases, Intracranial OR Vascular Disorder, Brain OR Vascular Disorder, Intracranial OR Vascular Disorders, Brain OR Vascular Disorders, Intracranial):ti,ab,kw

#8 #1 OR #2 OR #3 OR #4 OR #5 OR #6 or #7

#9 (systemic immune inflammatory index OR systemic immune-inflammatory index OR SII OR Systemic-immune-inflammation index OR Systemic immune-inflammation index OR (neutrophil AND platelets AND lymphocyte)):ti,ab,kw

#10 #8 AND #9

with Cochrane Library publication date from Jan 1000 to Oct 2024


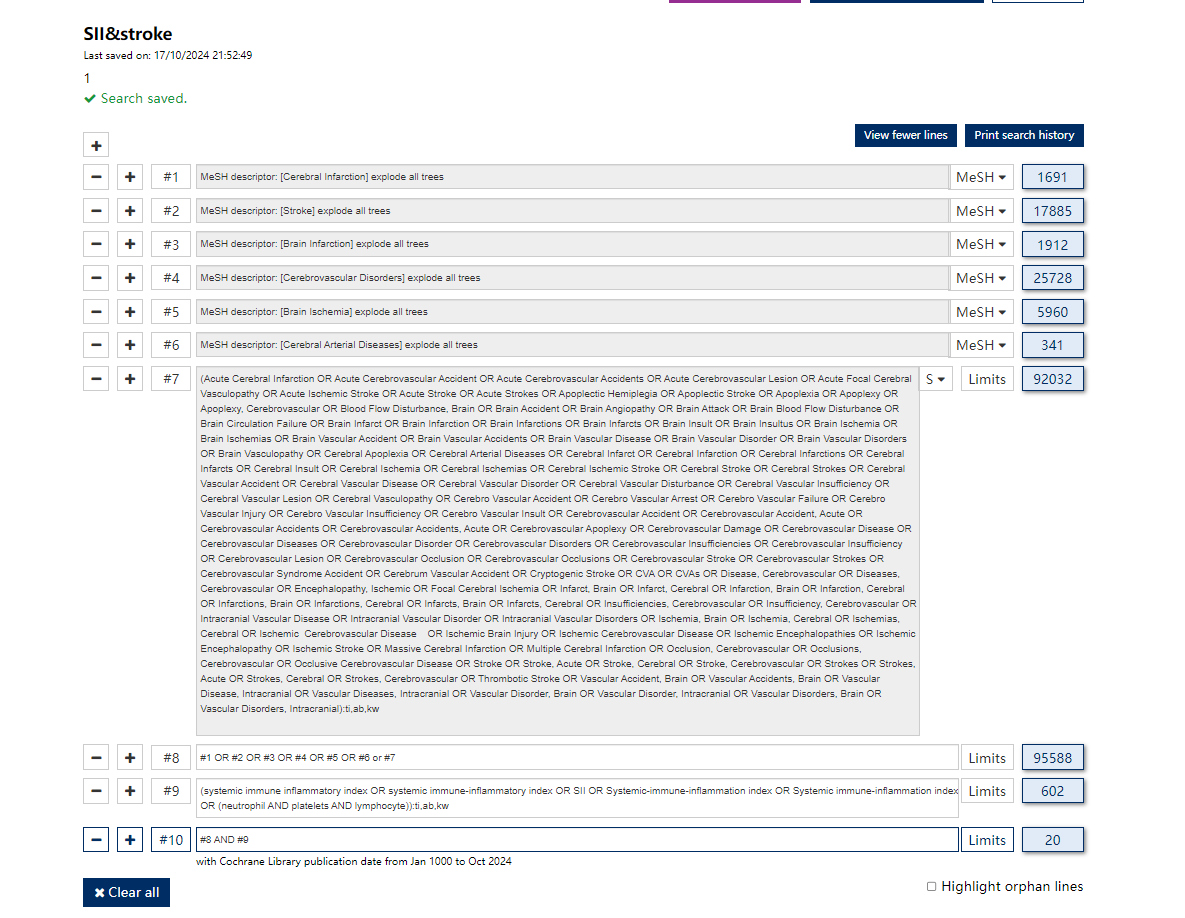


# CINAHL Plus with Full Text(EBSCO) : 33

S20 S18 AND S19

S19 TX systemic immune inflammatory index OR TX systemic immune-inflammatory index OR TX SII OR TX Systemic-immune-inflammation index OR TX Systemic immune-inflammation index OR TX neutrophil ×platelets/lymphocyte

S18 S1 OR S2 OR S3 OR S4 OR S5 OR S6 OR S7 OR S8 OR S9 OR S10 OR S11 OR S12 OR S13 OR S14 OR S15 OR S16 OR S17

S17 SU Vascular Accident, Brain OR SU Vascular Accidents, Brain OR SU Vascular Disease, Intracranial OR SU Vascular Diseases, Intracranial OR SU Vascular Disorder, Brain OR SU Vascular Disorder, Intracranial OR SU Vascular Disorders, Brain OR SU Vascular Disorders, Intracranial

S16 SU Occlusion, Cerebrovascular OR SU Occlusions, Cerebrovascular OR SU Occlusive Cerebrovascular Disease OR SU Stroke OR SU Stroke, Acute OR SU Stroke, Cerebral OR SU Stroke, Cerebrovascular OR SU Strokes OR SU Strokes, Acute OR SU Strokes, Cerebral OR SU Strokes, Cerebrovascular OR SU Thrombotic Stroke

S15 SU Intracranial Vascular Disorders OR SU Ischemia, Brain OR SU Ischemia, Brain OR SU Ischemias, Cerebral OR SU Ischemic Cerebrovascular Disease OR SU Ischemic Brain Injury OR SU Ischemic Cerebrovascular Disease OR SU Ischemic Encephalopathies OR SU Ischemic Encephalopathy OR SU Ischemic Stroke OR SU Massive Cerebral Infarction OR SU Multiple Cerebral Infarction

S14 SU Infarct, Brain OR SU Infarct, Cerebral OR SU Infarction, Brain OR SU Infarction, Cerebral OR SU Infarctions, Brain OR SU Infarctions, Cerebral OR SU Infarcts, Brain OR SU Infarcts, Cerebral OR SU Insufficiencies, Cerebrovascular OR SU Insufficiency, Cerebrovascular OR SU Intracranial Vascular Disease OR SU Intracranial Vascular Disorder

S13 SU Cerebrovascular Occlusions OR SU Cerebrovascular Stroke OR SU Cerebrovascular Strokes OR SU Cerebrovascular Syndrome Accident OR SU Cerebrum Vascular Accident OR SU Cryptogenic Stroke OR SU CVA OR SU CVAs OR SU Disease, Cerebrovascular OR SU Diseases, Cerebrovascular OR SU Encephalopathy, Ischemic OR SU Focal Cerebral Ischemia

S12 SU Cerebrovascular Accidents OR SU Cerebrovascular Accidents, Acute OR SU Cerebrovascular Apoplexy OR SU Cerebrovascular Damage OR SU Cerebrovascular Disease OR SU Cerebrovascular Diseases OR SU Cerebrovascular Disorder OR SU Cerebrovascular Disorders OR SU Cerebrovascular Insufficiencies OR SU Cerebrovascular Insufficiency OR SU Cerebrovascular Lesion OR SU Cerebrovascular Occlusion

S11 SU Cerebral Vascular Disturbance OR SU Cerebral Vascular Insufficiency OR SU Cerebral Vascular Lesion OR SU Cerebral Vasculopathy OR SU Cerebro Vascular Accident OR SU Cerebro Vascular Arrest OR SU Cerebro Vascular Failure OR SU Cerebro Vascular Injury OR SU Cerebro Vascular Insufficiency OR SU Cerebro Vascular Insult OR SU Cerebrovascular Accident OR SU Cerebrovascular Accident, Acute

S10 SU Cerebral Infarction OR SU Cerebral Infarctions OR SU Cerebral Infarcts OR SU Cerebral Insult OR SU Cerebral Ischemia OR SU Cerebral Ischemias OR SU Cerebral Ischemic Stroke OR SU Cerebral Stroke OR SU Cerebral Strokes OR SU Cerebral Vascular Accident OR SU Cerebral Vascular Disease OR SU Cerebral Vascular Disorder

S9 SU Brain Insultus OR SU Brain Ischemia OR SU Brain Ischemias OR SU Brain Vascular Accident OR SU Brain Vascular Accidents OR SU Brain Vascular Disease OR SU Brain Vascular Disorder OR SU Brain Vascular Disorders OR SU Brain Vasculopathy OR SU Cerebral Apoplexia OR SU Cerebral Arterial Diseases OR SU Cerebral Infarct

S8 SU Apoplexy, Cerebrovascular OR SU Blood Flow Disturbance, Brain OR SU Brain Accident OR SU Brain Angiopathy OR SU Brain Attack OR SU Brain Blood Flow Disturbance OR SU Brain Circulation Failure OR SU Brain Infarct OR SU Brain Infarction OR SU Brain Infarctions OR SU Brain Infarcts OR SU Brain Insult

S7 SU Acute Cerebral Infarction OR SU Acute Cerebrovascular Accident OR SU Acute Cerebrovascular Accidents OR SU Acute Cerebrovascular Lesion OR SU Acute Focal Cerebral Vasculopathy OR SU Acute Ischemic Stroke OR SU Acute Stroke OR SU Acute Strokes OR SU Apoplectic Hemiplegia OR SU Apoplectic Stroke OR SU Apoplexia OR SU Apoplexy

S6 (MH "Brain Injuries")

S5 (MH "Cerebral Arterial Diseases")

S4 (MH "Cerebral Ischemia")

S3 (MH "Cerebrovascular Disorders")

S2(MH "Stroke")

S1(MH "Cerebral Infarction")


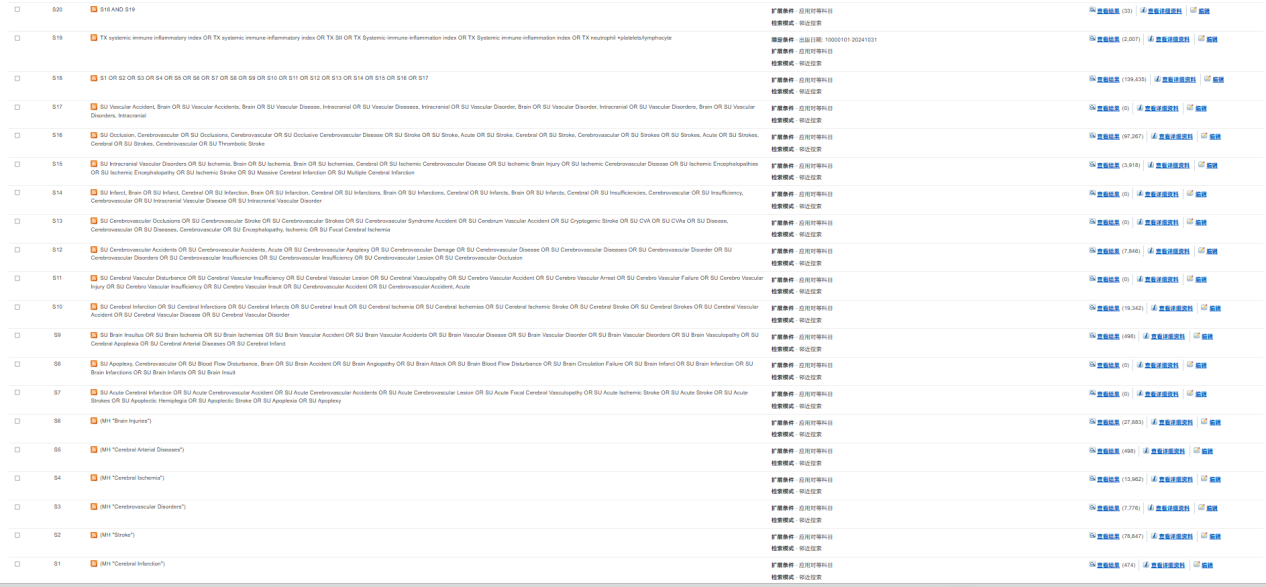


# SCOPUS:262

( TITLE-ABS-KEY ( "Acute Cerebral Infarction" OR "Acute Cerebrovascular Accident" OR "Acute Cerebrovascular Accidents" OR "Acute Cerebrovascular Lesion" OR "Acute Focal Cerebral Vasculopathy" OR "Acute Ischemic Stroke" OR "Acute Stroke" OR "Acute Strokes" OR "Apoplectic Hemiplegia" OR "Apoplectic Stroke" OR "Apoplexia" OR "Apoplexy" OR "Apoplexy, Cerebrovascular" OR "Blood Flow Disturbance, Brain" OR "Brain Accident" OR "Brain Angiopathy" OR "Brain Attack" OR "Brain Blood Flow Disturbance" OR "Brain Circulation Failure" OR "Brain Infarct" OR "Brain Infarction" OR "Brain Infarctions" OR "Brain Infarcts" OR "Brain Insult" OR "Brain Insultus" OR "Brain Ischemia" OR "Brain Ischemias" OR "Brain Vascular Accident" OR "Brain Vascular Accidents" OR "Brain Vascular Disease" OR "Brain Vascular Disorder" OR "Brain Vascular Disorders" OR "Brain Vasculopathy" OR "Cerebral Apoplexia" OR "Cerebral Arterial Diseases" OR "Cerebral Infarct" OR "Cerebral Infarction" OR "Cerebral Infarctions" OR "Cerebral Infarcts" OR "Cerebral Insult" OR "Cerebral Ischemia" OR "Cerebral Ischemias" OR "Cerebral Ischemic Stroke" OR "Cerebral Stroke" OR "Cerebral Strokes" OR "Cerebral Vascular Accident" OR "Cerebral Vascular Disease" OR "Cerebral Vascular Disorder" OR "Cerebral Vascular Disturbance" OR "Cerebral Vascular Insufficiency" OR "Cerebral Vascular Lesion" OR "Cerebral Vasculopathy" OR "Cerebro Vascular Accident" OR "Cerebro Vascular Arrest" OR "Cerebro Vascular Failure" OR "Cerebro Vascular Injury" OR "Cerebro Vascular Insufficiency" OR "Cerebro Vascular Insult" OR "Cerebrovascular Accident" OR "Cerebrovascular Accident, Acute" OR "Cerebrovascular Accidents" OR "Cerebrovascular Accidents, Acute" OR "Cerebrovascular Apoplexy" OR "Cerebrovascular Damage" OR "Cerebrovascular Disease" OR "Cerebrovascular Diseases" OR "Cerebrovascular Disorder" OR "Cerebrovascular Disorders" OR "Cerebrovascular Insufficiencies" OR "Cerebrovascular Insufficiency" OR "Cerebrovascular Lesion" OR "Cerebrovascular Occlusion" OR "Cerebrovascular Occlusions" OR "Cerebrovascular Stroke" OR "Cerebrovascular Strokes" OR "Cerebrovascular Syndrome Accident" OR "Cerebrum Vascular Accident" OR "Cryptogenic Stroke" OR "CVA" OR "CVAs" OR "Disease, Cerebrovascular" OR "Diseases, Cerebrovascular" OR "Encephalopathy, Ischemic" OR "Focal Cerebral Ischemia" OR "Infarct, Brain" OR "Infarct, Cerebral" OR "Infarction, Brain" OR "Infarction, Cerebral" OR "Infarctions, Brain" OR "Infarctions, Cerebral" OR "Infarcts, Brain" OR "Infarcts, Cerebral" OR "Insufficiencies, Cerebrovascular" OR "Insufficiency, Cerebrovascular" OR "Intracranial Vascular Disease" OR "Intracranial Vascular Disorder" OR "Intracranial Vascular Disorders" OR "Ischemia, Brain" OR "Ischemia, Cerebral" OR "Ischemias, Cerebral" OR "Ischemic Cerebrovascular Disease " OR "Ischemic Brain Injury" OR "Ischemic Cerebrovascular Disease" OR "Ischemic Encephalopathies" OR "Ischemic Encephalopathy" OR "Ischemic Stroke" OR "Massive Cerebral Infarction" OR "Multiple Cerebral Infarction" OR "Occlusion, Cerebrovascular" OR "Occlusions, Cerebrovascular" OR "Occlusive Cerebrovascular Disease" OR "Stroke" OR "Stroke, Acute" OR "Stroke, Cerebral" OR "Stroke, Cerebrovascular" OR "Strokes" OR "Strokes, Acute" OR "Strokes, Cerebral" OR "Strokes, Cerebrovascular" OR "Thrombotic Stroke" OR "Vascular Accident, Brain" OR "Vascular Accidents, Brain" OR "Vascular Disease, Intracranial" OR "Vascular Diseases, Intracranial" OR "Vascular Disorder, Brain" OR "Vascular Disorder, Intracranial" OR "Vascular Disorders, Brain" OR "Vascular Disorders, Intracranial" ) AND TITLE-ABS-KEY ( "systemic immune inflammatory index " OR "systemic immune-inflammatory index" OR "SII " OR "Systemic-immune-inflammation index" OR "Systemic immune-inflammation index" OR "neutrophil×platelets/lymphocyte" ) )


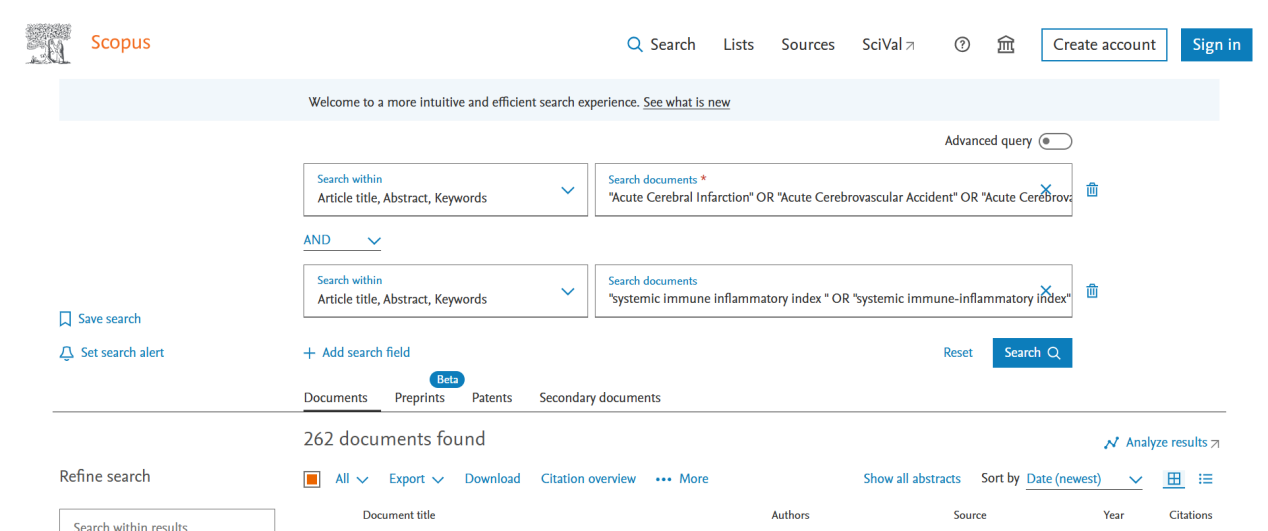


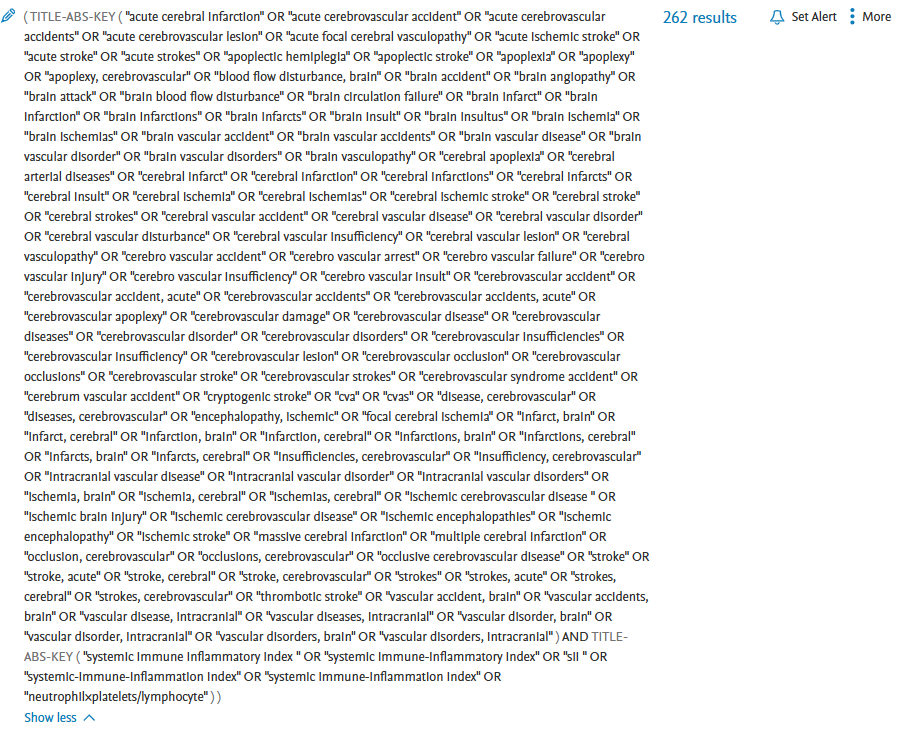


# OVID:193

1 Cerebral Infarction/

2 Stroke/

3 Brain Infarction/

4 Cerebrovascular Disorders/

5 Brain Ischemia/

6 Cerebral Arterial Diseases/

7 (Acute Cerebral Infarction or Acute Cerebrovascular Accident or Acute Cerebrovascular Accidents or Acute Cerebrovascular Lesion or Acute Focal Cerebral Vasculopathy or Acute Ischemic Stroke or Acute Stroke or Acute Strokes or Apoplectic Hemiplegia or Apoplectic Stroke or Apoplexia or Apoplexy or Apoplexy, Cerebrovascular or Blood Flow Disturbance, Brain or Brain Accident or Brain Angiopathy or Brain Attack or Brain Blood Flow Disturbance or Brain Circulation Failure or Brain Infarct or Brain Infarction or Brain Infarctions or Brain Infarcts or Brain Insult or Brain Insultus or Brain Ischemia or Brain Ischemias or Brain Vascular Accident or Brain Vascular Accidents or Brain Vascular Disease or Brain Vascular Disorder or Brain Vascular Disorders or Brain Vasculopathy or Cerebral Apoplexia or Cerebral Arterial Diseases or Cerebral Infarct or Cerebral Infarction or Cerebral Infarctions or Cerebral Infarcts or Cerebral Insult or Cerebral Ischemia or Cerebral Ischemias or Cerebral Ischemic Stroke or Cerebral Stroke or Cerebral Strokes or Cerebral Vascular Accident or Cerebral Vascular Disease or Cerebral Vascular Disorder or Cerebral Vascular Disturbance or Cerebral Vascular Insufficiency or Cerebral Vascular Lesion or Cerebral Vasculopathy or Cerebro Vascular Accident or Cerebro Vascular Arrest or Cerebro Vascular Failure or Cerebro Vascular Injury or Cerebro Vascular Insufficiency or Cerebro Vascular Insult or Cerebrovascular Accident or Cerebrovascular Accident, Acute or Cerebrovascular Accidents or Cerebrovascular Accidents, Acute or Cerebrovascular Apoplexy or Cerebrovascular Damage or Cerebrovascular Disease or Cerebrovascular Diseases or Cerebrovascular Disorder or Cerebrovascular Disorders or Cerebrovascular Insufficiencies or Cerebrovascular Insufficiency or Cerebrovascular Lesion or Cerebrovascular Occlusion or Cerebrovascular Occlusions or Cerebrovascular Stroke or Cerebrovascular Strokes or Cerebrovascular Syndrome Accident or Cerebrum Vascular Accident or Cryptogenic Stroke or CVA or CVAs or Disease, Cerebrovascular or Diseases, Cerebrovascular or Encephalopathy, Ischemic or Focal Cerebral Ischemia or Infarct, Brain or Infarct, Cerebral or Infarction, Brain or Infarction, Cerebral or Infarctions, Brain or Infarctions, Cerebral or Infarcts, Brain or Infarcts, Cerebral or Insufficiencies, Cerebrovascular or Insufficiency, Cerebrovascular or Intracranial Vascular Disease or Intracranial Vascular Disorder or Intracranial Vascular Disorders or Ischemia, Brain or Ischemia, Cerebral or Ischemias, Cerebral or Ischemic Cerebrovascular Disease or Ischemic Brain Injury or Ischemic Cerebrovascular Disease or Ischemic Encephalopathies or Ischemic Encephalopathy or Ischemic Stroke or Massive Cerebral Infarction or Multiple Cerebral Infarction or Occlusion, Cerebrovascular or Occlusions, Cerebrovascular or Occlusive Cerebrovascular Disease or Stroke or Stroke, Acute or Stroke, Cerebral or Stroke, Cerebrovascular or Strokes or Strokes, Acute or Strokes, Cerebral or Strokes, Cerebrovascular or Thrombotic Stroke or Vascular Accident, Brain or Vascular Accidents, Brain or Vascular Disease, Intracranial or Vascular Diseases, Intracranial or Vascular Disorder, Brain or Vascular Disorder, Intracranial or Vascular Disorders, Brain or Vascular Disorders, Intracranial).mp. [mp=title, book title, abstract, original title, name of substance word, subject heading word, floating sub-heading word, keyword heading word, organism supplementary concept word, protocol supplementary concept word, rare disease supplementary concept word, unique identifier, synonyms, population supplementary concept word, anatomy supplementary concept word]

8 1 or 2 or 3 or 4 or 5 or 6 or 7

9 (systemic immune inflammatory index or systemic immune-inflammatory index or SII or Systemic-immune-inflammation index or Systemic immune-inflammation index Systemic immune-inflammation index).mp. [mp=title, book title, abstract, original title, name of substance word, subject heading word, floating sub-heading word, keyword heading word, organism supplementary concept word, protocol supplementary concept word, rare disease supplementary concept word, unique identifier, synonyms, population supplementary concept word, anatomy supplementary concept word]

10 limit 9 to yr="1860 - 2024"

11 8 and 10


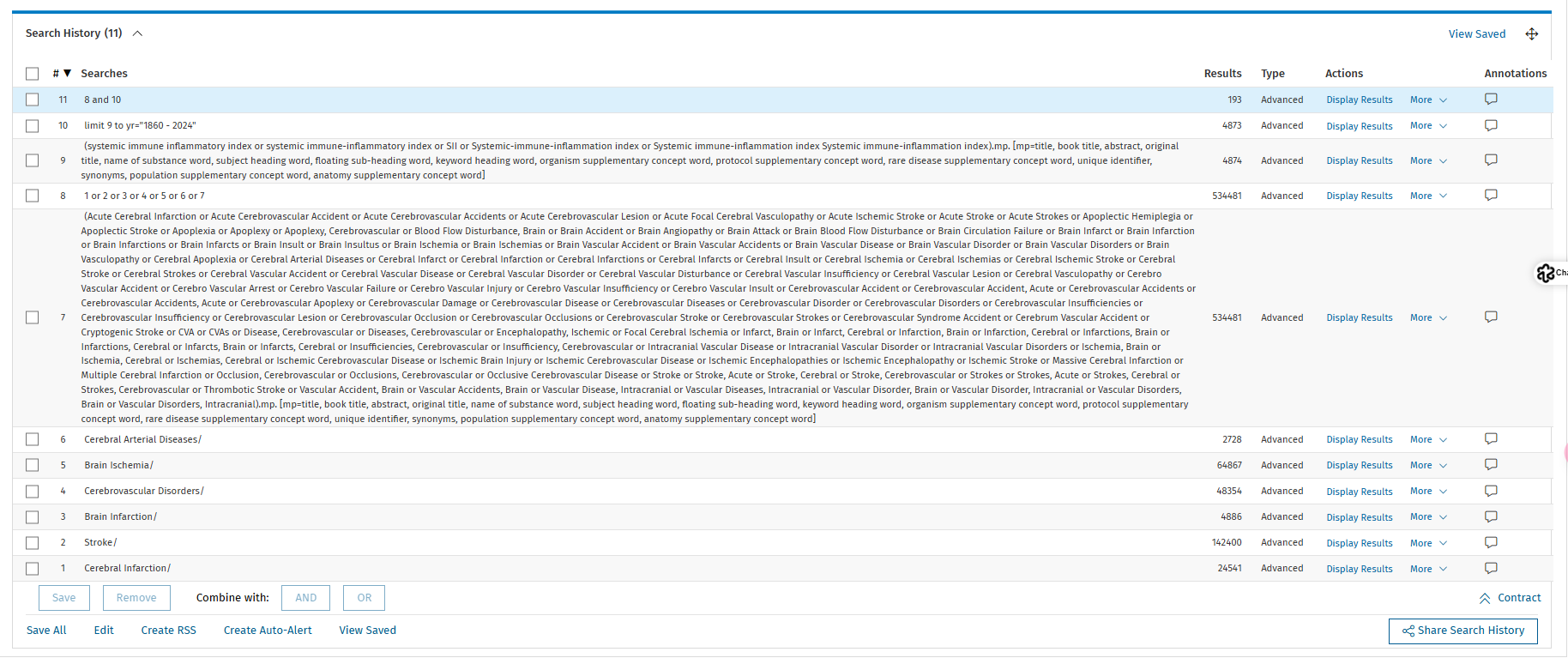


# CNKI:81

(SU='脑梗死'+'脑梗塞'+'卒中'+'缺血性卒中'+'缺血性脑卒中'+'脑卒中'+'脑栓塞'+'脑血栓'+'缺血性脑血管病'+'缺血性脑损伤'+'缺血性脑病'+'脑缺血'+'腔隙性梗塞'+'腔隙性脑梗死'+'腔隙性脑梗塞'+'大面积脑梗死'+'大面积脑梗'+'多发性脑梗死'+'脑梗死后遗症'+'脑卒中后遗症'+'脑血栓后遗症'+'脑干梗死'+'小脑梗死'+‘丘脑梗死’+‘缺血半暗带’+'中风'+'中风病'+'脑中风'+'缺血性中风'+'中风偏瘫'+'脑血管病'+'脑血管病后遗症'+'中风后遗症'+'脑血管意外'+'脑血管疾病'+'神经功能缺损'+’急性脑梗死’) AND (SU='全身免疫炎症指数'+'全身免疫-炎症指数'+'全身炎症免疫指数'+'全身炎症-免疫指数'+'系统免疫炎症指数 '+'系统免疫-炎症指数'+'系统性炎症指数'+'系统性免疫炎症指数'+'血小板计数x中性粒细胞计数/淋巴细胞计数'+'systemic immune inflammatory index'+'systemic immune-inflammatory index'+'SII'+'Systemic-immune-inflammation index'+'Systemic immune-inflammation index'+'neutrophil ×platelets/lymphocyte')


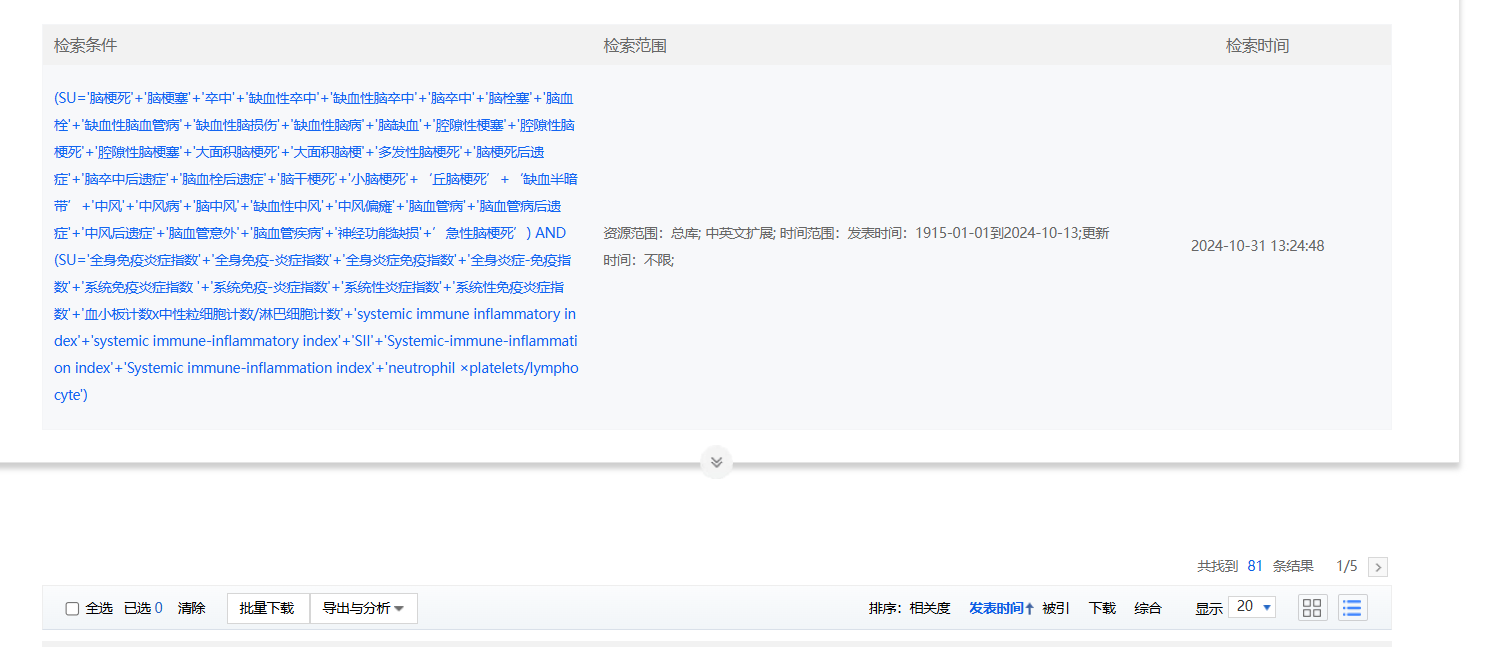


# Wanfang: 115

((主题:“脑梗死”OR“脑梗塞”OR“卒中”OR“缺血性卒中“OR”缺血性脑卒中“OR”脑卒中“OR”脑栓塞“OR”脑血栓“OR”缺血性脑血管病“OR”缺血性脑损伤”OR”缺血性脑病”OR”脑缺血”OR”腔隙性梗塞”OR”腔隙性脑梗死”OR”腔隙性脑梗塞”OR”大面积脑梗死”OR”大面积脑梗”OR”多发性脑梗死”OR”脑梗死后遗症”OR”脑卒中后遗症”OR”脑血栓后遗症”OR”脑干梗死”OR”小脑梗死”OR”丘脑梗死”OR”缺血半暗带”OR”中风”OR”中风病”OR”脑中风”OR”缺血性中风”OR”中风偏瘫”OR”脑血管病”OR”脑血管病后遗症”OR”中风后遗症”OR”脑血管意外”OR”脑血管疾病”OR”神经功能缺损”OR”急性脑梗死” ) AND (主题: “全身免疫炎症指数”OR”全身免疫-炎症指数”OR”全身炎症免疫指数”OR”全身炎症-免疫指数”OR”系统免疫炎症指数”OR”系统免疫-炎症指数”OR”系统性炎症指数”OR”系统性免疫炎症指数”OR”血小板计数x中性粒细胞计数/淋巴细胞计数”OR“systemic immune inflammatory index”OR“systemic immune-inflammatory index”OR“SII“OR”Systemic-immune-inflammation index“OR”Systemic immune-inflammation index“OR”neutrophil ×platelets/lymphocyte“)) and 发表时间:*-2024


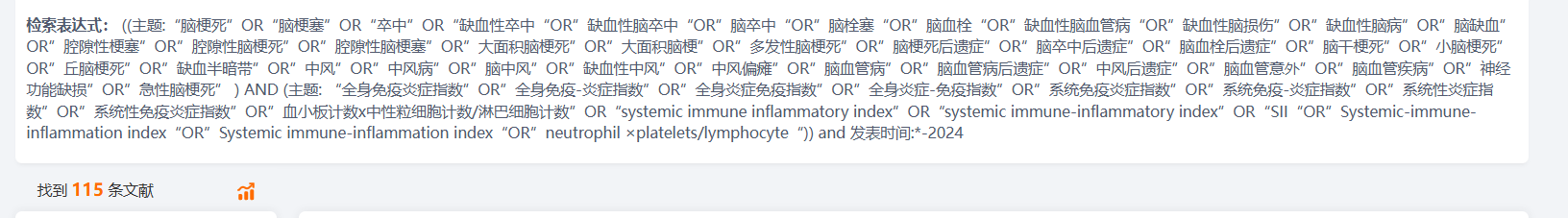


**VIP: 0**

(U=全身免疫炎症指数 OR 全身免疫-炎症指数 OR 全身炎症免疫指数 OR 全身炎症-免疫指数 OR 系统免疫炎症指数 OR 系统免疫-炎症指数 OR 系统性炎症指数 OR 系统性免疫炎症指数 OR 血小板计数x中性粒细胞计数/淋巴细胞计数 OR systemic immune inflammatory index OR systemic immune-inflammatory index OR SII OR Systemic-immune-inflammation index OR Systemic immune-inflammation index OR neutrophil×platelets/lymphocyte ) AND (U=脑梗死 OR 脑梗塞 OR 卒中 OR 缺血性卒中 OR 缺血性脑卒中 OR 脑卒中 OR 脑栓塞 OR 脑血栓 OR 缺血性脑血管病 OR 缺血性脑损伤 OR 缺血性脑病 OR 脑缺血 OR 腔隙性梗塞 OR 腔隙性脑梗死 OR 腔隙性脑梗塞 OR 大面积脑梗死 OR 大面积脑梗 OR 多发性脑梗死 OR 脑梗死后遗症 OR 脑卒中后遗症 OR 脑血栓后遗症 OR 脑干梗死 OR 小脑梗死 OR 丘脑梗死 OR 缺血半暗带 OR 中风 OR 中风病 OR 脑中风 OR 缺血性中风 OR 中风偏瘫 OR 脑血管病 OR 脑血管病后遗症 OR 中风后遗症 OR 脑血管意外 OR 脑血管疾病 OR 神经功能缺损 OR 急性脑梗死)


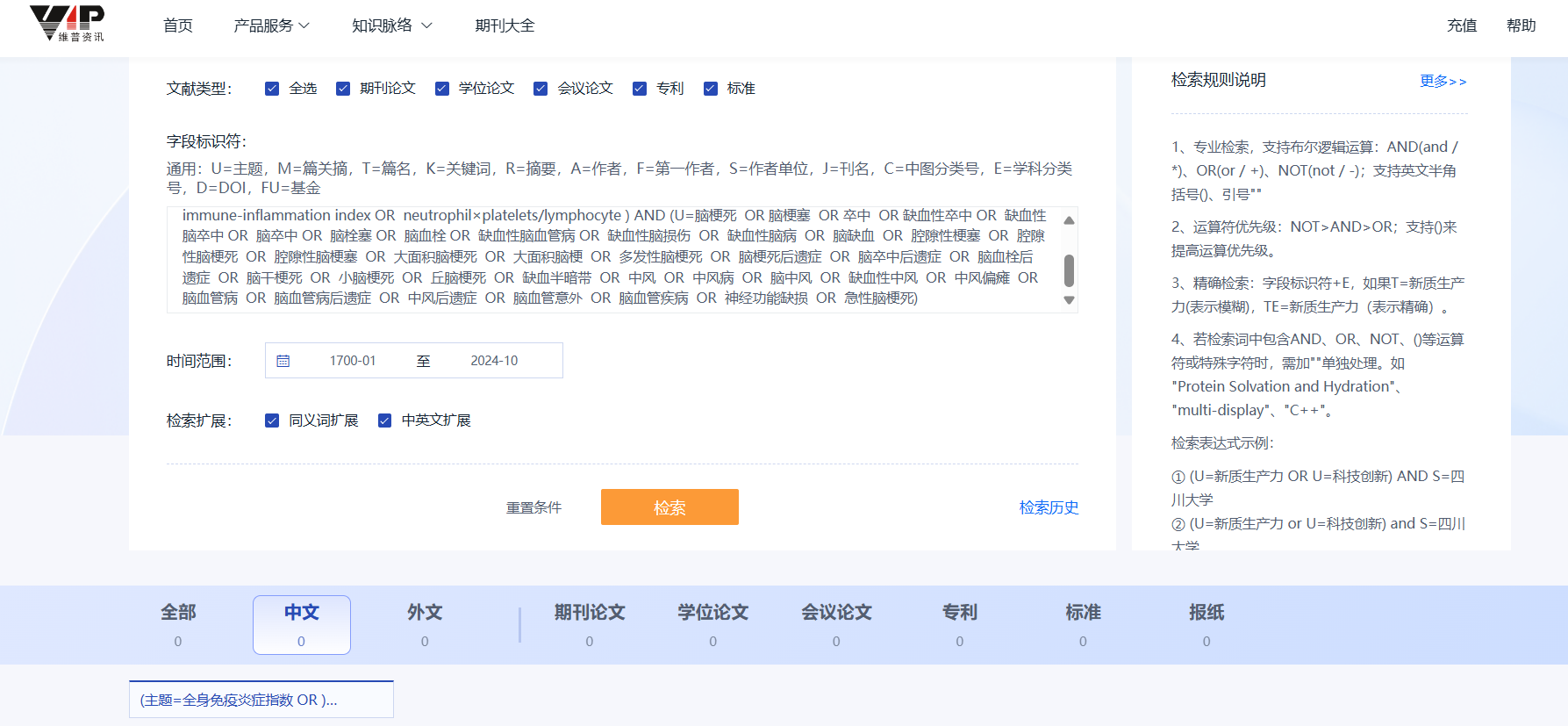


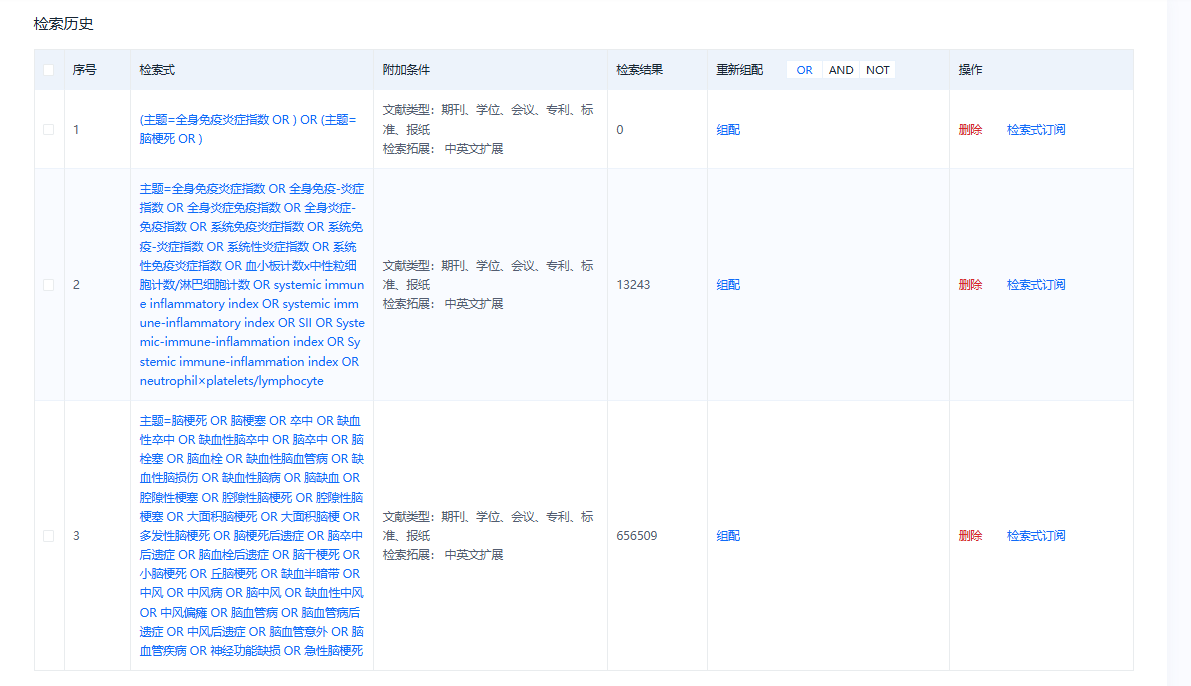


# CBM(Sinomed):75

1 ((全身免疫炎症指数 OR 全身免疫-炎症指数 OR 全身炎症免疫指数 OR 全身炎症-免疫指数 OR 系统免疫炎症指数 OR 系统免疫-炎症指数 OR 系统性炎症指数 OR 系统性免疫炎症指数 OR 血小板计数x中性粒细胞计数/淋巴细胞计数 OR systemic immune inflammatory index OR systemic immune-inflammatory index OR SII OR Systemic-immune-inflammation index OR Systemic immune-inflammation index OR neutrophil×platelets/lymphocyte) AND 1900-2024[日期]) AND (脑梗死 OR 脑梗塞 OR 卒中 OR 缺血性卒中 OR 缺血性脑卒中 OR 脑卒中 OR 脑栓塞 OR 脑血栓 OR 缺血性脑血管病 OR 缺血性脑损伤 OR 缺血性脑病 OR 脑缺血 OR 腔隙性梗塞 OR 腔隙性脑梗死 OR 腔隙性脑梗塞 OR 大面积脑梗死 OR 大面积脑梗 OR 多发性脑梗死 OR 脑梗死后遗症 OR 脑卒中后遗症 OR 脑血栓后遗症 OR 脑干梗死 OR 小脑梗死 OR 丘脑梗死 OR 缺血半暗带 OR 中风 OR 中风病 OR 脑中风 OR 缺血性中风 OR 中风偏瘫 OR 脑血管病 OR 脑血管病后遗症 OR 中风后遗症 OR 脑血管意外 OR 脑血管疾病 OR 神经功能缺损 OR 急性脑梗死 OR 脑血管障碍 OR 缺氧缺血, 脑)

2 (((("脑梗死"[不加权:扩展]) OR "脑血管障碍"[不加权:扩展]) OR "脑缺血"[不加权:扩展]) OR "卒中"[不加权:扩展]) OR "缺血性卒中"[不加权:扩展]

3 全身免疫炎症指数 OR 全身免疫-炎症指数 OR 全身炎症免疫指数 OR 全身炎症-免疫指数 OR 系统免疫炎症指数 OR 系统免疫-炎症指数 OR 系统性炎症指数 OR 系统性免疫炎症指数 OR 血小板计数x中性粒细胞计数/淋巴细胞计数 OR systemic immune inflammatory index OR systemic immune-inflammatory index OR SII OR Systemic-immune-inflammation index OR Systemic immune-inflammation index Systemic immune-inflammation index OR neutrophil×platelets/lymphocyte

4 (#3) AND (#2)

5 ((#4) OR (#1)) AND 1900-2024[日期]


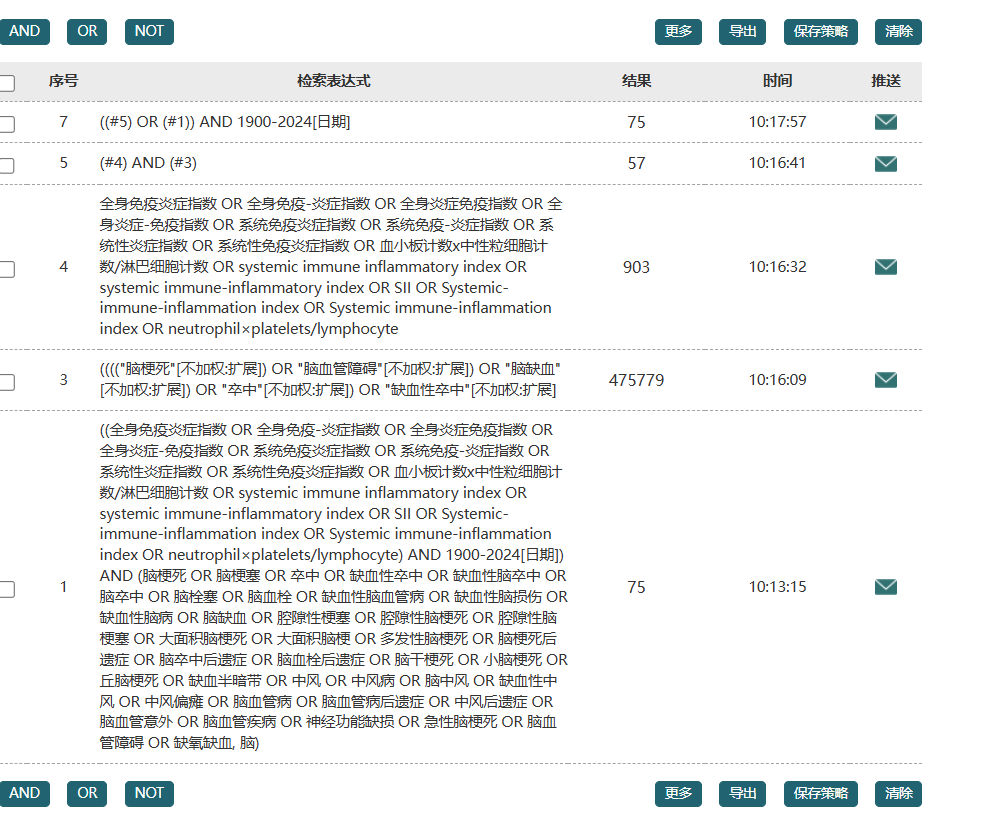


# ClinicalTrials.gov:19

**Acute Cerebral Infarction OR Acute Cerebrovascular Accident OR Acute Cerebrovascular Accidents OR Acute Cerebrovascular Lesion OR Acute Focal Cerebral Vasculopathy OR Acute Ischemic Stroke OR Acute Stroke OR Acute Strokes OR Apoplectic Hemiplegia OR Apoplectic Stroke OR Apoplexia OR Apoplexy OR Apoplexy, Cerebrovascular OR Blood Flow Disturbance, Brain OR Brain Accident OR Brain Angiopathy OR Brain Attack OR Brain Blood Flow Disturbance OR Brain Circulation Failure OR Brain Infarct OR Brain Infarction OR Brain Infarctions OR Brain Infarcts OR Brain Insult OR Brain Insultus OR Brain Ischemia OR Brain Ischemias OR Brain Vascular Accident OR Brain Vascular Accidents OR Brain Vascular Disease OR Brain Vascular Disorder OR Brain Vascular Disorders OR Brain Vasculopathy OR Cerebral Apoplexia OR Cerebral Arterial Diseases OR Cerebral Infarct OR Cerebral Infarction OR Cerebral Infarctions OR Cerebral Infarcts OR Cerebral Insult OR Cerebral Ischemia OR Cerebral Ischemias OR Cerebral Ischemic Stroke OR Cerebral Stroke OR Cerebral Strokes OR Cerebral Vascular Accident OR Cerebral Vascular Disease OR Cerebral Vascular Disorder OR Cerebral Vascular Disturbance OR Cerebral Vascular Insufficiency OR Cerebral Vascular Lesion OR Cerebral Vasculopathy OR Cerebro Vascular Accident OR Cerebro Vascular Arrest OR Cerebro Vascular Failure OR Cerebro Vascular Injury OR Cerebro Vascular Insufficiency OR Cerebro Vascular Insult OR Cerebrovascular Accident OR Cerebrovascular Accident, Acute OR Cerebrovascular Accidents OR Cerebrovascular Accidents, Acute OR Cerebrovascular Apoplexy OR Cerebrovascular Damage OR Cerebrovascular Disease OR Cerebrovascular Diseases OR Cerebrovascular Disorder OR Cerebrovascular Disorders OR Cerebrovascular Insufficiencies OR Cerebrovascular Insufficiency OR Cerebrovascular Lesion OR Cerebrovascular Occlusion OR Cerebrovascular Occlusions OR Cerebrovascular Stroke OR Cerebrovascular Strokes OR Cerebrovascular Syndrome Accident OR Cerebrum Vascular Accident OR Cryptogenic Stroke OR CVA OR CVAs OR Disease, Cerebrovascular OR Diseases, Cerebrovascular OR Encephalopathy, Ischemic OR Focal Cerebral Ischemia OR Infarct, Brain OR Infarct, Cerebral OR Infarction, Brain OR Infarction, Cerebral OR Infarctions, Brain OR Infarctions, Cerebral OR Infarcts, Brain OR Infarcts, Cerebral OR Insufficiencies, Cerebrovascular OR Insufficiency, Cerebrovascular OR Intracranial Vascular Disease OR Intracranial Vascular Disorder OR Intracranial Vascular Disorders OR Ischemia, Brain OR Ischemia, Cerebral OR Ischemias, Cerebral OR Ischemic Cerebrovascular Disease OR Ischemic Brain Injury OR Ischemic Cerebrovascular Disease OR Ischemic Encephalopathies OR Ischemic Encephalopathy OR Ischemic Stroke OR Massive Cerebral Infarction OR Multiple Cerebral Infarction OR Occlusion, Cerebrovascular OR Occlusions, Cerebrovascular OR Occlusive Cerebrovascular Disease OR Stroke OR Stroke, Acute OR Stroke, Cerebral OR Stroke, Cerebrovascular OR Strokes OR Strokes, Acute OR Strokes, Cerebral OR Strokes, Cerebrovascular OR Thrombotic Stroke OR Vascular Accident, Brain OR Vascular Accidents, Brain OR Vascular Disease, Intracranial OR Vascular Diseases, Intracranial OR Vascular Disorder, Brain OR Vascular Disorder, Intracranial OR Vascular Disorders, Brain OR Vascular Disorders, Intracranial | Other terms: systemic immune inflammatory index OR systemic immune-inflammatory index OR SII OR Systemic-immune-inflammation index OR Systemic immune-inflammation index**


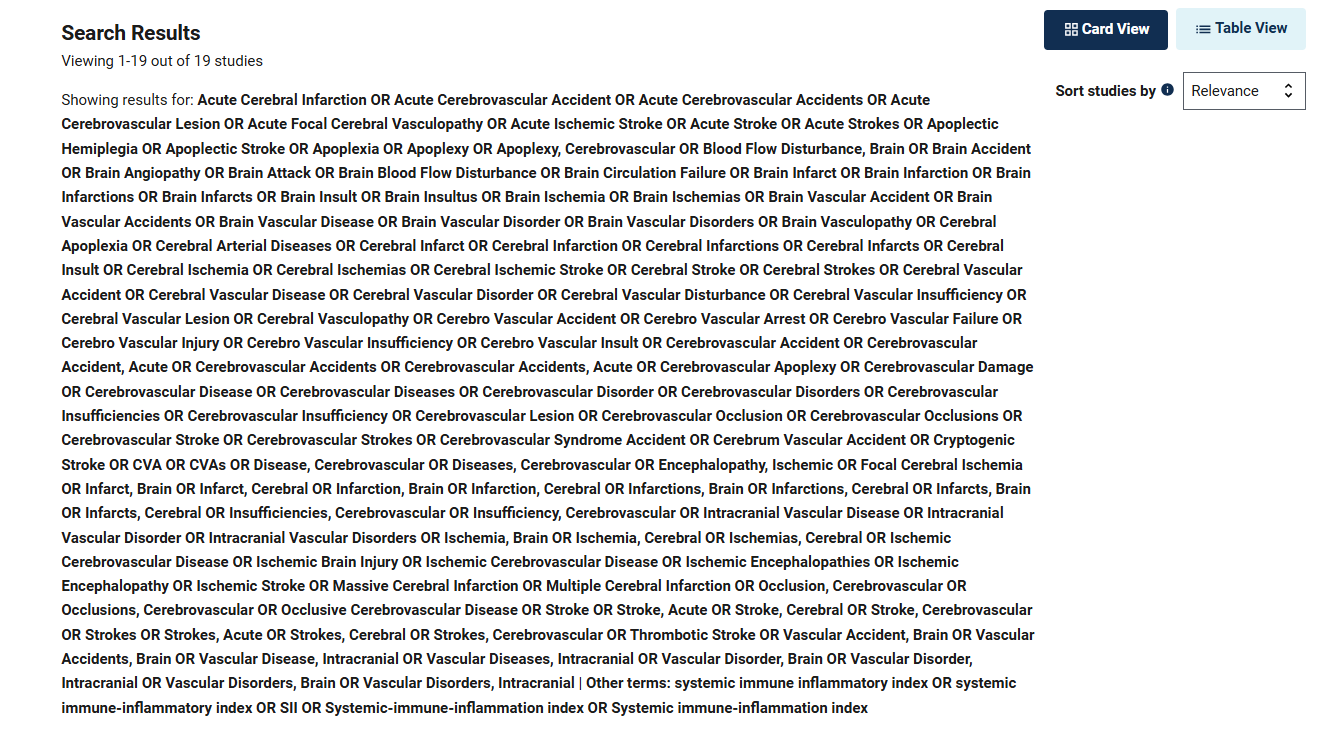


# WHO-ICTRP:0

1.Acute Cerebral Infarction OR Acute Cerebrovascular Accident OR Acute Cerebrovascular Accidents OR Acute Cerebrovascular Lesion OR Acute Focal Cerebral Vasculopathy OR Acute Ischemic Stroke OR Acute Stroke OR Acute Strokes OR Apoplectic Hemiplegia OR Apoplectic Stroke OR Apoplexia OR Apoplexy OR Apoplexy, Cerebrovascular OR Blood Flow Disturbance, Brain OR Brain Accident OR Brain Angiopathy OR Brain Attack OR Brain Blood Flow Disturbance OR Brain Circulation Failure OR Brain Infarct OR Brain Infarction OR Brain Infarctions OR Brain Infarcts OR Brain Insult OR Brain Insultus OR Brain Ischemia OR Brain Ischemias OR Brain Vascular Accident OR Brain Vascular Accidents OR Brain Vascular Disease OR Brain Vascular Disorder OR Brain Vascular Disorders OR Brain Vasculopathy OR Cerebral Apoplexia OR Cerebral Arterial Diseases OR Cerebral Infarct OR Cerebral Infarction OR Cerebral Infarctions OR Cerebral Infarcts OR Cerebral Insult OR Cerebral Ischemia OR Cerebral Ischemias OR Cerebral Ischemic Stroke OR Cerebral Stroke OR Cerebral Strokes OR Cerebral Vascular Accident OR Cerebral Vascular Disease OR Cerebral Vascular Disorder OR Cerebral Vascular Disturbance OR Cerebral Vascular Insufficiency OR Cerebral Vascular Lesion OR Cerebral Vasculopathy OR Cerebro Vascular Accident OR Cerebro Vascular Arrest OR Cerebro Vascular Failure OR Cerebro Vascular Injury OR Cerebro Vascular Insufficiency OR Cerebro Vascular Insult OR Cerebrovascular Accident OR Cerebrovascular Accident, Acute OR Cerebrovascular Accidents OR Cerebrovascular Accidents, Acute OR Cerebrovascular Apoplexy OR Cerebrovascular Damage OR Cerebrovascular Disease OR Cerebrovascular Diseases OR Cerebrovascular Disorder OR Cerebrovascular Disorders OR Cerebrovascular Insufficiencies OR Cerebrovascular Insufficiency OR Cerebrovascular Lesion OR Cerebrovascular Occlusion OR Cerebrovascular Occlusions OR Cerebrovascular Stroke OR Cerebrovascular Strokes OR Cerebrovascular Syndrome Accident OR Cerebrum Vascular Accident OR Cryptogenic Stroke OR CVA OR CVAs OR Disease, Cerebrovascular OR Diseases, Cerebrovascular OR Encephalopathy, Ischemic OR Focal Cerebral Ischemia OR Infarct, Brain OR Infarct, Cerebral OR Infarction, Brain OR Infarction, Cerebral OR Infarctions, Brain OR Infarctions, Cerebral OR Infarcts, Brain OR Infarcts, Cerebral OR Insufficiencies, Cerebrovascular OR Insufficiency, Cerebrovascular OR Intracranial Vascular Disease OR Intracranial Vascular Disorder OR Intracranial Vascular Disorders OR Ischemia, Brain OR Ischemia, Cerebral OR Ischemias, Cerebral OR Ischemic Cerebrovascular Disease OR Ischemic Brain Injury OR Ischemic Cerebrovascular Disease OR Ischemic Encephalopathies OR Ischemic Encephalopathy OR Ischemic Stroke OR Massive Cerebral Infarction OR Multiple Cerebral Infarction OR Occlusion, Cerebrovascular OR Occlusions, Cerebrovascular OR Occlusive Cerebrovascular Disease OR Stroke OR Stroke, Acute OR Stroke, Cerebral OR Stroke, Cerebrovascular OR Strokes OR Strokes, Acute OR Strokes, Cerebral OR Strokes, Cerebrovascular OR Thrombotic Stroke OR Vascular Accident, Brain OR Vascular Accidents, Brain OR Vascular Disease, Intracranial OR Vascular Diseases, Intracranial OR Vascular Disorder, Brain OR Vascular Disorder, Intracranial OR Vascular Disorders, Brain OR Vascular Disorders, Intracranial

AND

2. systemic immune inflammatory index OR systemic immune-inflammatory index OR SII OR Systemic-immune-inflammation index OR Systemic immune-inflammation index


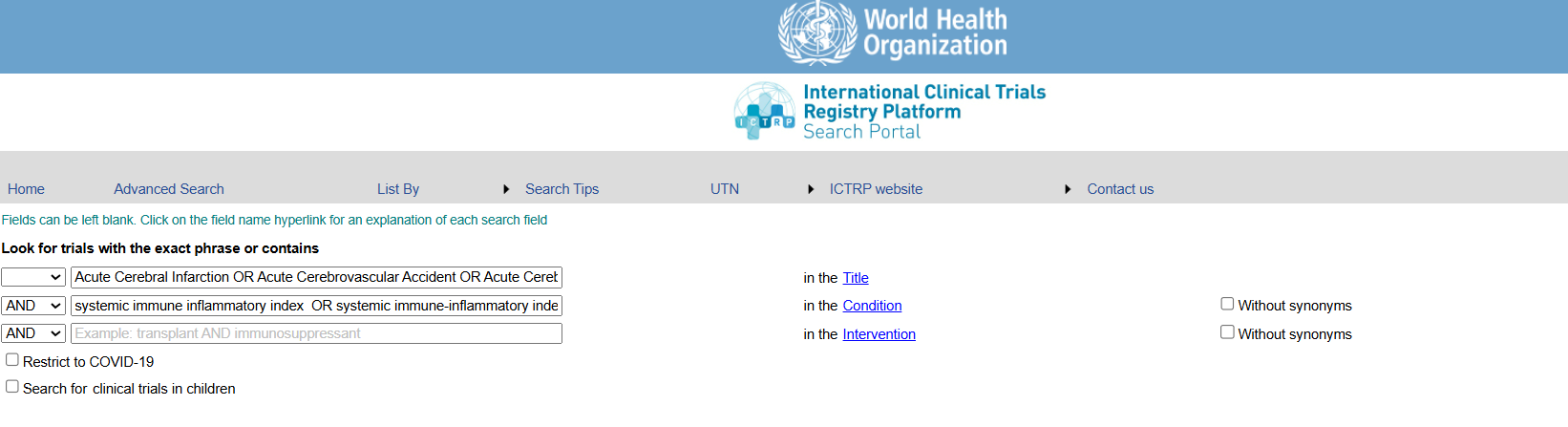


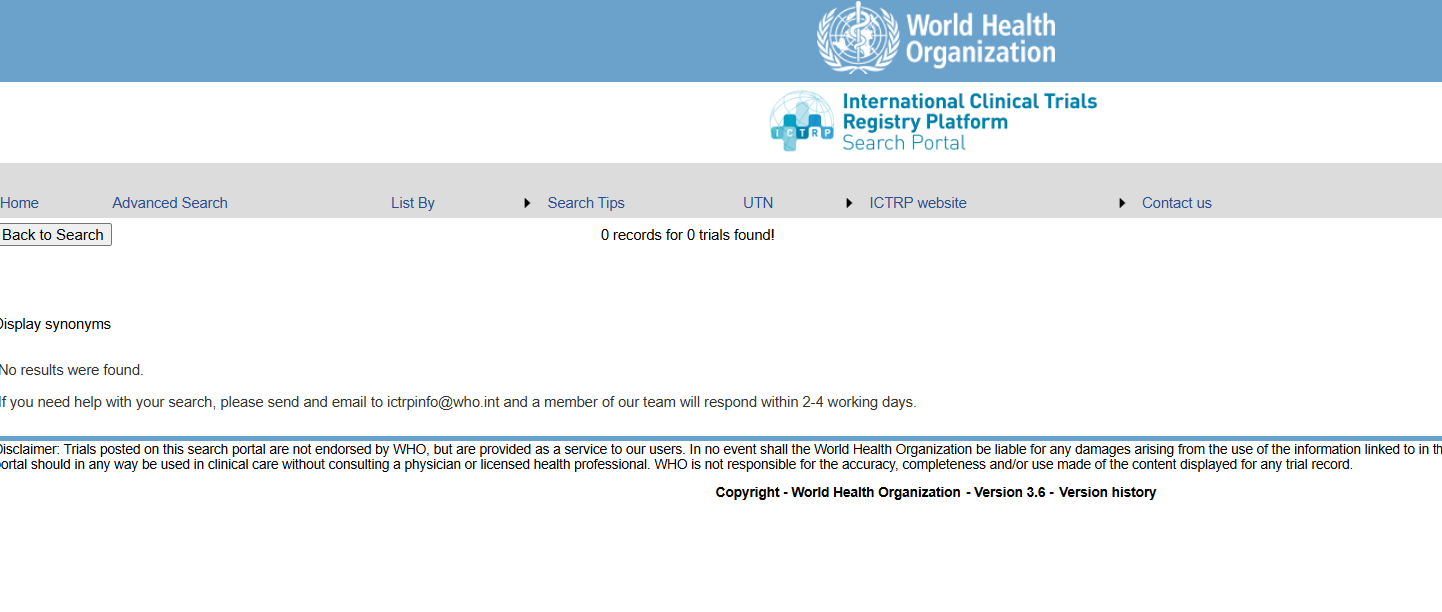


# ChiCTR:2


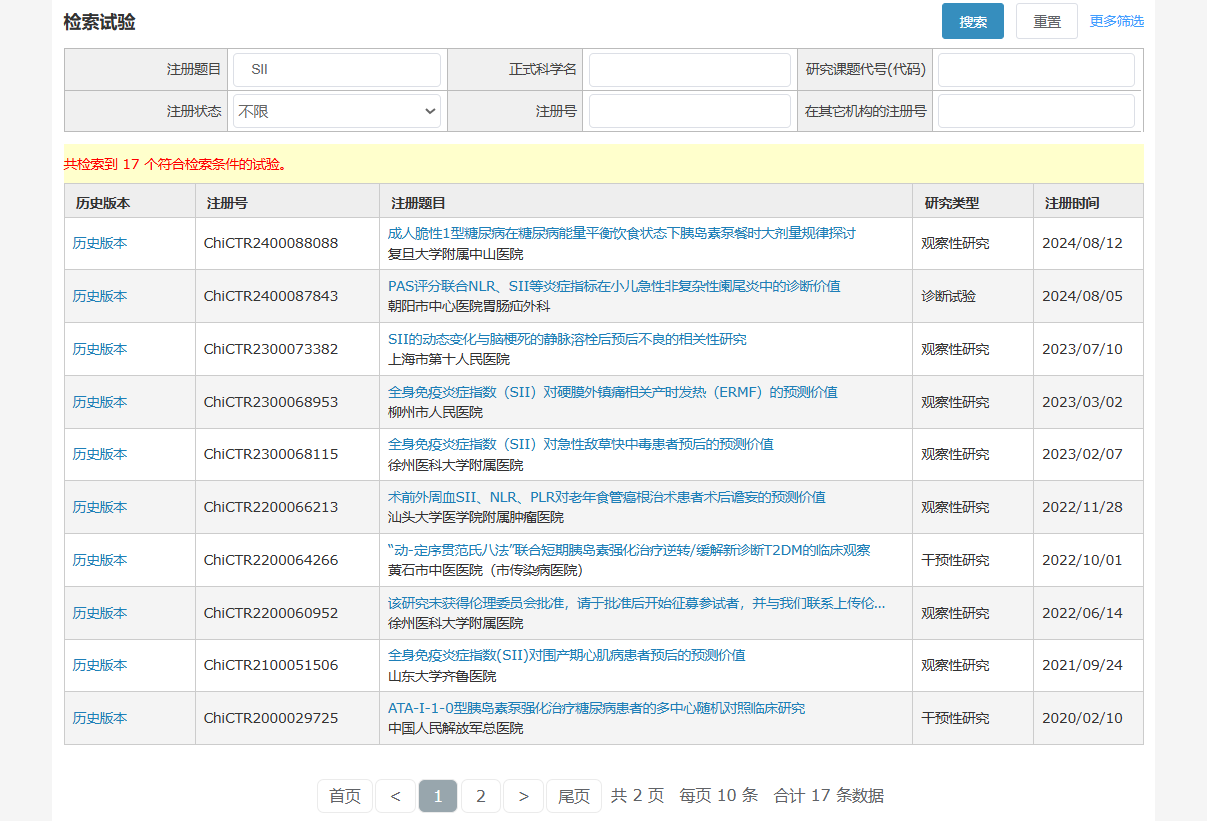


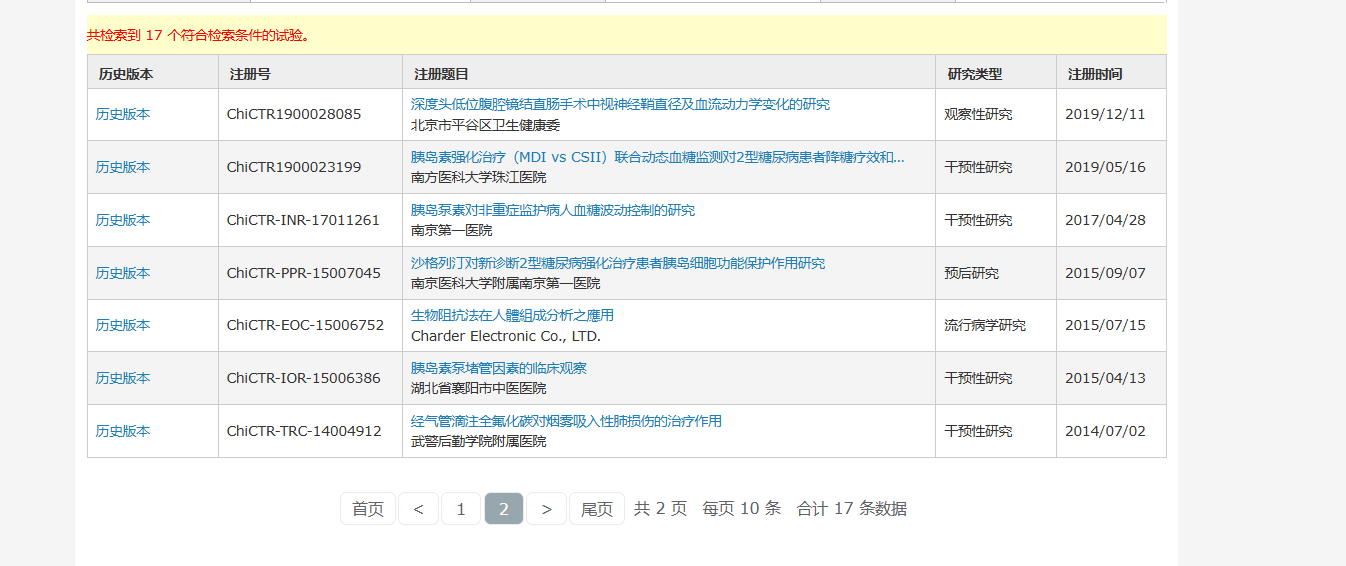


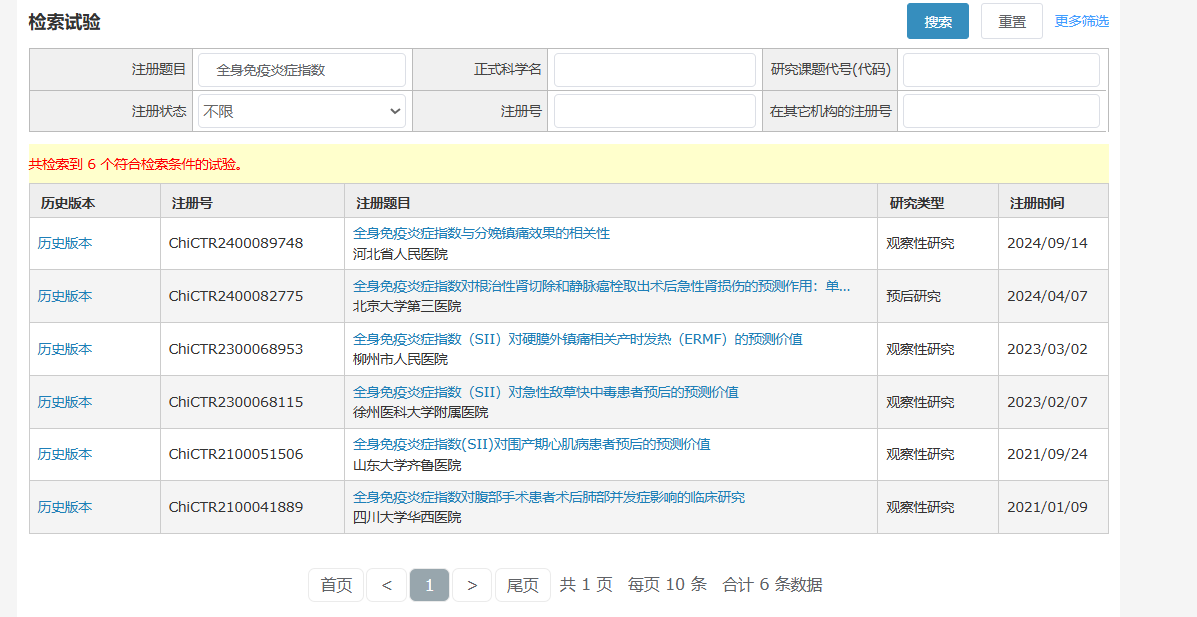


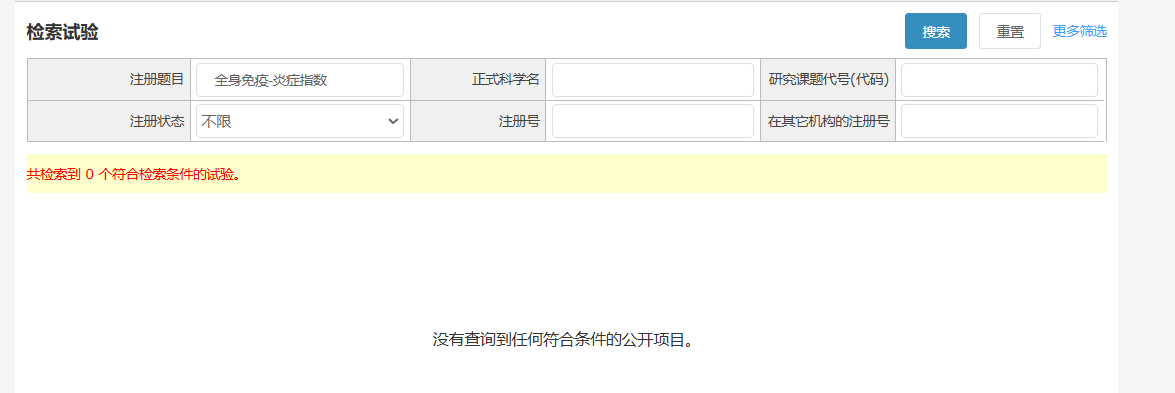


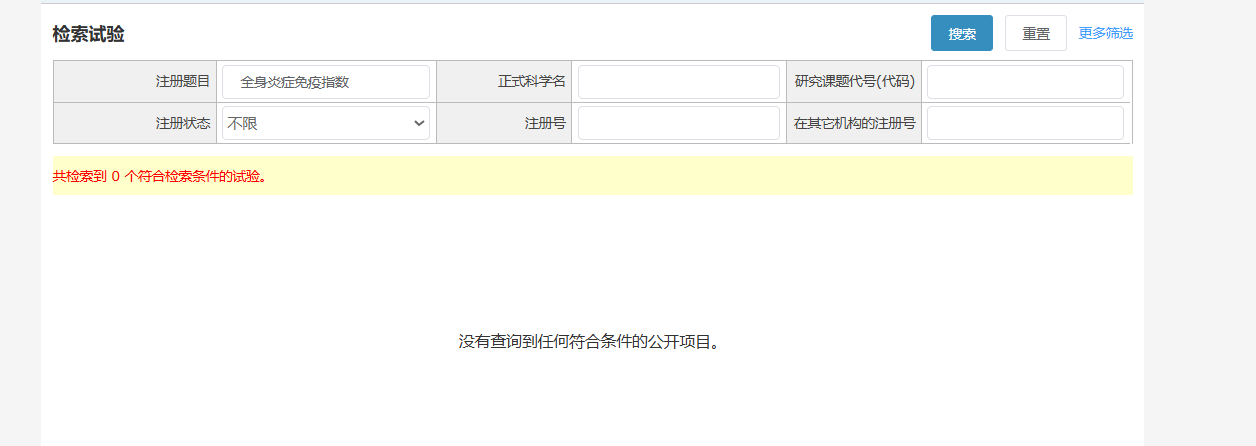


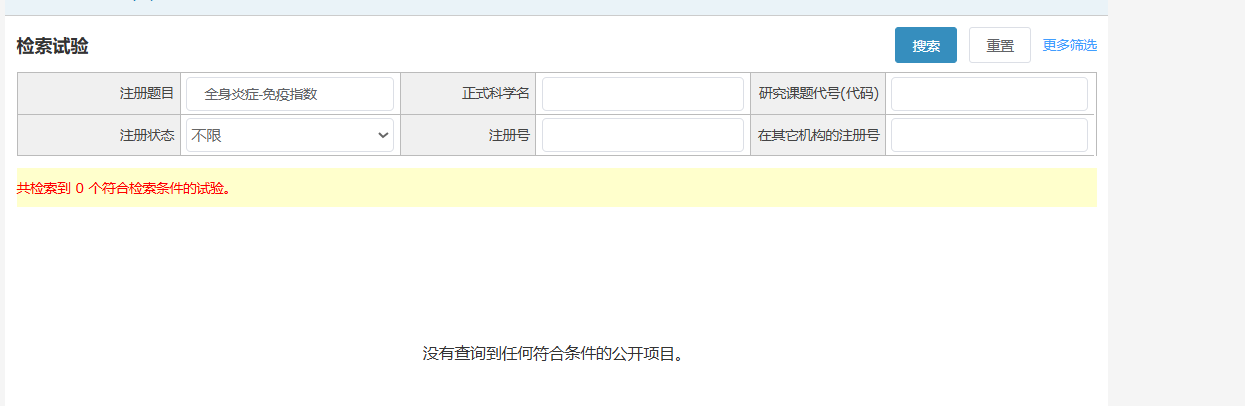


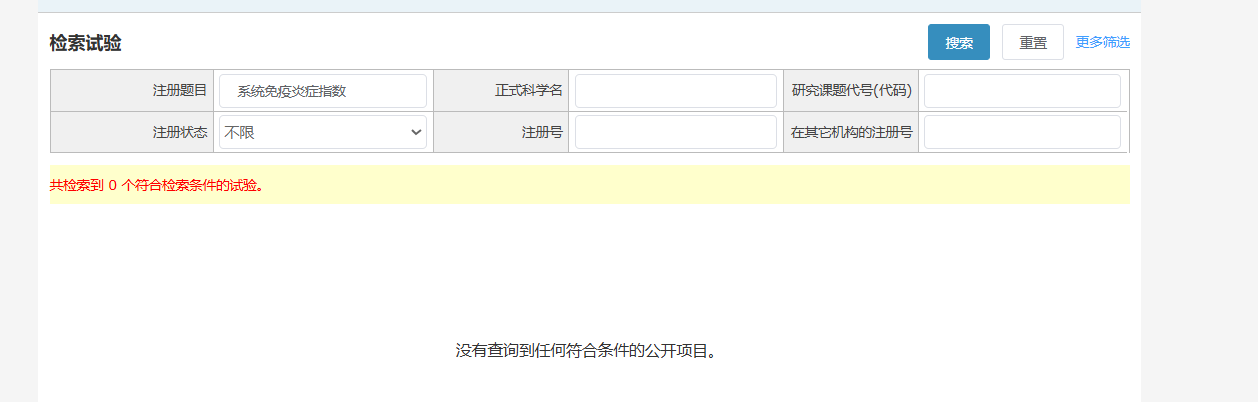


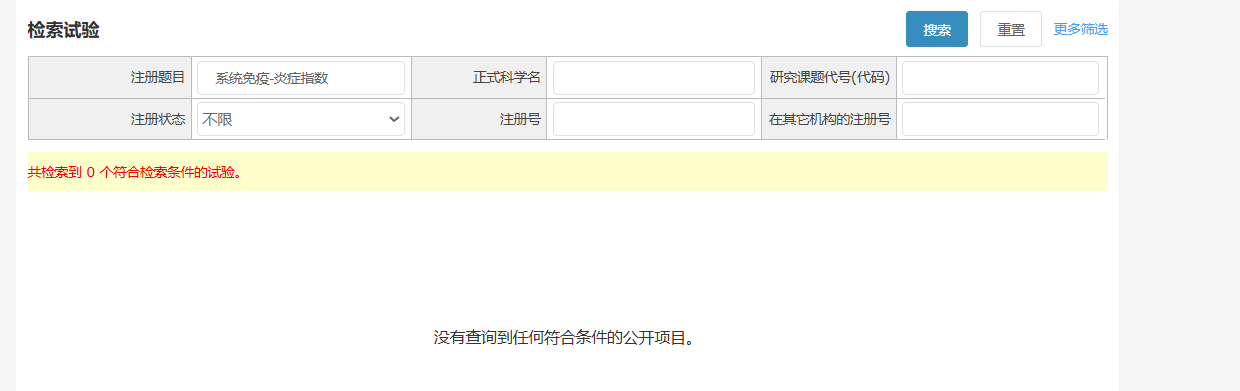


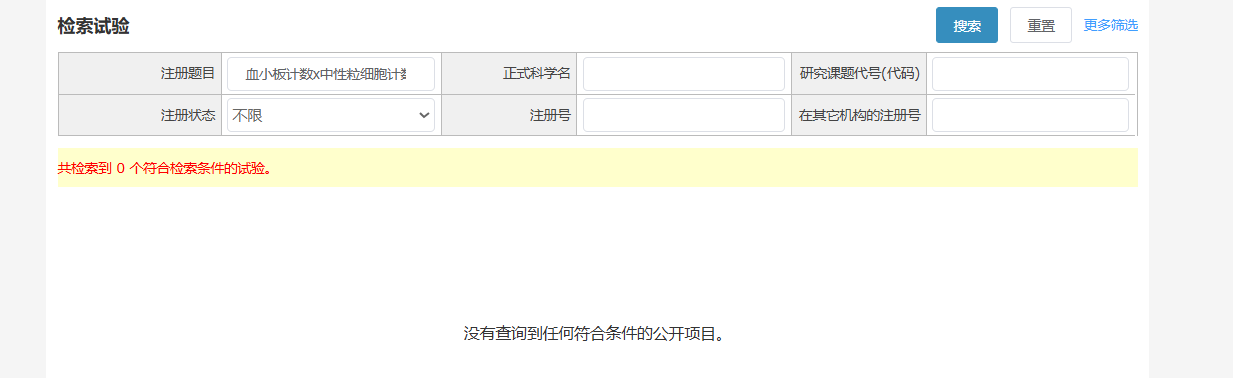

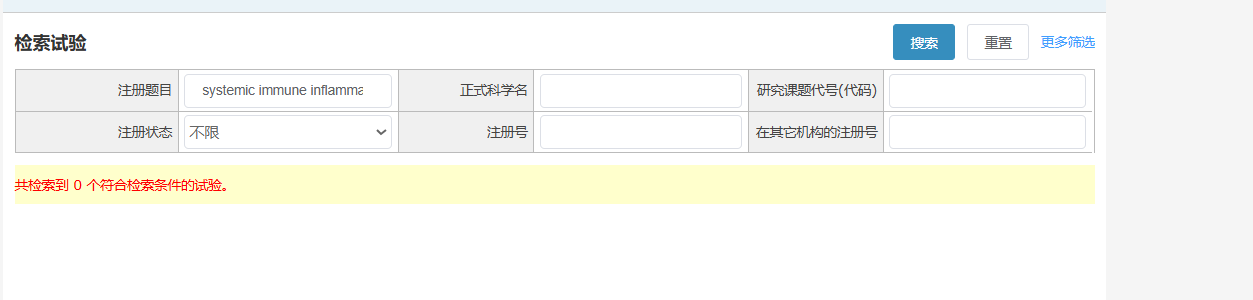


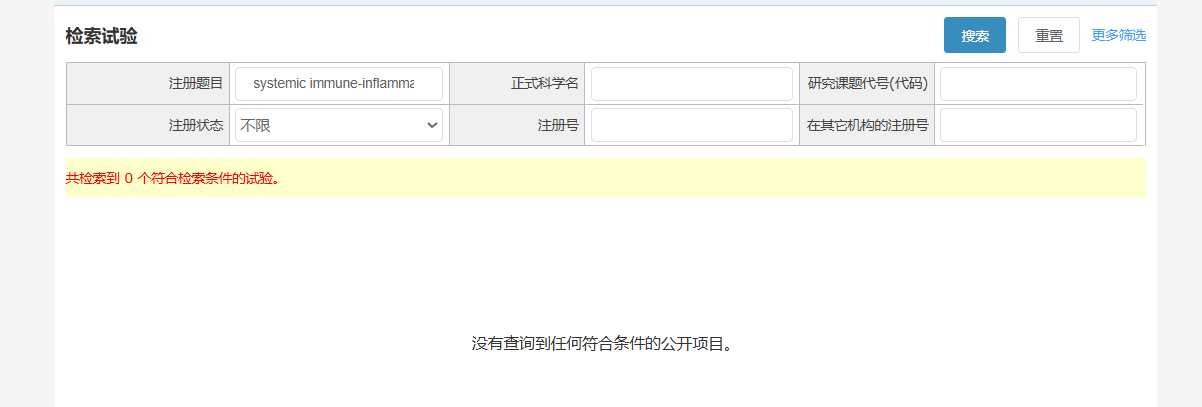


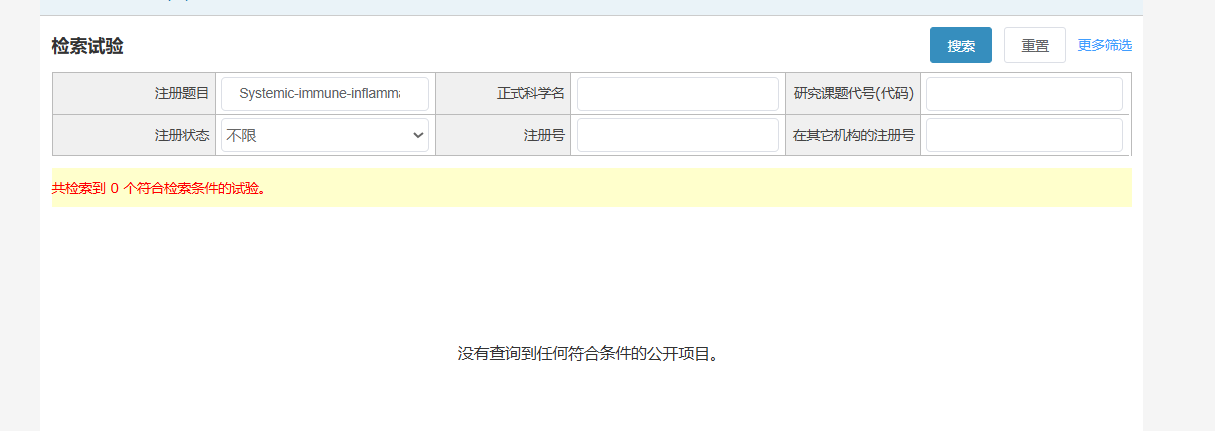


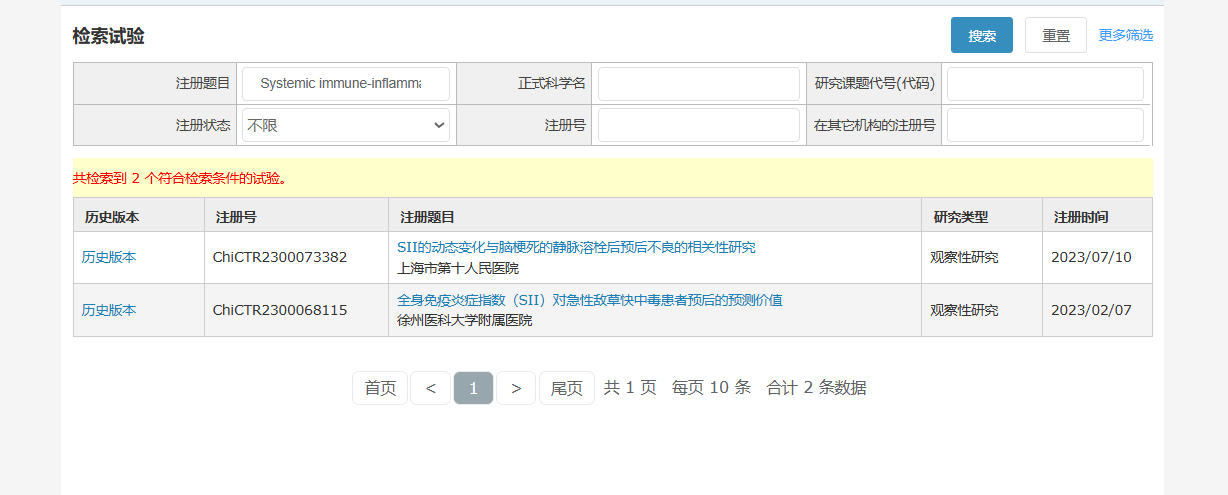


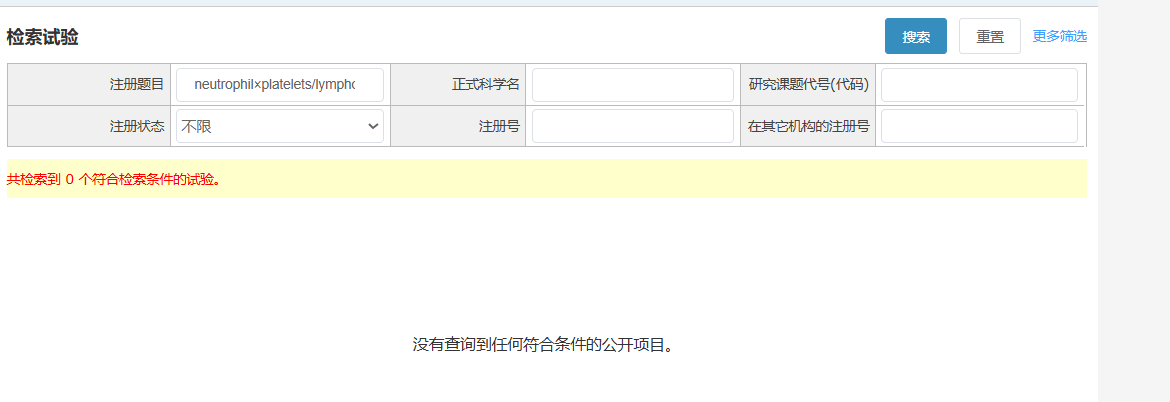


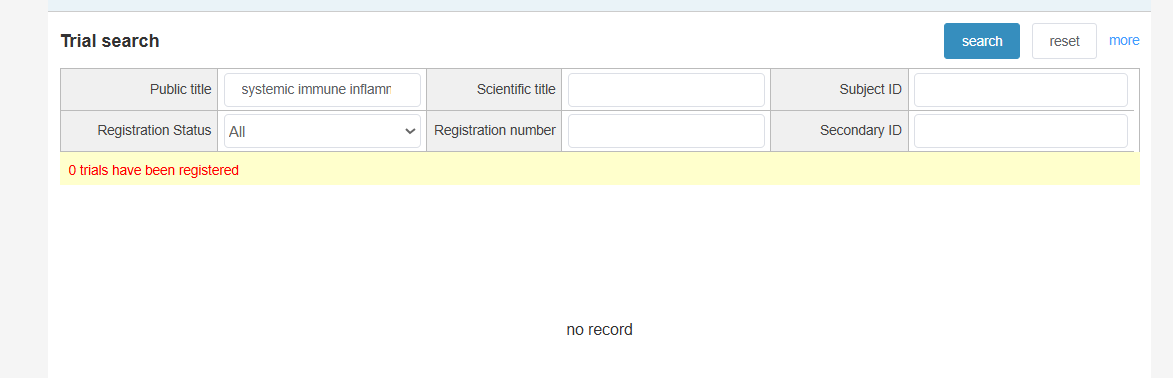


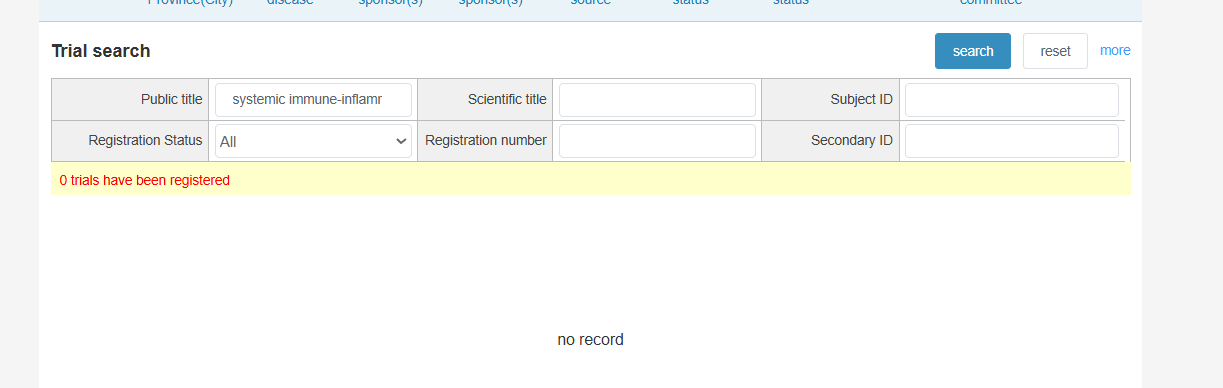


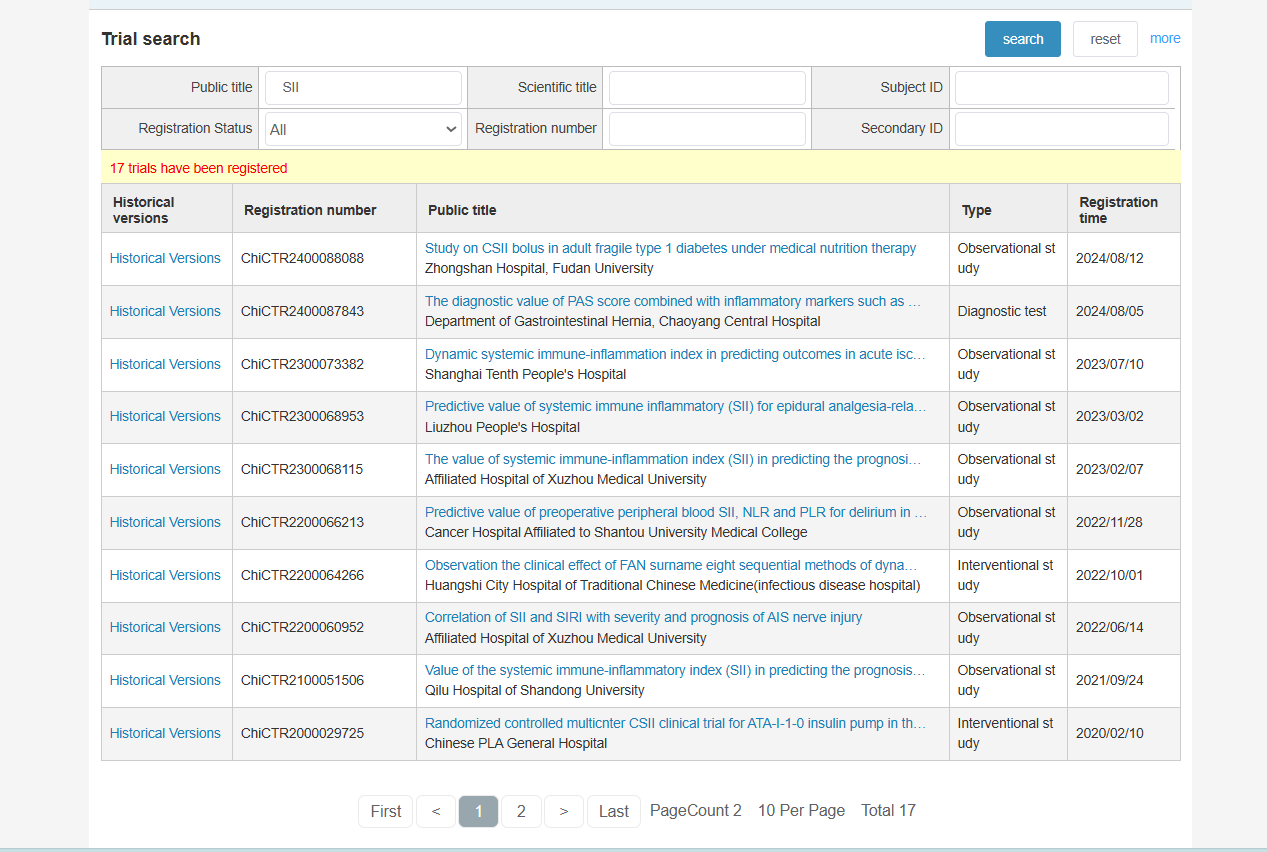


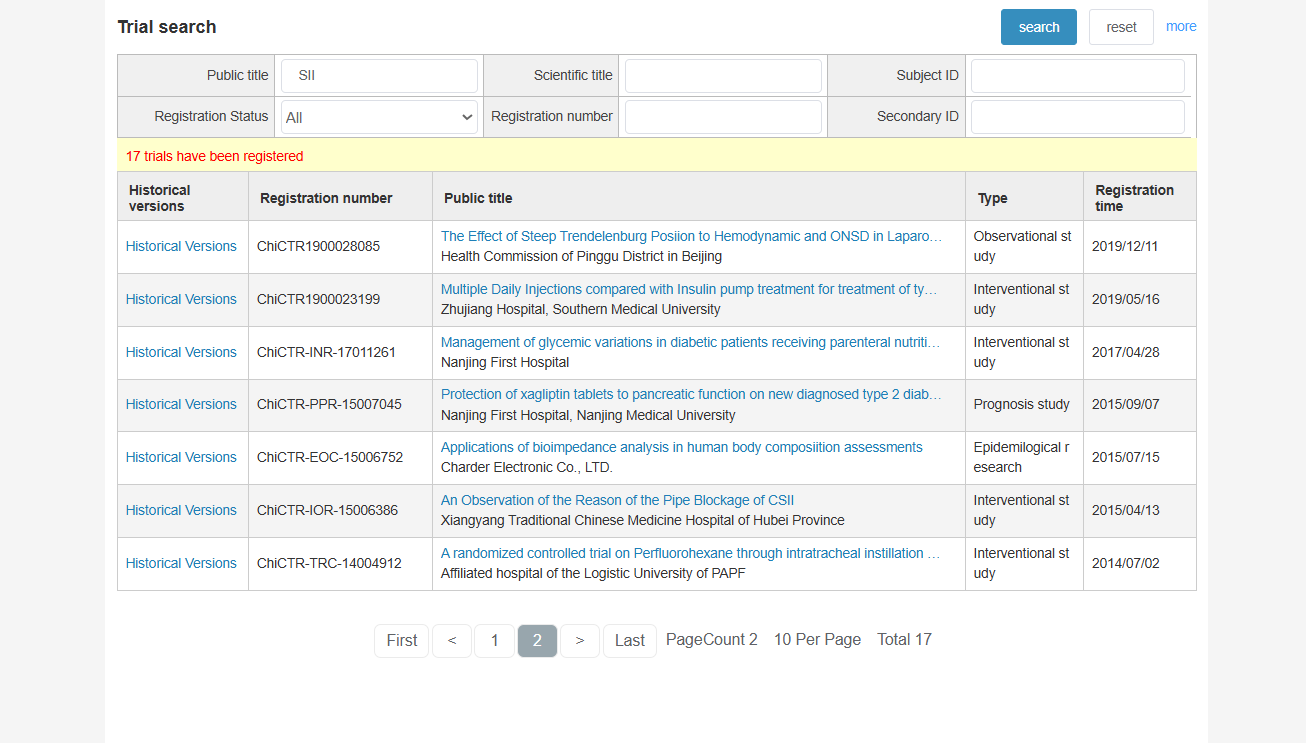


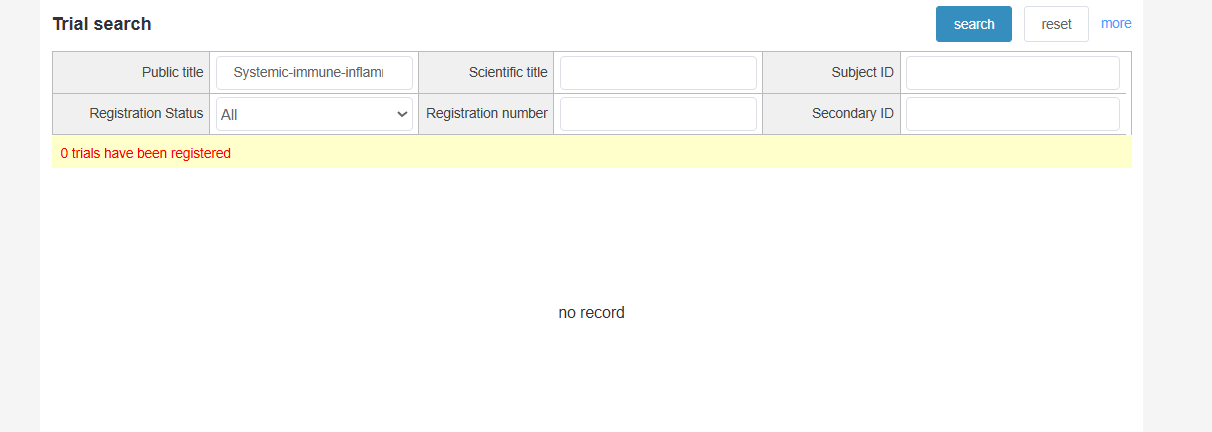


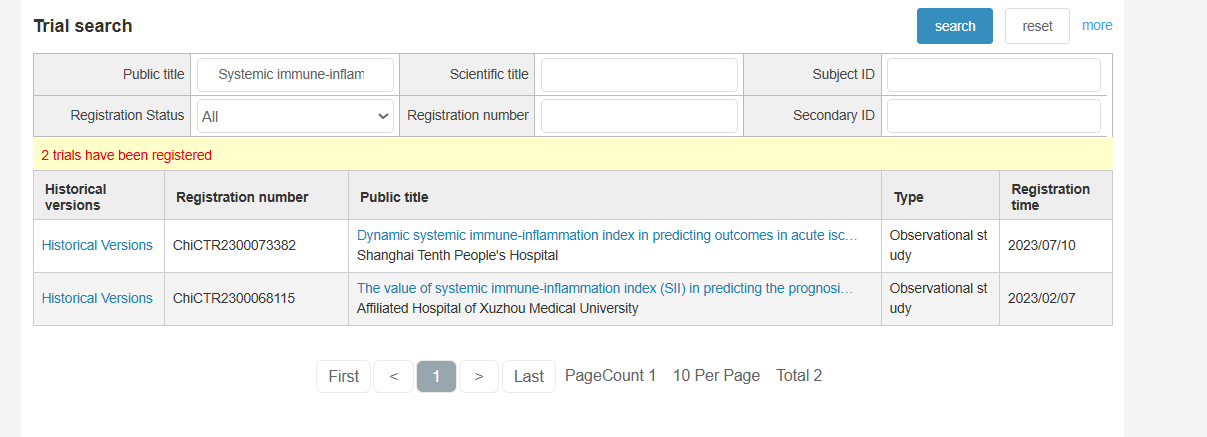


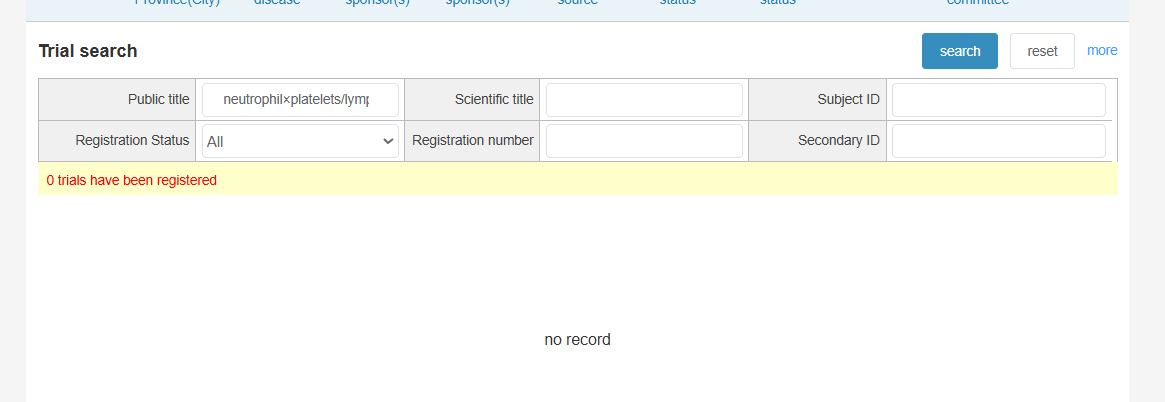


# DANSEASY(Greynet/OpenGrey/Opensigle): 0


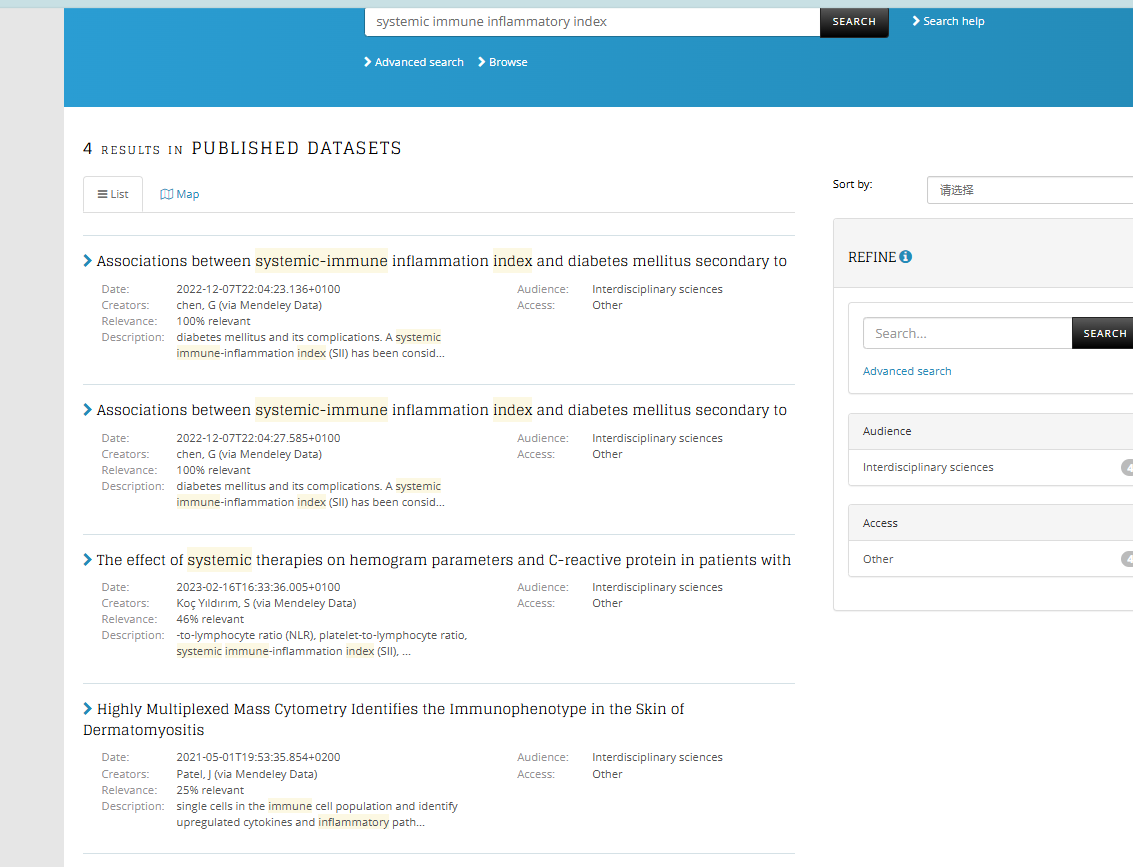


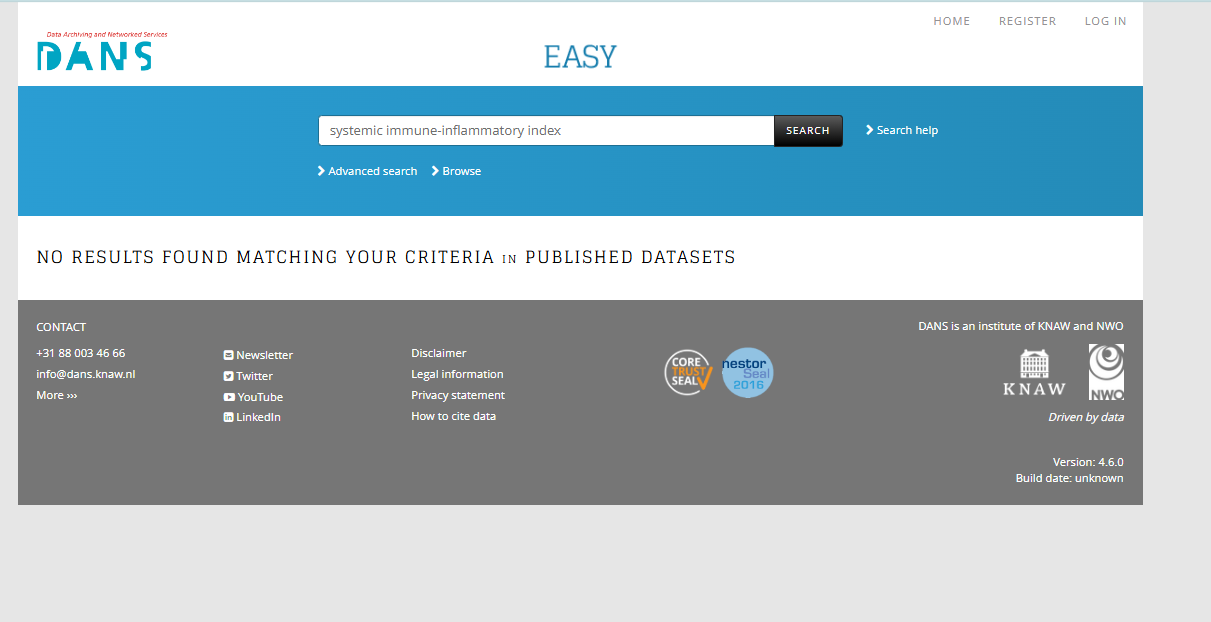


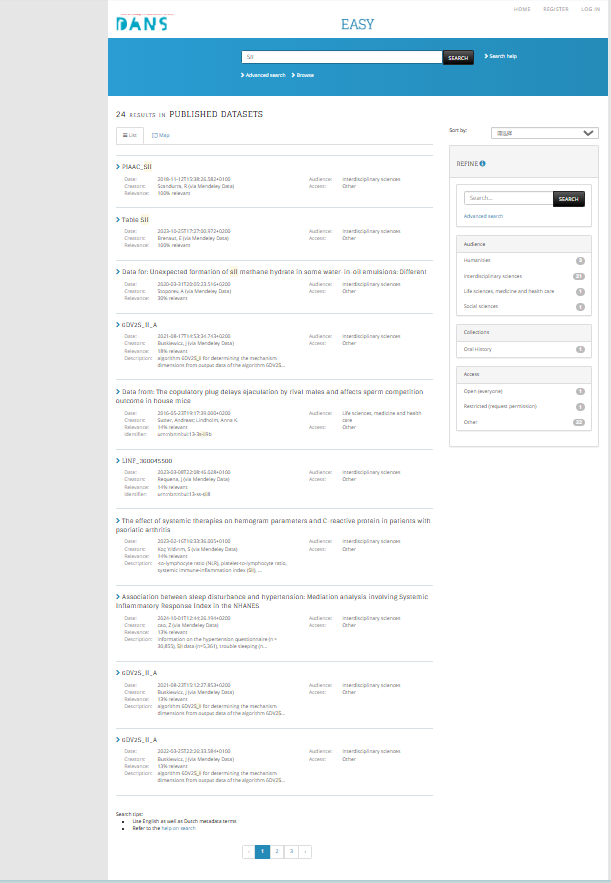

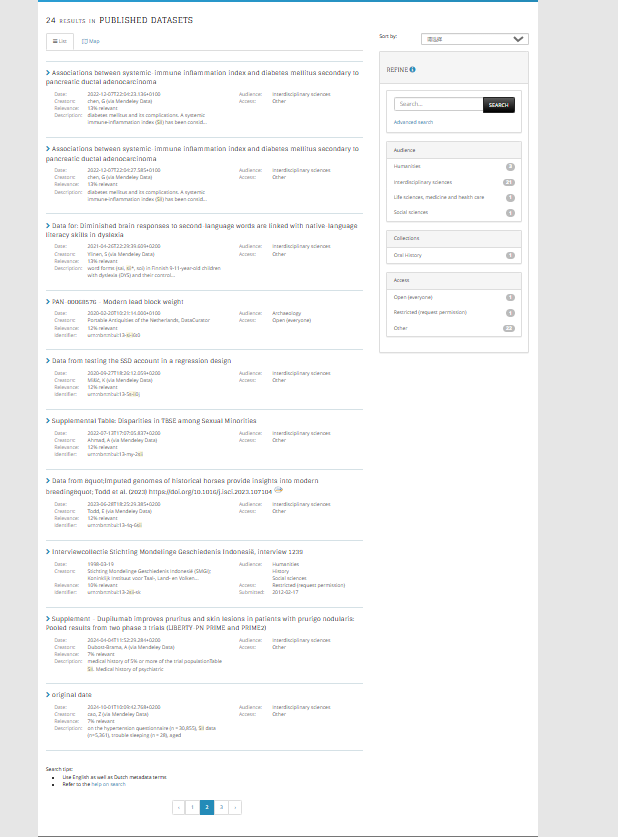

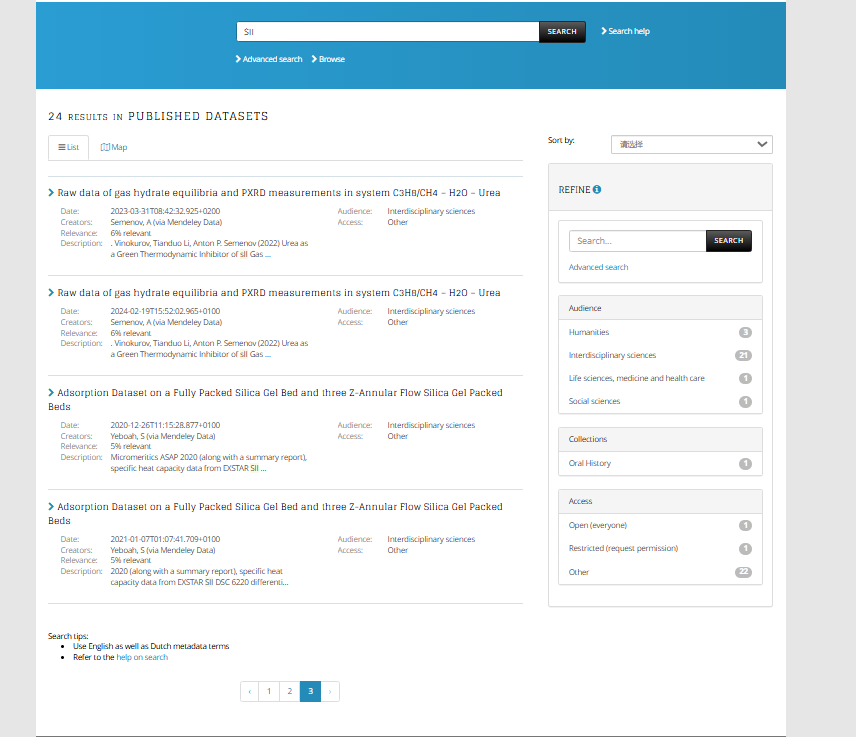


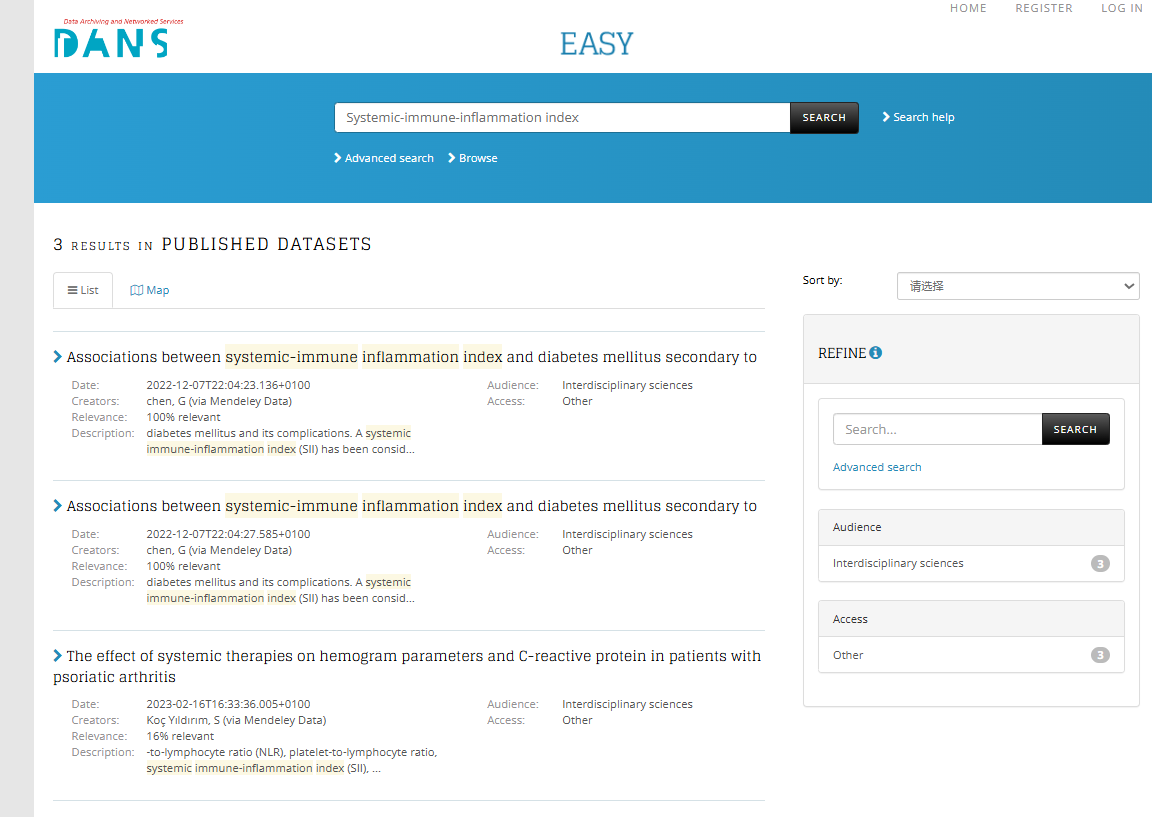


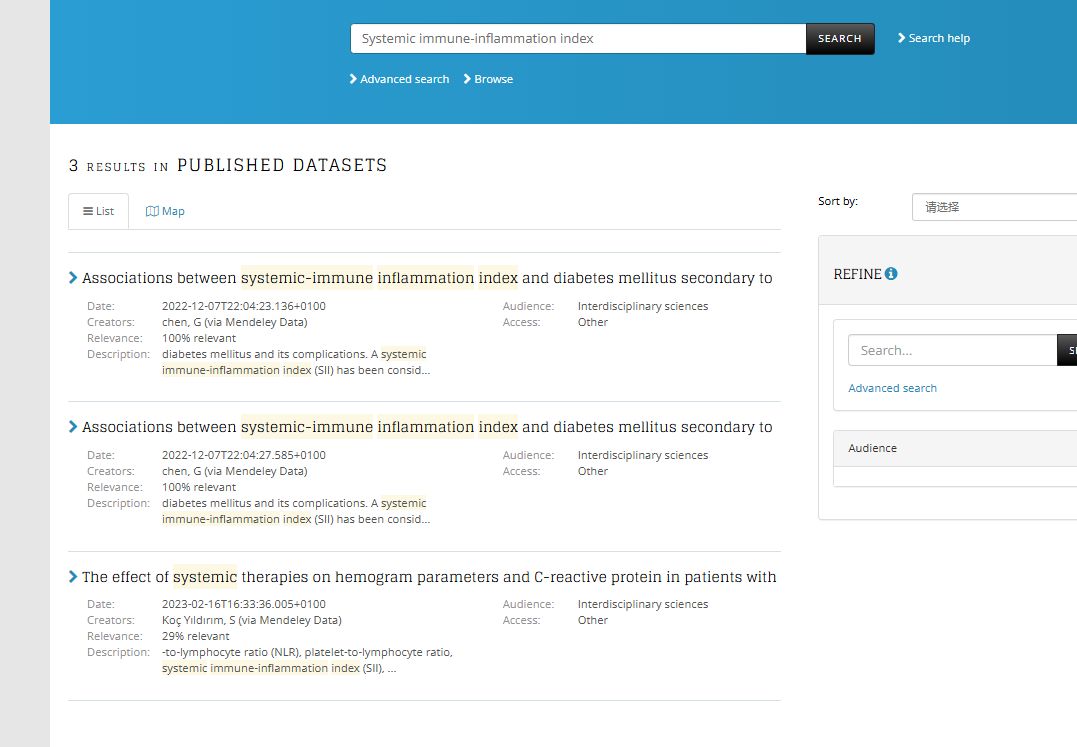


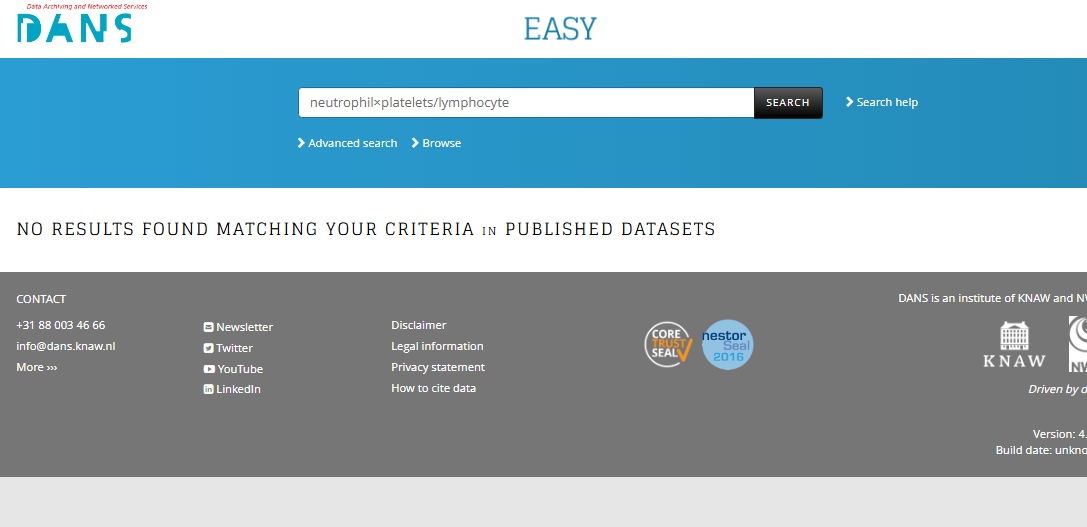

Supplement: Supplementary file 1 [file Table_1.docx]
